# Synthesis and in vitro antibacterial, antifungal, anti-proliferative activities of novel adamantane-containing thiazole compounds

**DOI:** 10.1038/s41598-022-25390-0

**Published:** 2022-12-06

**Authors:** Eman T. Warda, Mahmoud B. El-Ashmawy, El-Sayed E. Habib, Mohammed S. M. Abdelbaky, Santiago Garcia-Granda, Subbiah Thamotharan, Ali A. El-Emam

**Affiliations:** 1 Department of Medicinal Chemistry, Faculty of Pharmacy, Mansoura University, Mansoura, 35516 Egypt; 2 Department of Microbiology and Immunology, Faculty of Pharmacy, Mansoura University, Mansoura, 35516 Egypt; 3 Department of Physical and Analytical Chemistry, Faculty of Chemistry, Oviedo University-CINN, 33006 Oviedo, Spain; 4 Biomolecular Crystallography Laboratory, Department of Bioinformatics, School of Chemical and Biotechnology, SASTRA Deemed University, Thanjavur, 613 401 India

**Keywords:** Cancer, Computational biology and bioinformatics, Drug discovery, Microbiology, Chemistry

## Abstract

A series of (*Z*)-*N*-(adamantan-1-yl)-3,4-diarylthiazol-2(3*H*)-imines (**5a-r**) was synthesized via condensation of 1-(adamantan-1-yl)-3-arylthioureas (**3a-c**) with various aryl bromomethyl ketones (**4a-f**). The structures of the synthesized compounds were characterized by ^1^H NMR, ^13^C NMR and by X-ray crystallography. The in vitro inhibitory activities of the synthesized compounds were assessed against a panel of Gram-positive and Gram-negative bacteria, and pathogenic fungi. Compounds **5c**, **5g**, **5l**, **5m**, and **5q** displayed potent broad-spectrum antibacterial activity, while compounds **5a** and **5o** showed activity against the tested Gram-positive bacteria. Compounds **5b**, **5l** and **5q** displayed potent antifungal activity against *Candida albicans*. In addition, the synthesized compounds were evaluated for anti-proliferative activity towards five human tumor cell lines. The optimal anti-proliferative activity was attained by compounds **5e** and **5k** which showed potent inhibitory activity against all the tested cell lines. Molecular docking analysis reveals that compounds **5e** and **5k** can occupy the positions of NAD cofactor and the histone deacetylase inhibitor EX527 at the active site of SIRT1 enzyme.

## Introduction

**Figure 1:**
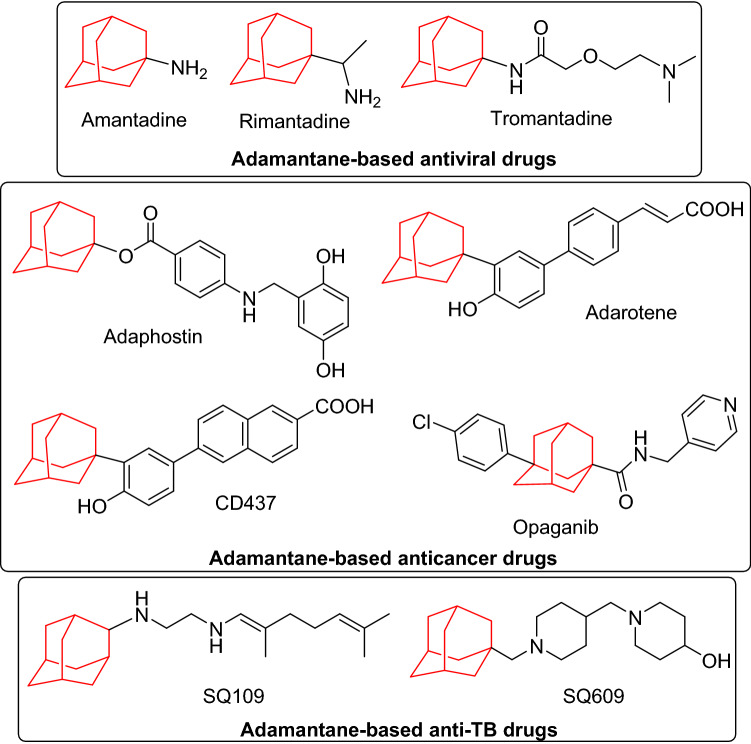
Adamantane-based chemotherapeutic drugs and drug candidates.

Adamantane cage is an interesting core of several drugs possessing diverse pharmacological properties^1–4^. The chemotherapeutic effectiveness of adamantane-based compounds was early explored after the discovery of amantadine^5,6^ and rimantadine^7^ as efficient medications for the control of Influenza A viral infections. Tromantadine was also developed as potent antiviral drug for the treatment of skin infection caused by herpes simplex virus^8^. Adamantane cage constitutes an important moiety of currently employed anticancer drugs. Adaphostin is a tyrosine kinase inhibitor that displays anti-proliferative activity in leukemia, non-small cell lung-cancer, and prostate cancer^9^. Adarotene, a IκB kinase-β inhibitor, was also developed as potent anticancer drug for the treatment of lymphoma, leukemia and prostate cancer^10^. The adamantane-based synthetic retinoid CD437 was also reported as a promising anticancer agent acting by inhibition of DNA polymerase^11^, while Opaganib is a recently approved adamantane-based anticancer drug for the treatment of advanced solid tumors^12^. Opaganib induces its anticancer activity via inhibition of sphingosine kinase (SK). Recently, opaganib proved to be a promising candidate for the treatment of severe COVID-19 pneumonia^13^. Other adamantane-based derivatives have demonstrated potent activity against pathogenic bacteria, mycobacteria and fungi. The adamantane-diamine hybrid derivative SQ109 was discovered for treatment of drug-resistant tuberculosis^14^, while the structurally-related dipiperidine analogue SQ609 was developed as a lead compound with potent action against *Mycobacterium tuberculosis*^15^ (Fig. 1).

**Figure 2:**
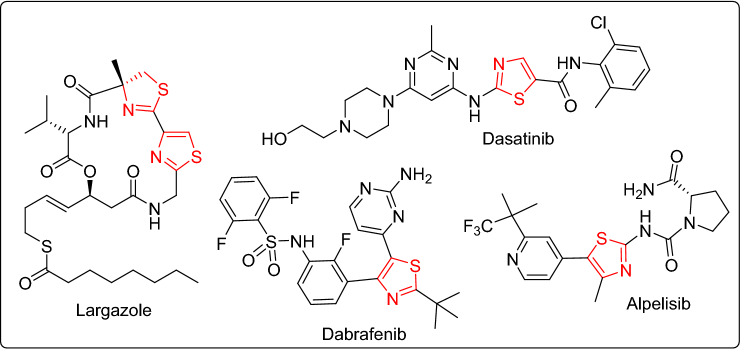
Thiazole-based anticancer drugs.

On the other hand, thiazole heterocycle represents the bioactive core of numerous drugs^16–18^. Several thiazole-based compounds were found to be effective chemotherapeutic agents with anticancer^19,20^, antifungal^21–23^, antibacterial^24,25^, antiviral^26^ and anti-leishmanial^27^ activities. The thiazole-based natural product largazole was discovered as potent anticancer drug acting via inhibition of histone deacetylases (HDACs)^28,29^. Dasatinib^30^, dabrafenib^31^ and alpelisib^32^ are currently-used drugs for the treatment of acute lymphoblastic leukemia, advanced melanoma and breast cancer, respectively (Fig. 2). Besides, the thiazole-based natural products patellamide A^33^, ixabepilone^34^ and epothilones^35^ are clinically useful for the treatment of resistant prostate cancer, metastatic breast cancer, ovarian and rectal cancers.

In light of the previously reported observations and as a continuation of an ongoing interest in the chemotherapeutic activities of adamantane-based derivatives^36–38^, we report herein the synthesis, characterization, and biological evaluation of a series of adamantane-linked thiazole derivatives as potential antibacterial, antifungal and anti-proliferative agents.

## Results and discussion

### Chemical synthesis

**Figure 3:**
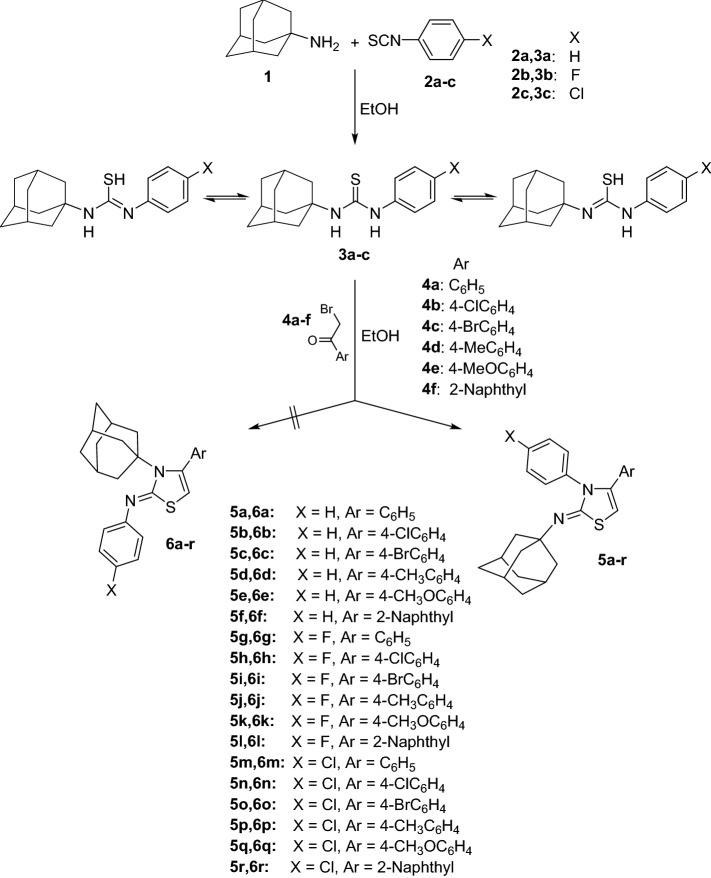
Synthetic outline of the target compounds **5a-r**.

Adamantan-1-amine **1** was allowed to react with phenyl isothiocyanate **2a**, 4-fluorophenyl isothiocyanate **2b** and 4-chlorophenyl isothiocyanate **2c** via heating in ethanol under reflux for 4 h to yield the corresponding 1-(adamantan-1-yl)-3-arylthiourea derivatives **3a-c** in good yields^39–41^. The thiourea derivatives **3a-c** were subsequently reacted with different aryl bromomethyl ketones **4a-f** via prolonged heating in ethanol followed by addition of sodium acetate to yield the corresponding (*Z*)-*N*-(adamantan-1-yl)-3,4-diarylthiazol-2(3*H*)-imine derivatives **5a-r** rather than the isomeric (*Z*)-3-(adamantan-1-yl)-4,*N*-diarylthiazol-2(3*H*)-imines **6a-r**. The structures of the diarylthiazol-2(3*H*)-imines **5a-r** were assigned based on ^1^H NMR, ^13^C NMR and elemental analysis. Single crystal X-ray analysis of compounds **5d** and **5f.** was also performed to assign their configuration and to exclude the formation of compounds **6a-r** (Fig. 3). The ^1^H NMR spectra of compounds **5a-r** showed the adamantane protons (15H) as three singlets (condensed multiplets) at *δ* 1.65–1.76 (6H), 1.84–1.99 (6H) and 2.06–2.17 (3H) ppm. The thiazole CH protons were shown as distinguished singlets at *δ* 5.78–6.04 ppm. Meanwhile, the ^13^C NMR spectra of compounds **5a-r** showed the adamantane carbons as four characteristic peaks at *δ* 29.64–30.12, 36.60–37.08, 40.63–41.17 and 53.49–54.19 ppm. In addition, the C5, C4 and C2 carbons of the thiazole ring were shown at *δ* 96.11–99.09, 148.47–150.28 and 158.89–160.08 ppm, respectively. The aromatic protons and carbons and their substituents were properly shown in their ^1^H NMR and ^13^C NMR spectra (see “Materials and methods” section).

The molecular and crystal structures of compounds **5d** and **5f.** were determined by single crystal X-ray analysis to explore the configuration of compounds **5a-r** and to exclude the formation of compounds **6a-r**. The crystal data and refinement parameters of the compounds are summarized in Table S1.

**Figure 4:**
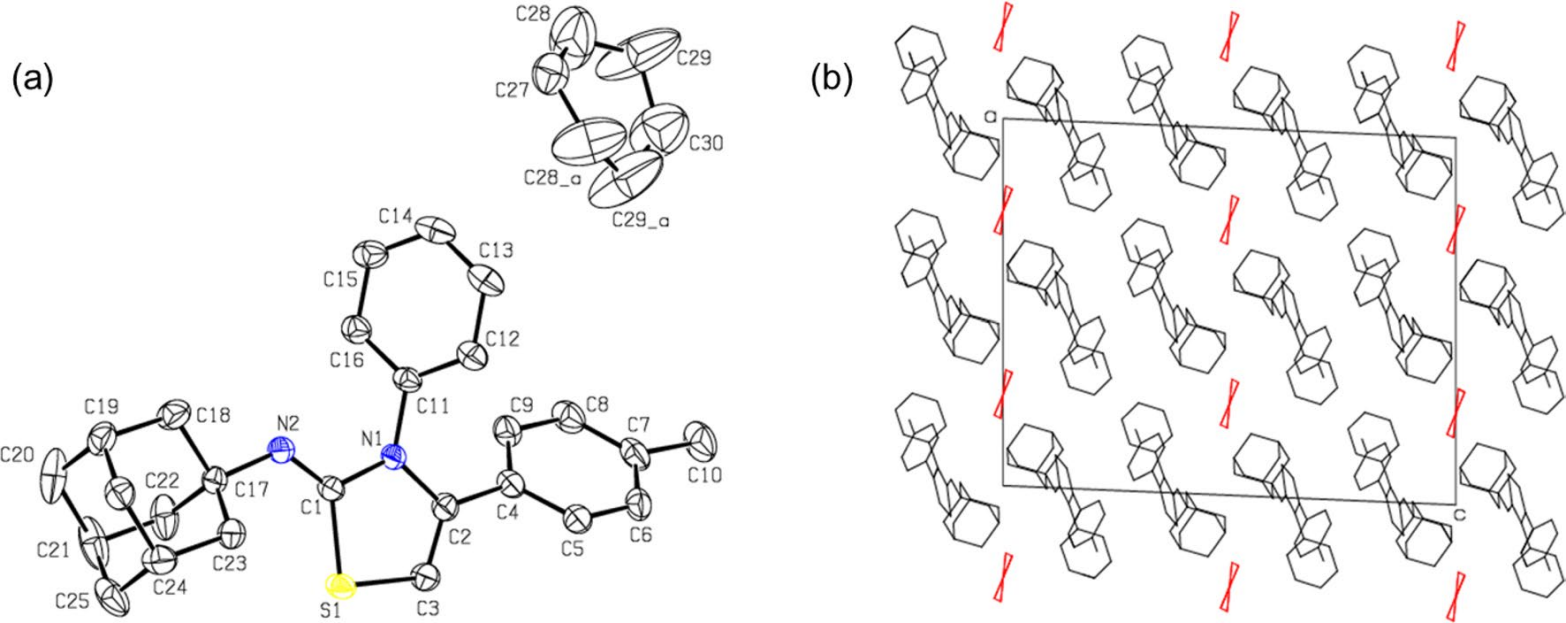
(**a**) ORTEP plot of compound **5d** and a cyclohexane solvent in the crystalline lattice (symmetry element (**a**) − x + 1/2, y, − z). The displacement ellipsoid is drawn at the 50% probability level along atom-labelling scheme and (**b**) columnar packing mode of compound **5d** along the crystallographic *ac* plane. The lattice cyclohexane solvent is shown in red. All H atoms have been omitted for the sake of clarity.

Compound **5d** crystallizes in the monoclinic system with the space group I2/a. The asymmetric unit contains one molecule of the compound as cyclohexane solvate (Fig. 4a). The atoms C27 and C30 of the cyclohexane molecule lie on two-fold axis. The six-membered rings of the adamantane moiety exhibit a chair conformation, whereas a cyclohexane solvent adopts twist-boat conformation. The central thiazole ring is oriented at an angle of 47.30° with the mean plane of the *p*-tolyl group, whereas the same thiazole unit makes 53.66° angle with the phenyl ring attached at the third position of the thiazole ring. In the solid state, molecules of **5d** and cyclohexane solvent observed in the crystal lattice forming in a columnar fashion (Fig. 4b) as observed in many adamantane derivatives. No classical hydrogen bonds observed in the crystal structure. However, a weak intermolecular C–H…π (involving the tolyl proton, H6 and centroid of the thiazole ring; C6–H6…Cg1 = 149°; H6…Cg1 = 2.72 Å, C6…Cg1 = 3.572(3); symmetry element: − x, y + 1/2, − z + 1/2).

**Figure 5:**
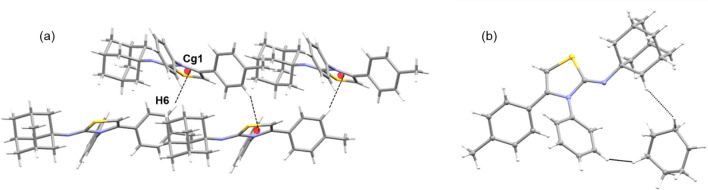
(**a**) Molecular chain formed by a weak C–H…π interaction in the solid state of compound and (**b**) cyclohexane…adamantane/phenyl interactions in compound **5d**.

This interaction links the molecules into a chain that runs parallel to the crystallographic *b* axis (Fig. 5a). The cyclohexane solvent establishes a pair of weak H…H contacts with the protons of the adamantane and phenyl moieties (Fig. 5b).

**Figure 6:**
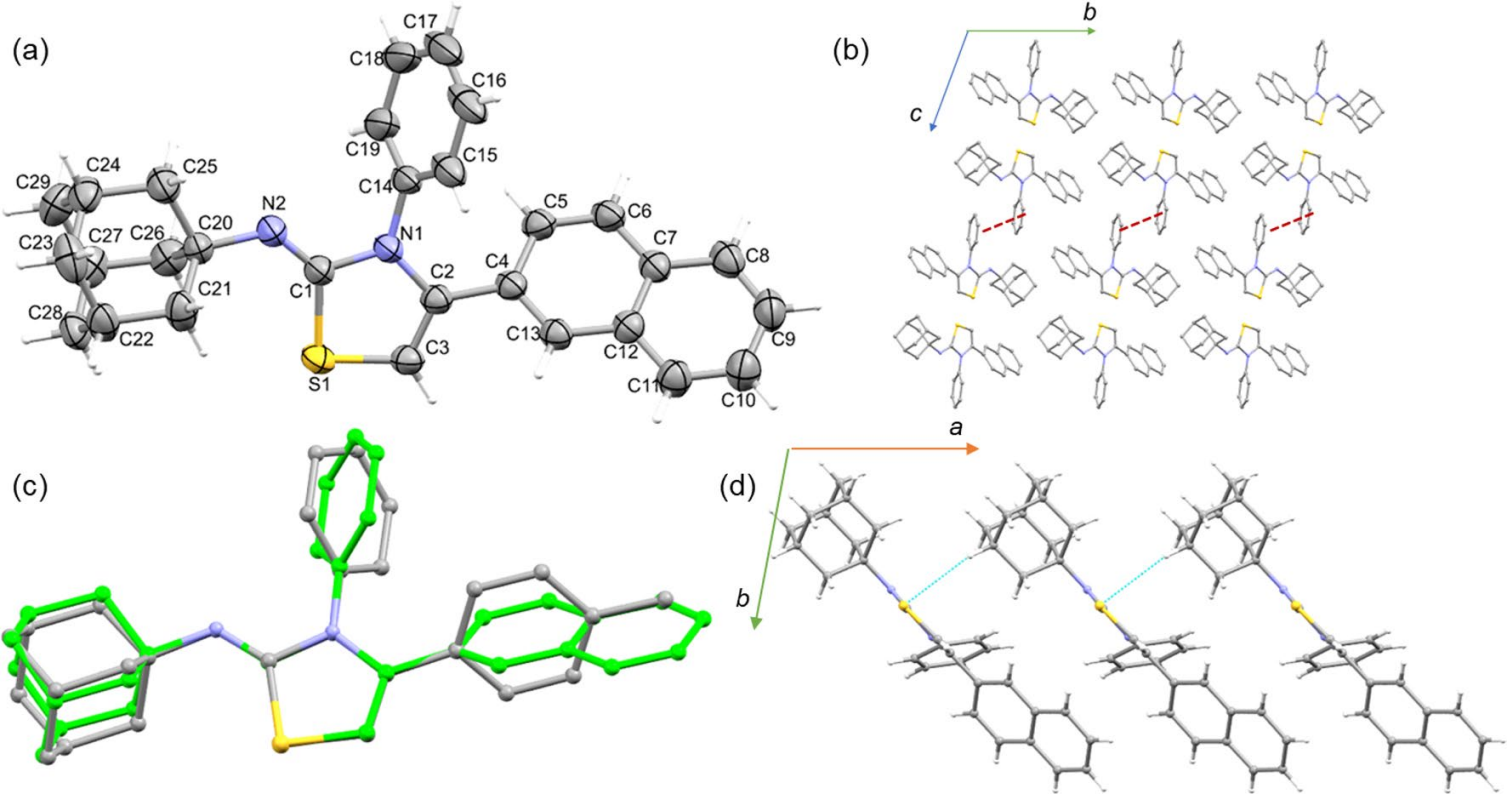
(**a**) ORTEP representation of compound **5f.** with atom-labelling scheme, (**b**) crystal packing of compound **5f.** projected along *bc* plane and the π-stacking interaction between phenyl rings is shown as red dashed lines, (**c**) structural superposition of compounds **5d** and **5f.** (cyclohexane solvent has been omitted for clarity) and (**d**) molecular chain is generated via a weak intermolecular C–H…S interaction.

Compound **5f.** crystallizes in the triclinic system with the P-1 space group. The asymmetric unit contains one molecule (Fig. 6a). This compound has a naphthalene ring instead of the *p*-tolyl position in compound **5d**. In compound **5f.**, the central thiazole ring is oriented at an angle of 42.03° with the mean plane of the naphthalene ring. However, the mean plane of the phenyl ring makes 60.25° with the thiazole core.

The six-membered rings of the adamantane cage exhibits chair conformation as observed in related structures. In the crystalline state, the molecules pack in a columnar fashion (Fig. 6b). Figure 6c shows the structural overlay between **5d** and **5f.** structures and one can see from this figure that the central thiazole and adamantane cage overlap well and the aryl rings show twist orientations. The crystal structure of **5f.** is stabilized primarily by a weak π-stacking interaction formed between phenyl rings in adjacent columns (centroid–centroid = 3.848 (2), symmetry element: 1-x, 1-y, 1-z). In addition, a weak C–H…S interaction (C27-H27…S1 = 161°, H27…S1 = 3.02 Å and C27…S1 = 3.959 Å; symmetry element: x-1, y, z) links the neighboring molecules into a chain that runs parallel to the crystallographic *a* axis as shown in Fig. 6d.

### In vitro antibacterial and antifungal activities

The in vitro antibacterial and antifungal activity of newly designed compounds **5a-r** was assessed against a panel of standard pathogenic strains of the Institute of Fermentation of Osaka (IFO) namely; *Staphylococcus aureus* IFO 3060 (***SA***), *Bacillus subtilis* IFO 3007 (***BS***), *Micrococcus luteus* IFO 3232 (***ML***) (Gram-positive bacteria), *Escherichia coli* IFO 3301 (***EC***), *Pseudomonas aeruginosa* IFO 3448 (***PA***) (Gram-negative bacteria ) and the pathogenic fungi *Candida albicans* IFO 0583 (***CA***), *Aspergillus oryzae* IFO 4177 (***AO***) and *Aspergillus niger* IFO 4414 (***AN***). The initial screening was carried out using the semi-quantitative agar disc-diffusion method using Müller-Hinton agar medium^42^.

The results of the initial antimicrobial screening of compounds **5a-r** (200 µg/disc); the antibacterial antibiotics Ampicillin trihydrate, Ciprofloxacin (100 µg/disc) and the antifungal drug Fluconazole (100 µg/disc) are shown in Table 1. The results showed varying degrees of inhibition against the tested microorganisms. In general, potent antibacterial activity was displayed by compounds **5a**, **5c**, **5e**, **5g**, **5h**, **6j**, **5l**, **5m**, **5n**, **5o**, **5p** and **5q**, which produced growth inhibition zones ≥ 18 mm against one or more of the tested bacterial strains. Meanwhile, compounds **5b**, **5d**, **5f.**, **5i**, **5k** and **5r** displayed moderate antibacterial activity (growth inhibition zones 14–17 mm). In addition, the Gram-positive bacteria *Staphylococcus aureus*, *Bacillus subtilis* and to a lesser extent *Micrococcus luteus* are considered the most sensitive among the tested bacterial strains. The inhibitory activity against the tested Gram-negative bacteria was generally lower than the Gram-positive bacteria; compounds **5c**, **5g**, **5l**, **5m** and **5q** showed potent inhibitory activity against *Escherichia coli*, while only compounds **5c**, **5l** and **5m** were strongly active against *Pseudomonas aeruginosa*. The optimum antibacterial activity was attained by compounds **5c**, **5g**, **5l**, **5m** and **5q** which displayed potent broad spectrum antibacterial activity.

**Table 1:** Antibacterial and antifungal activities of compounds **5a-r** (200 μg/8 mm disc), the broad-spectrum antibacterial drugs Ampicillin trihydrate, Ciprofloxacin and the antifungal drug Fluconazole (100 μg/8 mm disc) against the Gram-positive bacteria *Staphylococcus aureus* IFO 3060 (***SA***), *Bacillus subtilis* IFO 3007 (***BS***) and *Micrococcus luteus* IFO 3232 (***ML***), the Gram-negative bacteria *Escherichia coli* IFO 3301 (***EC***) and *Pseudomonas aeruginosa* IFO 3448 (***PA***) and the pathogenic fungi *Candida albicans* IFO 0583 (***CA***), *Aspergillus oryzae* IFO 4177 (***AO***) and *Aspergillus niger* IFO 4414 (***AN***).


Comp. No	X	Ar	Diameter of Growth Inhibition Zone (mm)*							
			*SA*	*BS*	*ML*	*EC*	*PA*	*CA*	*AO*	*AN*
**5a**	H	C_6_H_5_	**25**	**26**	**20**	12	-	-	-	-
**5b**	H	4-ClC_6_H_4_	14	17	10	-	-	**18**	-	-
**5c**	H	4-BrC_6_H_4_	**28**	**23**	**24**	**25**	**19**	10	-	-
**5d**	H	4-CH_3_C_6_H_4_	17	14	15	14	12	-	-	-
**5e**	H	4-CH_3_OC_6_H_4_	**18**	15	15	-	-	-	-	-
**5f.**	H	2-Naphthyl	14	15	12	11	10	-	-	-
**5g**	F	C_6_H_5_	**21**	17	**21**	**21**	15	-	13	-
**5h**	F	4-ClC_6_H_4_	**18**	15	17	16	14	12	10	-
**5i**	F	4-BrC_6_H_4_	12	14	12	12	11	-	-	-
**5j**	F	4-CH_3_C_6_H_4_	**18**	17	15	15	12	14	-	-
**5k**	F	4-CH_3_OC_6_H_4_	14	14	12	14	10	17	-	-
**5l**	F	2-Naphthyl	**25**	**28**	**22**	**22**	**18**	**18**	-	-
**5m**	Cl	C_6_H_5_	**22**	**20**	**18**	**26**	**20**	-	-	-
**5n**	Cl	4-ClC_6_H_4_	**18**	17	15	15	12	-	-	-
**5o**	Cl	4-BrC_6_H_4_	**26**	**22**	**18**	14	12	-	-	-
**5p**	Cl	4-CH_3_C_6_H_4_	**18**	15	10	12	-	-	-	-
**5q**	Cl	4-CH_3_OC_6_H_4_	**24**	**22**	**22**	**18**	15	**19**	14	-
**5r**	Cl	2-Naphthyl	14	10	12	-	-	-	-	
**Ampicillin trihydrate**	**28**	**30**	**25**	**24**	**22**	**NT**	**NT**	**NT**		
**Ciprofloxacin**	**34**	**38**	**32**	**38**	**36**	NT	NT	NT		
**Fluconazole**	NT	NT	NT	NT	NT	**21**	**22**	**24**		

*NT* not tested.

*-Inactive (inhibition zone < 10 mm).

Significant values are in bold.

The antifungal activity of the compounds **5a-r** was generally lower than their antibacterial activity. Compounds **5b**, **5l** and **5q** displayed high activity against the yeast-like pathogenic fungus *Candida albicans*, while compounds **5j** and **5k** were moderately active and compounds **5c** and **5h** exhibited marginal activity (growth inhibition zones 10–13 mm). Compounds **5g**, **5h** and **5q** were almost weakly against *Aspergillus oryzae*, and all the compounds were totally inactive against *Aspergillus niger*.

In general, the antibacterial and antifungal potency and spectrum are greatly influenced by the nature of the aryl groups at positions 3 and 4 of the core thiazole nucleus. In the 3-phenyl series **5a-f**, the 3,4-diphenyl analogue **5a** displayed potent antibacterial activity against the tested Gram-positive bacteria and lacked activity against the tested Gram-negative bacteria and pathogenic fungi. Replacement of the 4-phenyl group with 4-(4-chlorophenyl) substituent (compound **5b**) deteriorated the antibacterial activity and greatly enhanced the activity against *Candida albicans*. Meanwhile, the 4-(*p*-tolyl) **5d** and 4-(2-naphthyl) **5f.** analogues retained moderate activity against the tested Gram-positive bacteria, with only marginal activity against the tested Gram-negative bacteria, and no antifungal activity. In addition, the 4-(4-methoxyphenyl) derivative **5e** displayed good activity against *Staphylococcus aureus* and moderate activity against *Bacillus subtilis* and *Micrococcus luteus* with no activity against the tested Gram-negative bacteria and fungi. The optimum antibacterial activity was attained in the 4-bromophenyl analogue **5c** which displayed excellent broad-spectrum antibacterial activity.

In the 3-(4-fluorophenyl) series **5g-l**, the 4-phenyl, 4-(4-chlorophenyl) and 4-(*p*-tolyl) derivatives **5g**, **5h** and **5j** showed marked broad-spectrum antibacterial activity and weak antifungal activity against *Candida albicans* and/or *Aspergillus oryzae*. Unlike the antimicrobial activity in the 3-phenyl derivatives **5a-f**, the antibacterial activity of the 4-bromophenyl analogue **5i** was found inferior compared to the majority of the 3-(4-fluorophenyl) derivatives **5g-l**, and with no antifungal activity. Meanwhile, the 4-(*p*-tolyl) **5j** and 4-(4-methoxyphenyl) **5k** analogues showed almost moderate or weak antibacterial activity and moderate activity against *Candida albicans*. In contrary to the antimicrobial activity of the 3-phenyl derivatives **5a-f**, the 4-(2-naphthyl) analogue **5l** displayed excellent broad-spectrum antibacterial activity in addition to potent antifungal activity against *Candida albicans*.

In the 3-(4-chlorophenyl) series **5m-r**, the optimum antibacterial activity was shown by the 4-phenyl **5m** and 4-(4-methoxyphenyl) **5q** derivatives which almost displayed potent broad-spectrum antibacterial activity, while the 4-(4-bromophenyl) derivative **5o** retained potent and almost weak activity against the tested Gram-positive and Gram-negative bacteria, respectively. The antibacterial activity of the 4-(4-chlorophenyl) **5n** and 4-(*p*-tolyl) **5p** derivatives was found similar, but to lower extent, to the activity of the 4-(4-bromophenyl) derivative **5o**. The 4-(2-naphthyl) analogue **5r** only retained moderate or marginal activity against the tested Gram-positive bacteria. Potent and moderate antifungal activity against *Candida albicans* and *Aspergillus oryzae*, respectively, was displayed by the 4-(4-methoxyphenyl) **5q** derivative.

Taken together, the broad spectrum antibacterial activity was exhibited by monohalogenated derivatives rather than the dihalogenated ones. This is illustrated by the activity **5c**, **5l**, **5m** and **5q**. The same results were clearly demonstrated by the antifungal activity of compounds **5b**, **5l** and **5q**. Upon comparing the antibacterial activity of the two isomeric structures **5b** and **5m**, it has been discovered that the halogen substitution pattern is more beneficial at the N-3 position than the C-4 position.

The minimal inhibitory concentrations (MIC) and the minimal biocidal concentrations (MBC, bactericidal and fungicidal) for seven of the most active compounds (**5a**, **5c**, **5g**, **5l**, **5m**, **5o** and **5q**) against the same microorganisms used in the primary screening were carried out using the micro-dilution susceptibility method in Müller-Hinton Broth^43–45^. The MIC and MBC values of compounds **5a**, **5c**, **5g**, **5l**, **5m**, **5o**, **5q**, Ampicillin trihydrate, Ciprofloxacin and Fluconazole are depicted in Table 2. The MIC of the tested compounds were in accordance with the results obtained in the primary screening. According to the antimicrobial standards, an agent is usually considered as bactericidal or fungicidal if the MBC/MIC ratio is not more than 4^46^. The MBC/MIC ratio for the tested compounds (Table 2) were found to be around 2. According to the MBC/MIC values of the tested compounds, it could be concluded that the (*Z*)-*N*-(adamantan-1-yl)-3,4-diarylthiazol-2(3*H*)-imine derivative **5a**, **5c**, **5g**, **5l**, **5m**, **5o** and **5q** could be considered as potential antibacterial candidates for further studies. Though these compounds are of inferior activity compared to the reference drugs, they have displayed MBC/MIC ratio equivalent to that of the references.

**Table 2:** The Minimal Inhibitory Concentrations (MIC) and Minimal Biocidal Concentrations (MBC, bactericidal and fungicidal) of compounds **5a**, **5c**, **5g**, **5l**, **5m**, **5o** and **5q** in comparison with the broad spectrum antibacterial drugs Ampicillin trihydrate, Ciprofloxacin and the antifungal drug Fluconazole against the Gram-positive bacteria *Staphylococcus aureus* IFO 3060 (*SA*), *Bacillus subtilis* IFO 3007 (*BS*) and *Micrococcus luteus* IFO 3232 (*ML*), the Gram-negative bacteria *Escherichia coli* IFO 3301 (*EC*) and *Pseudomonas aeruginosa* IFO 3448 (*PA*) and the pathogenic fungus *Candida albicans* IFO 0583 (*CA*).

Comp. No	MIC/MBC (μg/mL)*					
	*SA*	*BS*	*ML*	*EC*	*PA*	*CA*
**5a**	3.25/7.20	3.5/8.25	7.0/15.5	ND/ND	ND/ND	ND/ND
**5c**	2.0/5.20	4.0/7.50	3.0/6.20	6.5/11.50	10.25/ND	ND/ND
**5g**	6.50/10.25	ND/ND	6.50/12.0	8.0/16.20	ND/ND	ND/ND
**5l**	3.25/8.50	2.25/5.0	4.0/9.50	7.20/15.50	ND/ND	8.50/16.20
**5m**	6.0/12.50	8.50/15.50	ND/ND	4.25/8.75	8.50/ND	ND/ND
**5o**	3.20/8.25	3.75/8.0	ND/ND	ND/ND	ND/ND	ND/ND
**5q**	3.50/8.20	3.75/7.50	4.20/7.80	ND/ND	ND/ND	7.25/13.50
**Ampicillin**	2.0/3.5	1.0/3.0	2.5/4.0	ND/ND	ND/ND	ND/ND
**Ciprofloxacin**	0.75/1.50	0.50/1.0	1.0/2.0	0.25/1.0	ND/ND	ND/ND
**Fluconazole**	ND/ND	ND/ND	ND/ND	ND/ND	ND/ND	6.25/12.0

*ND* not determined.

*Data shown are the mean values of three experiments.

The adamantane-linked thiazoles **5a**, **5c**, **5l** and **5o** which displayed the best antibacterial activity against Gram-positive bacteria were selected for further assessment of their bacterial biofilm inhibitory activity using the crystal violet staining method^47^. Bacterial biofilms are clusters of bacteria that are attached to a surface and/or to each other and embedded in a self-produced matrix. The biofilm matrix mainly consists of proteins and polysaccharides. In addition to the protection offered by the matrix, bacteria in biofilms can employ several survival strategies to evade the host defense systems^48,49^. The minimal biofilm inhibitory concentrations (IC_50_) of compounds **5a**, **5c**, **5l**, **5o** and the broad-spectrum antibiotic Erythromycin towards the Gram-positive bacteria *Staphylococcus aureus* and *Micrococcus luteus* are shown in Table 3. The IC_50_ (the lowest concentrations of tested compounds that exhibited 50% inhibition on the biofilm formation) ranged from 1.6 to 2.5 and 3.4 to 7.4 μg/mL against *Staphylococcus aureus* and *Micrococcus luteus*, respectively.

**Table 3:** Anti-biofilm activity (IC_50_, μg/mL) of compounds **5a**, **5c**, **5l**, **5o** and the broad-spectrum antibiotic Erythromycin against *Staphylococcus aureus* IFO 3060 (*SA*) and *Micrococcus luteus* IFO 3232 (*ML*).

Comp. No	Anti-biofilm activity (μg/mL)*	
	*SA*	*ML*
**5a**	2.4 ± 0.12	4.8 ± 0.20
**5c**	1.6 ± 0.25	6.5 ± 0.25
**5l**	2.5 ± 0.15	3.4 ± 0.15
**5o**	2.0 ± 0.15	7.4 ± 0.21
**Erythromycin**	0.45 ± 0.15	0.62 ± 0.21

*Results are the mean values of three experiments ± SD.

### In vitro anti-proliferative activity

The in vitro anti-proliferative activity of the adamantane-linked thiazoles **5a-r** was assessed against five human tumor cell lines namely; PC-3 (human prostate cancer), HCT-116 (human colorectal cancer), HepG-2 (human hepatocellular cancer), HeLa (human cervical cancer) and MCF-7 (human breast cancer) using the 3-[4,5-dimethylthiazoyl-2-yl]-2,5-diphenyltetrazolium bromide (MTT) colorimetric assay^50^. The obtained results, together with that of the anticancer drug doxorubicin, are shown in Table 4.

**Table 4:** In vitro anti-proliferative activity of compounds **5a-r** and the anticancer drug Doxorubicin toward human prostate cancer (PC-3), colorectal carcinoma (HCT-116), hepatocellular carcinoma (HepG-2), epithelioid cervical carcinoma (HeLa) and mammary gland breast cancer (MCF-7) cell lines.


Comp. No	X	Ar	IC_50_ (µM)^a^				
			PC-3	HCT-116	HepG-2	HeLa	MCF-7
**5a**	H	C_6_H_5_	67.26 ± 3.6	59.23 ± 3.3	48.32 ± 2.9	41.81 ± 2.4	51.26 ± 2.9
**5b**	H	4-ClC_6_H_4_	87.82 ± 4.1	85.51 ± 4.4	65.29 ± 3.4	71.48 ± 3.7	78.62 ± 3.9
**5c**	H	4-BrC_6_H_4_	78.97 ± 3.9	80.25 ± 4.2	56.43 ± 3.2	83.86 ± 4.0	72.31 ± 3.7
**5d**	H	4-CH_3_C_6_H_4_	53.19 ± 2.9	58.63 ± 3.2	38.24 ± 2.5	49.83 ± 2.7	45.66 ± 2.6
**5e**	H	4-CH_3_OC_6_H_4_	**12.85 ± 1.0**	**11.96 ± 0.9**	**16.30 ± 1.4**	**9.87 ± 0.7**	**6.90 ± 0.4**
**5f.**	H	2-Naphthyl	> 100	94.26 ± 4.8	81.62 ± 4.2	> 100	86.49 ± 4.4
**5g**	F	C_6_H_5_	**16.73 ± 1.3**	28.27 ± 2.1	**20.87 ± 1.6**	30.67 ± 2.1	**19.64 ± 1.4**
**5h**	F	4-ClC_6_H_4_	**23.56 ± 1.9**	39.74 ± 2.5	**22.07 ± 1.8**	**18.11 ± 1.5**	32.78 ± 2.2
**5i**	F	4-BrC_6_H_4_	> 100	> 100	88.57 ± 4.5	> 100	91.18 ± 4.8
**5j**	F	4-CH_3_C_6_H_4_	90.04 ± 4.6	73.86 ± 3.9	70.28 ± 3.8	52.53 ± 2.9	66.28 ± 3.5
**5k**	F	4-CH_3_OC_6_H_4_	**9.71 ± 0.8**	**6.47 ± 0.5**	**11.27 ± 0.9**	**7.09 ± 0.5**	**3.51 ± 0.2**
**5l**	F	2-Naphthyl	> 100	89.05 ± 4.5	77.21 ± 4.0	93.62 ± 4.7	84.03 ± 4.3
**5m**	Cl	C_6_H_5_	33.59 ± 2.2	36.42 ± 2.3	29.53 ± 2.2	**21.59 ± 1.8**	26.04 ± 1.9
**5n**	Cl	4-ClC_6_H_4_	40.70 ± 2.4	46.11 ± 2.6	31.98 ± 2.3	25.65 ± 2.0	39.21 ± 2.5
**5o**	Cl	4-BrC_6_H_4_	61.39 ± 3.4	**24.50 ± 1.8**	47.38 ± 2.8	**15.28 ± 1.2**	**13.72 ± 1.2**
**5p**	Cl	4-CH_3_C_6_H_4_	55.86 ± 3.1	62.79 ± 3.5	43.65 ± 2.7	37.26 ± 2.3	57.39 ± 3.2
**5q**	Cl	4-CH_3_OC_6_H_4_	42.08 ± 2.6	**17.58 ± 1.4**	35.18 ± 2.5	**10.49 ± 0.9**	**8.53 ± 0.7**
**5r**	Cl	2-Naphthyl	75.45 ± 3.8	68.35 ± 3.7	51.79 ± 3.1	56.40 ± 3.3	63.54 ± 3.4
**Doxorubicin**			**8.87 ± 0.6**	**5.23 ± 0.3**	**4.50 ± 0.2**	**5.57 ± 0.4**	**4.17 ± 0.2**

^a^IC_50_ Values presented as the mean ± SD of three separate determinations.

Significant values are in bold.

Compounds **5a-r** displayed variable degrees of inhibitory activity against the tested cancer cell lines. In general, compounds **5e**, **5g**, **5h**, **5k**, **5o** and **5q** displayed potent anti-proliferative activity (IC_50_ < 25 μg/mL) against at least three of the tested cancer cell lines, compounds **5a**, **5d** and **5n** showed moderate activity (IC_50_ > 25 to 50 μg/mL), compounds **5b**, **5c**, **5j** and **5r** showed weak activity (IC_50_ > 50 to 75 μg/mL) while compounds **5f.**, **5i** and **5l** were inactive (IC_50_ > 75 μg/mL). In addition, the optimal anti-proliferative activity was attained by compounds **5e** and **5k** which showed excellent activity against all the tested cell lines.

The anti-proliferative potency was found to be mainly dependent on the nature of the aryl groups at positions 3 and 4 of the *N*-(adamantan-1-yl)-thiazol-2(3*H*)-imine core. Within the 3-phenyl derivatives **5a-f**, the 3,4-diphenyl analogue **5a** exhibited moderate activity against HepG-2 and HeLa cell lines with weak activity against PC-3, HCT-116 and MCF-7 cell lines. Replacement of the 4-phenyl group with 4-(4-chlorophenyl), 4-(4-bromophenyl) or 4-(*p*-tolyl) substituents greatly deteriorated the anti-proliferative activity against PC-3 and HCT-116 cell lines with almost weak or marginal activity against HepG-2, HeLa and MCF-7 cell lines. The 4-(4-methoxyphenyl) substituent (compound **5e**) is optimistic for anti-proliferative activity against all the tested cell lines. In contrary, a 4-(2-naphthyl) substituent (compound **5f.**) dramatically declined the anti-proliferative activity.

In the 3-(4-fluorophenyl) derivatives **5g-l**, the 4-phenyl analogue **5g** displayed potent activity against PC-3, HepG-2 and MCF-7 cell lines and moderate activity against HCT-116 and HeLa cell lines, while the 4-(4-chlorophenyl) analogue **5h** exhibited high activity against PC-3, HepG-2 and HeLa cell lines and moderate activity against HCT-116 and MCF-7 cell lines. In addition, the 4-(4-bromophenyl), 4-(*p*-tolyl) and 4-(2-naphthyl) derivatives **5i**, **5j** and **5l** were almost inactive against all the tested cell lines. The 4-(4-methoxyphenyl) analogue **5k** was found to the most active among the 3-(4-fluorophenyl) derivatives **5g-l** which resembles the anti-proliferative activity of Doxorubicin.

The anti-proliferative activity of the 3-(4-chlorophenyl) derivatives **5m-r** was rather lower than the 3-(4-fluorophenyl) derivatives **5g-l**. the 4-phenyl analogue **5m** displayed potent activity against HeLa cell lines and moderate activity against PC-3, HCT-116, HepG-2 and MCF-7 cell lines, while the 4-(4-chlorophenyl) analogue **5n** showed moderate activity against all the tested cell lines. In contrary to the activity of the 3-phenyl and 3-(4-fluorophenyl) derivatives (**5c** and **5i**), the 4-(4-bromophenyl) analogue **5o** showed potent activity against HCT-116, HeLa and MCF-7 cell lines, moderate activity against HepG-2 and week activity against PC-3 cell lines. As in case of the 3-phenyl and 3-(4-fluorophenyl) derivatives (**5e** and **5k**). the anti-proliferative activity of the 4-(4-methoxyphenyl) analogue **5q** was found to the most active among the 3-(4-chlorophenyl) derivatives **5m-r** as it in displayed potent activity against HCT-116, HeLa and MCF-7 cell lines, moderate activity against HepG-2 and week activity against PC-3 cell lines.

In view of the available results, it can be concluded that the presence of a 4-methoxyphenyl group at C-4 of the thiazole ring is optimal for the anti-proliferative activity, regardless of the nature of the C-3 substitution. Other C-4 substitutions diminish such activity, especially the 2-naphthyl group that impaired activity against all tested cell lines.

### Prediction of activity spectra and molecular docking studies

The online PASS (Prediction of Activity Spectra) structure–activity relationship tool^51^ was utilized to predict a suitable target of the designed (*Z*)-*N*-(adamantan-1-yl)-3,4-diarylthiazol-2(3*H*)-imine compounds **5a-r**. The PASS analysis results indicated that the predicted highest probability of biological activity (Pa) was for histone deacetylase (HDAC) SIRT1 enzyme (Table S2). The PASS prediction results are supported by the reported HDAC inhibitory activity of various adamantane^52–54^ and thiazole^28,29,55–59^ derivatives.

HDACs are capable of catalyzing the removal of *N*-acetyl group from acetylated lysine residues in histones or some non-histone proteins. The overexpression of HDACs has been linked to a variety of solid and hematological malignancies, neurological disorders, inflammatory diseases and metabolic disorders^60–62^.

**Figure 7:**
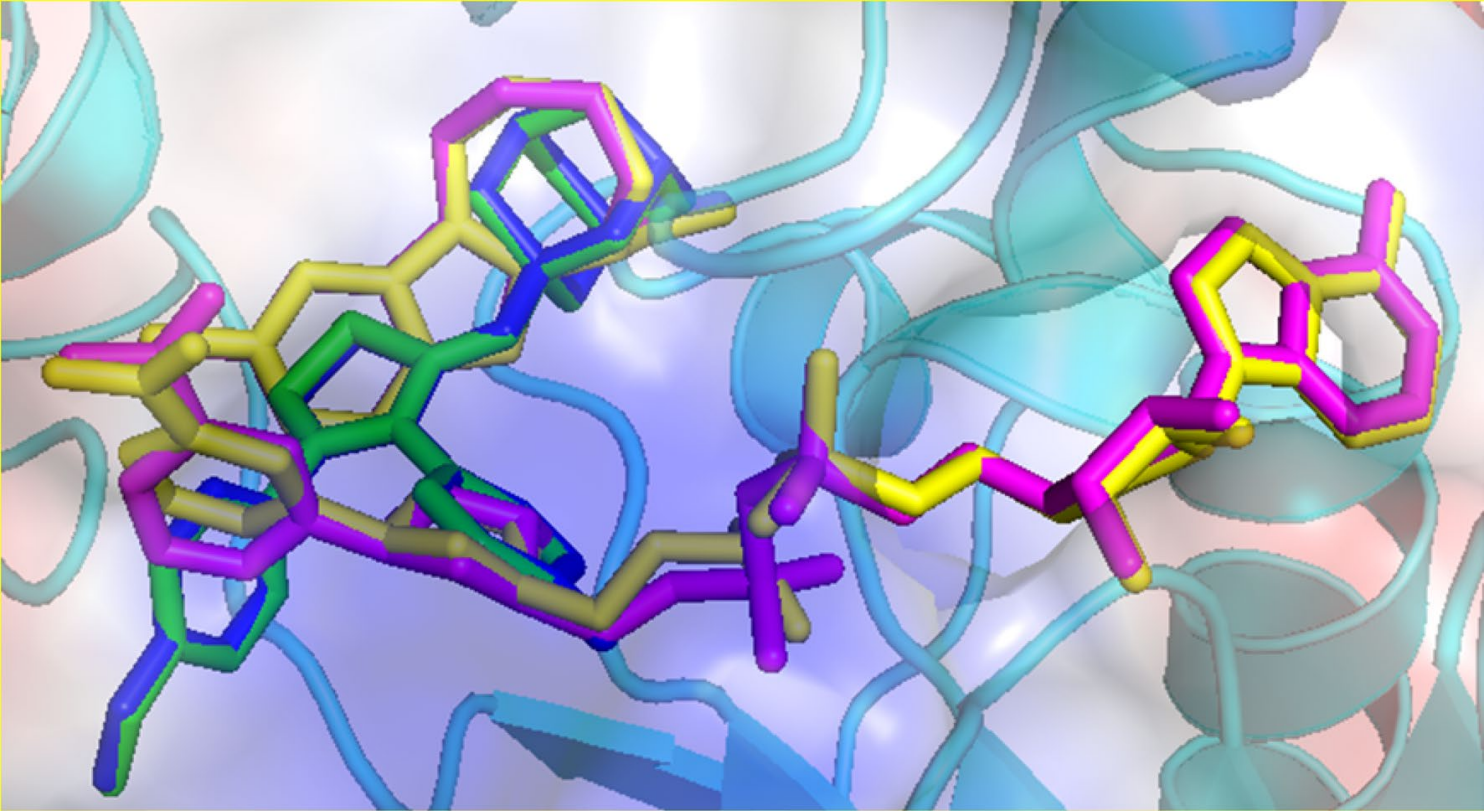
Experimental conformations (yellow) and predicted poses (magenta) of NAD cofactor and EX527 inhibitor are in stick representation at the active site of SIRT1. The top ranked predicted pose of **5e** (green) and **5k** (blue) at the active site is also shown.

As shown in Table 4, compounds **5e** and **5k** showed excellent anti-proliferative activity against all the tested cell lines. In order to understand the bioactivity of these compounds, we carried out molecular docking against the SIRT1 enzyme. Before docking of **5e** and **5k** with SIRT1, we carried out a positive control docking simulation for the NAD cofactor and EX527 inhibitor^63^ (PDB ID: 4I5) with SIRT1. The result showed that there is an excellent agreement between experimental conformations and the predicted poses of NAD cofactor and EX527 (Fig. 7). The binding affinity for the NAD ligand was calculated to be − 13.2 kcal mol^−1^ and − 11.0 kcal mol^−1^ for EX527. The same docking parameters were used for compounds **5e** and **5k** and the results suggest that both compounds showed similar binding affinities (**5e**: − 7.0 and **5k**: − 7.3 kcal mol^−1^).

As can be seen from Fig. 7, compounds **5e** and **5k** bound in a similar manner at the active site of the SIRT1. Moreover, the adamantane cage of these compounds occupies the position of the cycloheptanecarboxamide moiety of the nanomolar inhibitor EX527^64^. The five-membered ring overlaps with the position of the 5-chlorophenyl of EX527. The methoxyphenyl and the substituted phenyl moieties of **5e** and **5k** are closer to the position of the nicotinamide and pentose sugar moieties of the NAD cofactor. The predicted pose of **5e** and **5k** clearly suggest that they could act as potential inhibitors.

**Figure 8:**
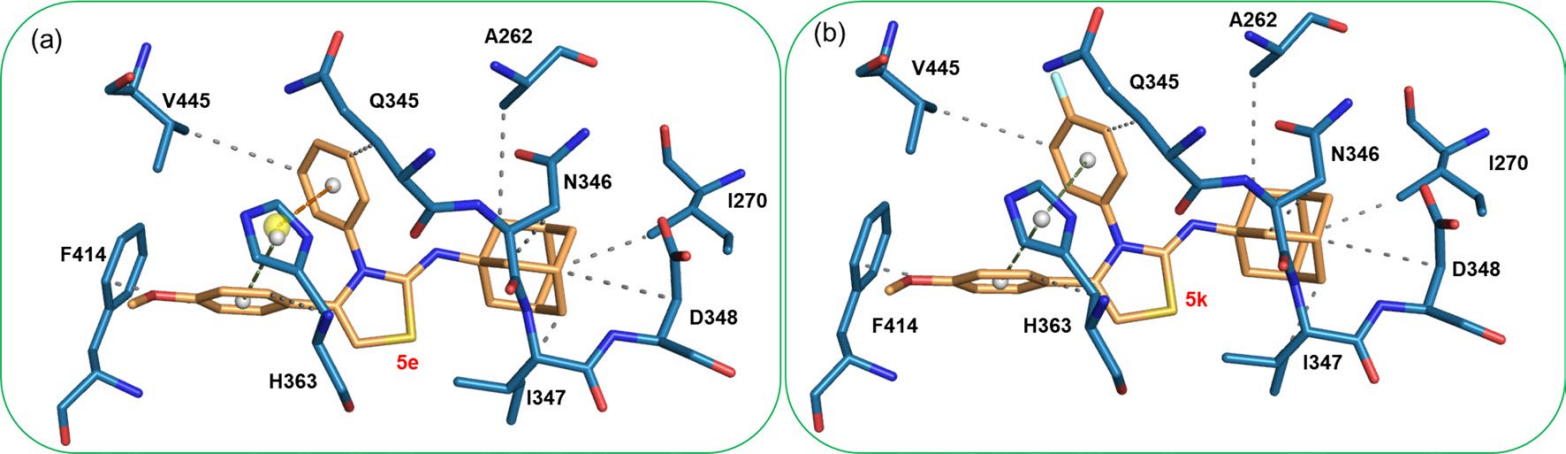
Interactions between SIRT1 (**a**) compound **5e** and (**b**) compound **5k**. Small spheres represent the centroid of the respective rings.

As shown in Fig. 8, the interacting residues of the active site of SIRT1 are identical for both compounds **5e** and **5k** because the binding poses of **5e** and **5k** are similar. The adamantane moiety establishes hydrophobic interactions with Ala 262, Ile 270, Asn 346, Ile 347 and Asp 348. The substituted phenyl also participated in the hydrophobic interaction with Gln 345 and Val 445. It is interesting to note that His 363 establishes π-stacking interaction with the substituted phenyl ring and the methoxy phenyl ring in compounds **5e** and **5k**. The active site residue Phe 414 has participated in a hydrophobic interaction with the methoxy phenyl ring.

## Conclusions

Eighteen new adamantane-linked thiazole derivatives were synthesized and their structures were confirmed by ^1^H NMR, ^13^C NMR data in addition to single crystal X-ray crystallography. The in vitro antibacterial, antifungal activities of the synthesized compounds were assessed against a panel of pathogenic Gram-positive, Gram-negative bacteria and fungi. Compounds **5c**, **5g**, **5l**, **5m**, and **5q** displayed potent broad-spectrum antibacterial activity and compounds **5a** and **5o** showed potent activity against the tested Gram-positive bacteria, while compounds **5b**, **5l** and **5q** displayed potent antifungal activity against *Candida albicans*. The anti-proliferative activity of the synthesized compounds was evaluated towards five human tumor cell lines. Compounds **5e** and **5k** showed potent inhibitory activity against all the tested cell lines. Molecular docking simulation analysis demonstrated that the potent compounds **5e** and **5k** bind to the active site of the SIRT1 enzyme, occupying the positions of the NAD cofactor and the known histone deacetylase inhibitor EX527. The adamantane moiety of these compounds establishes extensive hydrophobic interactions with the active site residues in addition to other interactions.

## Materials and methods

Melting points (℃, uncorrected) were measured in open glass capillaries using Stuart SMP30 electro-thermal melting point apparatus. NMR spectra were obtained on a Bruker 400 Avance III at 400.20 MHz for ^1^H and 100.63 MHz for ^13^C; and Jeol ECA 500 III at 500.16 MHz for ^1^H and 125.77 MHz for ^13^C using CDCl_3_ as solvent. The chemical shifts are expressed in *δ* (ppm) downfield from tetramethylsilane (TMS) as internal standard; coupling constants *(J*) are pressed in Hz. The splitting patterns were designated as: s (singlet), d (doublet), t (triplet), q (quartet) and m (multiplet). Monitoring the reactions and checking the purity of the final products were carried out by thin layer chromatography (TLC) using silica gel pre-coated aluminum sheets (60 F_254_, Merck), petroleum ether/ethyl acetate (8:2) as eluent and visualization with ultraviolet light (UV) at 365 and 254 nm. Compounds **3a-c** were prepared via condensation of adamantan-1-amine **1** with the corresponding aryl isothiocyanate according to the reported procedures^39–41^. Elemental analyses (C, H, N & S) were in agreement with the proposed structures within ± 0.3% of the theoretical values (Table S3). The standard experimental methods for evaluation of the antimicrobial and anti-proliferative activities^42–45,47,50^ are presented in the supplementary data. The reference drugs Ampicillin trihydrate (CAS 7177-48-2), Ciprofloxacin (CAS: 85721-33-1), Fluconazole (CAS: 86386-73-4), Erythromycin (CAS: 114-07-8) and Doxorubicin (CAS 23214-92-8) were purchased from Sigma-Aldrich Chemie GmbH, Germany.

### General procedure for the synthesis of (*Z*)-*N*-(adamantan-1-yl)-3,4-diarylthiazol-2(3*H*)-imines (5a-r)

A mixture of the appropriate 1-(adamantan-1-yl)-3-arylthiourea **3a-c** (2.0 mmol) and the appropriate aryl bromomethyl ketone **4a-f** (2.0 mmol), in ethanol (10 mL), was heated under reflux for 24 h. On cooling, sodium acetate (1.0 g) and crushed ice (100 g) were added and the mixture was stirred for 30 min. and allowed to stand at room temperature for 3 h. The precipitated crude product was filtered, washed with water, dried and crystallized from EtOH/CHCl_3_ or EtOH to yield the target compounds (**5a-r)** as fine colorless crystals.

*(Z)-N-(Adamantan-1-yl)-3,4-diphenylthiazol-2(3H)-imine* **5a**. Yield: 78%, m.p.: 198–200 °C (EtOH/CHCl_3_), Mol. Formula (Mol. Wt.): C_25_H_26_N_2_S (386.56). ^1^H NMR (500.16 MHz): *δ* 1.68 (s, 6H, Adamantane-H), 1.89 (s, 6H, Adamantane-H), 2.09 (s, 3H, Adamantane-H), 5.88 (s, 1H, Thiazole-H), 7.05–7.07 (m, 2H, Ar–H), 7.11–7.23 (m, 8H, Ar–H). ^13^C NMR (125.77 MHz): *δ* 29.64, 36.60, 40.63, 53.49 (Adamantane-C), 96.97 (Thiazole C-5), 126.02, 127.60, 127.77, 127.90, 129.05, 132.52, 138.84, 139.08 (Ar–C), 148.98 (Thiazole C-4), 160.02 (Thiazole C-2).

*(Z)-N-(Adamantan-1-yl)-4-(4-chlorophenyl)-3-phenylthiazol-2(3H)-imine* **5b**. Yield: 62%, m.p.: 176–178 °C (EtOH/CHCl_3_), Mol. Formula (Mol. Wt.): C_25_H_25_ClN_2_S (421.0). ^1^H NMR (400.20 MHz): *δ* 1.68 (s, 6H, Adamantane-H), 1.88 (s, 6H, Adamantane-H), 2.08 (s, 3H, Adamantane-H), 5.89 (s, 1H, Thiazole-H), 6.98 (d, 2H, Ar–H, *J* = 8.4 Hz), 7.10–7.18 (m, 5H, Ar–H), 7.22–7.28 (m, 2H, Ar–H). ^13^C NMR (100.63 MHz): *δ* 29.83, 36.77, 40.82, 53.90 (Adamantane-C), 98.14 (Thiazole C-5), 123.99, 124.59, 126.65, 128.28, 128.45, 129.21, 133.82, 137.98 (Ar–C), 149.30 (Thiazole C-4), 159.34 (Thiazole C-2).

*(Z)-N-(Adamantan-1-yl)-4-(4-bromophenyl)-3-phenylthiazol-2(3H)-imine* **5c**. Yield: 55%, m.p.: 179–181 °C (EtOH/CHCl_3_), Mol. Formula (Mol. Wt.): C_25_H_25_BrN_2_S (465.45). ^1^H NMR (400.20 MHz): *δ* 1.68 (s, 6H, Adamantane-H), 1.88 (s, 6H, Adamantane-H), 2.08 (s, 3H, Adamantane-H), 5.89 (s, 1H, Thiazole-H), 6.93 (d, 2H, Ar–H, *J* = 8.4 Hz), 7.10–7.18 (m, 3H, Ar–H), 7.22–7.30 (m, 4H, Ar–H). ^13^C NMR (100.63 MHz): *δ* 29.78, 36.65, 40.75, 54.19 (Adamantane-C), 99.09 (Thiazole C-5), 120.69, 122.28, 127.12, 128.57, 129.12, 129.58, 131.44, 138.22 (Ar–C), 148.90 (Thiazole C-4), 159.97 (Thiazole C-2).

*(Z)-N-(Adamantan-1-yl)-3-phenyl-4-(p-tolyl)thiazol-2(3H)-imine* **5d**. Yield: 88%, m.p.: 158–160 °C (EtOH), Mol. Formula (Mol. Wt.): C_26_H_28_N_2_S (400.58). ^1^H NMR (400.20 MHz): *δ* 1.70 (s, 6H, Adamantane-H), 1.92 (s, 6H, Adamantane-H), 2.11 (s, 3H, Adamantane-H), 2.28 (s, 3H, CH_3_), 5.85 (s, 1H, Thiazole-H), 6.97–6.99 (m, 4H, Ar–H), 7.14–7.17 (m, 3H, Ar–H), 7.22–7.28 (m, 2H, Ar–H). ^13^C NMR (100.63 MHz): *δ* 21.29 (CH_3_), 29.89, 36.83, 40.86, 53.78 (Adamantane-C), 96.56 (Thiazole C-5), 123.90, 126.27, 127.98, 128.89, 129.36, 130.0, 137.78, 139.19 (Ar–C), 149.42 (Thiazole C-4), 160.08 (Thiazole C-2).

*(Z)-N-(Adamantan-1-yl)-4-(4-methoxyphenyl)-3-phenylthiazol-2(3H)-imine* **5e**. Yield: 85%, m.p.: 156–158 °C (EtOH/CHCl_3_), Mol. Formula (Mol. Wt.): C_26_H_28_N_2_OS (416.58). ^1^H NMR (400.20 MHz): *δ* 1.67 (s, 6H, Adamantane-H), 1.89 (s, 6H, Adamantane-H), 2.08 (s, 3H, Adamantane-H), 3.73 (s, 3H, OCH_3_), 5.78 (s, 1H, Thiazole-H), 6.67 (d, 2H, Ar–H, *J* = 8.8 Hz), 6.97 (d, 2H, Ar–H, *J* = 8.8 Hz), 7.11–7.15 (m, 3H, Ar–H), 7.20–7.26 (m, 2H, Ar–H). ^13^C NMR (100.63 MHz): *δ* 29.87, 36.82, 40.86, 53.80 (Adamantane-C), 55.22 (OCH_3_), 96.13 (Thiazole C-5), 113.56, 125.12, 126.38, 128.10, 129.39, 138.88, 139.25, 157.71 (Ar–C), 149.77 (Thiazole C-4), 159.23 (Thiazole C-2).

*(Z)-N-(Adamantan-1-yl)-4-(naphthalen-2-yl)-3-phenylthiazol-2(3H)-imine* **5f**. Yield: 80%, m.p.: 177–179 °C (EtOH/CHCl_3_), Mol. Formula (Mol. Wt.): C_29_H_28_N_2_S (436.62). ^1^H NMR (400.20 MHz): *δ* 1.76 (s, 6H, Adamantane-H), 1.99 (s, 6H, Adamantane-H), 2.17 (s, 3H, Adamantane-H), 6.04 (s, 1H, Thiazole-H), 7.08–7.26 (m, 6H, Ar–H), 7.47- 7.50 (m, 2H, Ar–H), 7.59 (d, 1H, Ar–H, *J* = 8.4 Hz), 7.74- 7.78 (m, 3H, Ar–H). ^13^C NMR (100.63 MHz): *δ* 29.85, 36.78, 40.84, 53.94 (Adamantane-C), 98.21 (Thiazole C-5), 125.55, 126.38, 126.42, 126.51, 127.15, 127.56, 127.64, 128.08, 128.19, 129.21, 132.66, 133.02, 139.03, 139.18 (Ar–C), 150.02 (Thiazole C-4), 159.02 (Thiazole C-2).

*(Z)-N-(Adamantan-1-yl)-3-(4-fluorophenyl)-4-phenylthiazol-2(3H)-imine* **5g**. Yield: 90%, m.p.: 193–195 °C (EtOH/CHCl_3_), Mol. Formula (Mol. Wt.): C_25_H_25_FN_2_S (404.55). ^1^H NMR (500.16 MHz): *δ* 1.65 (s, 6H, Adamantane-H), 1.85 (s, 6H, Adamantane-H), 2.06 (s, 3H, Adamantane-H), 5.85 (s, 1H, Thiazole-H), 6.86–6.89 (m, 2H, Ar–H), 7.03–7.08 (m, 4H, Ar–H), 7.15–7.17 (m, 3H, Ar–H). ^13^C NMR (125.77 MHz): *δ* 29.88, 36.84, 40.93, 53.77 (Adamantane-C), 97.24 (Thiazole C-5), 114.86, 128.02, 128.29, 130.76, 132.54, 135.25, 138.90, 161.73 (Ar–C), 149.27 (Thiazole C-4), 159.78 (Thiazole C-2).

*(Z)-N-(Adamantan-1-yl)-4-(4-chlorophenyl)-3-(4-fluorophenyl)thiazol-2(3H)-imine* **5h**. Yield: 92%, m.p.: 194–196 °C (EtOH/CHCl_3_), Mol. Formula (Mol. Wt.): C_25_H_24_ClFN_2_S (438.99). ^1^H NMR (500.16 MHz): *δ* 1.66 (s, 6H, Adamantane-H), 1.85 (s, 6H, Adamantane-H), 2.07 (s, 3H, Adamantane-H), 5.87 (s, 1H, Thiazole-H), 6.89–6.93 (m, 2H, Ar–H), 6.96–6.99 (m, 2H, Ar–H), 7.04–7.07 (m, 2H, Ar–H), 7.14–7.16 (m, 2H, Ar–H). ^13^C NMR (125.77 MHz): *δ* 29.84, 36.76, 40.88, 53.99 (Adamantane-C), 98.20 (Thiazole C-5), 115.19, 128.62, 128.80, 129.11, 130.74, 134.09, 137.87, 161.95 (Ar–C), 149.76 (Thiazole C-4), 159.99 (Thiazole C-2).

*(Z)-N-(Adamantan-1-yl)-4-(4-bromophenyl)-3-(4-fluorophenyl)thiazol-2(3H)-imine* **5i**. Yield: 84%, m.p.: 221–223 °C (EtOH/CHCl_3_), Mol. Formula (Mol. Wt.): C_25_H_24_BrFN_2_S (483.44). ^1^H NMR (400.20 MHz): *δ* 1.66 (s, 6H, Adamantane-H), 1.85 (s, 6H, Adamantane-H), 2.07 (s, 3H, Adamantane-H), 5.88 (s, 1H, Thiazole-H), 6.89–6.95 (m, 4H, Ar–H), 7.04–7.08 (m, 2H, Ar–H), 7.29 (d, 2H, Ar–H, *J* = 8.4 Hz). ^13^C NMR (100.63 MHz): *δ* 29.85, 36.81, 40.93, 53.80 (Adamantane-C), 97.90 (Thiazole C-5), 115.03, 122.14, 129.46, 130.71, 131.44, 135.04, 137.73, 162.04 (Ar–C), 148.72 (Thiazole C-4), 159.60 (Thiazole C-2).

*(Z)-N-(Adamantan-1-yl)-3-(4-fluorophenyl)-4-(p-tolyl)thiazol-2(3H)-imine* **5j**. Yield: 64%, m.p.: 144–146 °C (EtOH), Mol. Formula (Mol. Wt.): C_26_H_27_FN_2_S (418.57). ^1^H NMR (400.20 MHz): *δ* 1.69 (s, 6H, Adamantane-H), 1.89 (s, 6H, Adamantane-H), 2.10 (s, 3H, Adamantane-H), 2.29 (s, 3H, CH_3_), 5.84 (s, 1H, Thiazole-H), 6.90–7.01 (m, 6H, Ar–H), 7.09–7.12 (m, 2H, Ar–H). ^13^C NMR (100.63 MHz): *δ* 21.23 (CH_3_), 29.82, 36.76, 40.85, 53.78 (Adamantane-C), 96.68 (Thiazole C-5), 114.84, 127.95, 128.94, 130.77, 135.16, 137.94, 138.95, 161.96 (Ar–C), 149.56 (Thiazole C-4), 159.52 (Thiazole C-2).

*(Z)-N-(Adamantan-1-yl)-3-(4-fluorophenyl)-4-(4-methoxyphenyl)thiazol-2(3H)-imine* **5k**. Yield: 94%, m.p.: 179–181 °C (EtOH/CHCl_3_), Mol. Formula (Mol. Wt.): C_26_H_27_FN_2_OS (434.57). ^1^H NMR (400.20 MHz): *δ* 1.68 (s, 6H, Adamantane-H), 1.87 (s, 6H, Adamantane-H), 2.08 (s, 3H, Adamantane-H), 3.74 (s, 3H, OCH_3_), 5.78 (s, 1H, Thiazole-H), 6.69 (d, 2H, Ar–H, *J* = 8.8 Hz), 6.88–6.98 (m, 4H, Ar–H), 7.06–7.10 (m, 2H, Ar–H). ^13^C NMR (100.63 MHz): *δ* 29.86, 36.80, 40.90, 53.80 (Adamantane-C, 55.24 (OCH_3_), 96.11 (Thiazole C-5), 113.67, 114.88, 124.85, 129.44, 130.85, 135.15, 138.68, 161.99 (Ar–C), 149.76 (Thiazole C-4), 159.55 (Thiazole C-2).

*(Z)-N-(Adamantan-1-yl)-3-(4-fluorophenyl)-4-(naphthalen-2-yl)thiazol-2(3H)-imine* **5l**. Yield: 60%, m.p.: 155–157 °C (EtOH/CHCl_3_), Mol. Formula (Mol. Wt.): C_29_H_27_FN_2_S (454.61). ^1^H NMR (500.16 MHz): *δ* 1.68–1.69 (m, 6H, Adamantane-H), 1.89–1.00 (m, 6H, Adamantane-H), 2.09 (s, 3H, Adamantane-H), 5.98 (s, 1H, Thiazole-H), 6.85–6.89 (m, 2H, Ar–H), 7.01–7.02 (m, 1H, Ar–H), 7.11–7.15 (m, 2H, Ar–H), 7.43–7.46 (m, 2H, Ar–H), 7.57 (d, 1H, Ar–H, *J* = 8.**5h**z), 7.68 (s, 1H, Ar–H), 7.71–7.75 (m, 2H, Ar–H). ^13^C NMR (125.77 MHz): *δ* 29.86, 36.78, 40.85, 54.04 (Adamantane-C), 98.18 (Thiazole C-5), 115.10, 125.58, 126.58, 127.37, 127.74, 128.14, 129.79, 130.77, 130.84, 132.77, 133.06, 134.91, 139.13, 161.88 (Ar–C), 150.28 (Thiazole C-4), 159.93 (Thiazole C-2).

*(Z)-N-(Adamantan-1-yl)-3-(4-chlorophenyl)-4-phenylthiazol-2(3H)-imine* **5m**. Yield: 76%, m.p.: 190–192 °C (EtOH/CHCl_3_), Mol. Formula (Mol. Wt.): C_25_H_25_ClN_2_S (421.0). ^1^H NMR (500.16 MHz): *δ* 1.66–1.68 (m, 6H, Adamantane-H), 1.86–1.87 (m, 6H, Adamantane-H), 2.08 (s, 3H, Adamantane-H), 5.87 (s, 1H, Thiazole-H), 7.04–7.07 (m, 4H, Ar–H), 7.15–7.20 (m, 5H, Ar–H). ^13^C NMR (125.77 MHz): *δ* 30.12, 37.08, 41.17, 54.08 (Adamantane-C), 97.97 (Thiazole C-5), 128.25, 128.34, 128.44, 128.64, 130.67, 132.74, 138.09, 138.87 (Ar–C), 149.10 (Thiazole C-4), 159.09 (Thiazole C-2).

*(Z)-N-(Adamantan-1-yl)-3,4-bis(4-chlorophenyl)thiazol-2(3H)-imine* **5n**. Yield: 77%, m.p.: 197–199 °C (EtOH/CHCl_3_), Mol. Formula (Mol. Wt.): C_25_H_24_Cl_2_N_2_S (455.44). ^1^H NMR (400.20 MHz): *δ* 1.66 (s, 6H, Adamantane-H), 1.85 (s, 6H, Adamantane-H), 2.06 (s, 3H, Adamantane-H), 5.88 (s, 1H, Thiazole-H), 6.97 (d, 2H, Ar–H, *J* = 8.4 Hz), 7.03 (d, 2H, Ar–H, *J* = 8.8 Hz), 7.15–7.20 (m, 4H, Ar–H). ^13^C NMR (100.63 MHz): *δ* 29.81, 36.74, 40.86, 53.99 (Adamantane-C), 98.48 (Thiazole C-5), 128.45, 128.68, 129.18, 130.34, 134.08, 137.54 (Ar–C), 148.55 (Thiazole C-4), 159.02 (Thiazole C-2).

*(Z)-N-(Adamantan-1-yl)-4-(4-bromophenyl)-3-(4-chlorophenyl)thiazol-2(3H)-imine* **5o**. Yield: 82%, m.p.: 210–212 °C (EtOH/CHCl_3_), Mol. Formula (Mol. Wt.): C_25_H_24_BrClN_2_S (499.90). ^1^H NMR (400.20 MHz): *δ* 1.66 (s, 6H, Adamantane-H), 1.84 (s, 6H, Adamantane-H), 2.07 (s, 3H, Adamantane-H), 5.89 (s, 1H, Thiazole-H), 6.91 (d, 2H, Ar–H, *J* = 8.0 Hz), 7.03 (d, 2H, Ar–H, *J* = 8.4 Hz), 7.18 (d, 2H, Ar–H, *J* = 8.4 Hz), 7.30 (d, 2H, Ar–H, *J* = 8.0 Hz). ^13^C NMR (100.63 MHz): *δ* 29.81, 36.75, 40.88, 53.93 (Adamantane-C), 98.46 (Thiazole C-5), 122.24, 128.42, 129.40, 130.33, 131.61, 137.53 (Ar–C), 148.47 (Thiazole C-4), 159.07 (Thiazole C-2).

*(Z)-N-(Adamantan-1-yl)-3-(4-chlorophenyl)-4-(p-tolyl)thiazol-2(3H)-imine* **5p**. Yield: 90%, m.p.: 188–190 °C (EtOH), Mol. Formula (Mol. Wt.): C_26_H_27_ClN_2_S (435.03). ^1^H NMR (500.16 MHz): *δ* 1.65–1.67 (m, 6H, Adamantane-H), 1.85–1.86 (m, 6H, Adamantane-H), 2.07 (s, 3H, Adamantane-H), 2.28 (s, 3H, CH_3_), 5.82 (s, 1H, Thiazole-H), 6.92 (d, 2H, Ar–H, *J* = 8.0 Hz), 6.98 (d, 2H, Ar–H, *J* = 8.0 Hz), 7.04–7.06 (m, 2H, Ar–H), 7.15–7.17 (m, 2H, Ar–H). ^13^C NMR (125.77 MHz): *δ* 21.33 (CH_3_), 29.87, 36.83, 40.91, 53.80 (Adamantane-C), 97.03 (Thiazole C-5), 127.90, 128.18, 129.09, 129.50, 130.47, 131.25, 138.02, 138.69 (Ar–C), 149.0 (Thiazole C-4), 158.89 (Thiazole C-2).

*(Z)-N-(Adamantan-1-yl)-3-(4-chlorophenyl)-4-(4-methoxyphenyl)thiazol-2(3H)-imine* **5q**. Yield: 82%, m.p.: 160–162 °C (EtOH/CHCl_3_), Mol. Formula (Mol. Wt.): C_26_H_27_ClN_2_OS (451.03). ^1^H NMR (500.16 MHz): *δ* 1.65–1.67 (m, 6H, Adamantane-H), 1.85–1.86 (m, 6H, Adamantane-H), 2.07 (s, 3H, Adamantane-H), 3.75 (s, 3H, OCH_3_), 5.78 (s, 1H, Thiazole-H), 6.70 (d, 2H, Ar–H, *J* = 9.0 Hz), 6.95 (d, 2H, Ar–H, *J* = 9.0 Hz), 7.04 (d, 2H, Ar–H, *J* = 9.0 Hz), 7.16 (d, 2H, Ar–H, *J* = 8.**5h**z). ^13^C NMR (125.77 MHz): *δ* 29.85, 36.80, 40.86, 53.84 (Adamantane-C), 55.30 (OCH_3_), 96.47 (Thiazole C-5), 113.76, 124.84, 128.23, 129.39, 130.53, 131.71, 137.78, 138.44 (Ar–C), 149.43 (Thiazole C-4), 159.36 (Thiazole C-2).

*(Z)-N-(Adamantan-1-yl)-3-(4-chlorophenyl)-4-(naphthalen-2-yl)thiazol-2(3H)-imine* **5r**. Yield: 89%, m.p.: 177–179 °C (EtOH/CHCl_3_), Mol. Formula (Mol. Wt.): C_29_H_27_ClN_2_S (471.06). ^1^H NMR (500.16 MHz): *δ* 1.68 (s, 6H, Adamantane-H), 1.88–1.89 (m, 6H, Adamantane-H), 2.09 (s, 3H, Adamantane-H), 5.99 (s, 1H, Thiazole-H), 6.99–7.02 (m, 1H, Ar–H), 7.10–7.15 (m, 4H, Ar–H), 7.44–7.48 (m, 2H, Ar–H), 7.58 (d, 1H, Ar–H, *J* = 9.0 Hz), 7.69 (s, 1H, Ar–H), 7.71–7.75 (m, 2H, Ar–H). ^13^C NMR (125.77 MHz): *δ* 29.87, 36.83, 40.93, 53.90 (Adamantane-C), 98.34 (Thiazole C-5), 125.49, 126.59, 127.11, 127.76, 127.89, 128.13, 128.28, 130.05, 130.37, 131.72, 132.77, 133.12, 137.79, 138.71 (Ar–C), 148.94 (Thiazole C-4), 158.96 (Thiazole C-2).

### Preparation of the single crystal of compounds 5d and 5f

Analytically-pure samples of compounds **5d** and **5f.** (50 mg) were dissolved in 1**5m**L cyclohexane/CHCl_3_ and EtOH/CHCl_3_ (1:1, *v/v*), respectively. Slow evaporation of the solutions at room temperature yielded the single crystals as colorless prisms.

### Molecular Modelling and molecular docking analysis

The structures of **5e** and **5k** were drawn using ChemSketch Freeware (www.acdlabs.com) and these structures were subsequently optimized with the B3LYP/6-31G level of theory using the Gaussian 09 program^65^. The optimized structures were used for molecular docking simulations. The crystal structure of SIRT1 catalytic domain bound to NAD and an EX527 analog was retrieved from the protein data bank (PDB ID: 4I5I). The molecular docking simulation against SIRT1 was performed using the AutoDock Vina program^66^ available in PyRX-0.8 virtual tools^67^. The chain A of 4I5I and the ligands **5e** and **5k** were prepared and used for docking calculation. The active site grid was constructed based on the position of NAD ligand. To check the predictive power of the AutoDock Vina program, we also performed docking for the cocrystallized ligand NAD. The experimental and predicted poses were very similar. The top ranked binding poses were considered for protein–ligand interaction analysis using PLIP web server^68^.

## Supplementary Information

Supplementary Information.

## Data Availability

The crystallographic data for the structures of compounds **5d** (CCDC #: 2,155,510) and **5f.** (CCDC #: 2,155,511) could be obtained free of charge from the Cambridge Crystallographic Data Centre (www.ccdc.cam.ac.uk/data_request/cif).
