# Supplementary material for: Synthesis and in vitro antibacterial, antifungal, anti-proliferative activities of novel adamantane-containing thiazole compounds
Source: Sci Rep. 2022 Dec 6;12:21058. doi: 10.1038/s41598-022-25390-0 (PMC9726863; doi:10.1038/s41598-022-25390-0)

Supplementary data

Synthesis and *in vitro* antibacterial, antifungal, anti-proliferative activities of novel adamantane-containing thiazole compounds

Eman T. Warda [1], Mahmoud B. El-Ashmawy [1], El-Sayed E. Habib [2], Mohammed S. M. Abdelbaky [3], Santiago Garcia-Granda [3], Subbiah Thamotharan [4], Ali A. El-Emam [1]*

[1] *Department of Medicinal Chemistry, Faculty of Pharmacy, Mansoura University, Mansoura 35516, Egypt*
[2] *Department of Microbiology and Immunology, Faculty of Pharmacy, Mansoura University, Mansoura 35516, Egypt*
[3] *Department of Physical and Analytical Chemistry, Faculty of Chemistry, Oviedo University-CINN, Oviedo 33006, Spain*
[4] *Biomolecular Crystallography Laboratory, Department of Bioinformatics, School of chemical and Biotechnology, SASTRA Deemed University, Thanjavur-613 401, India*

Contents

1. Crystal data and structure refinement parameters for compounds **5d** and **5f** (Table S1).
2. PASS prediction of compounds **5a-r** (Table S2).
3. Molecular formulae, molecular weights and elemental analyses data of compounds **5a-r** (Table S3)
4. NMR spectra of synthesised compounds **5a-r**.
5. Checkcif reports for crystals **5d** and **5f**.
6. Determination of antimicrobial activity of compounds **5a-r** (agar disc-diffusion method).
7. Determination of minimal inhibitory concentrations (MIC) and the minimal biocidal concentrations (MBC) for compounds **5a**, **5c**, **5g**, **5l**, **5m**, **5o** and **5q** (micro-dilution susceptibility method.
8. Determination of bacterial biofilm inhibitory activity of compounds **5a**, **5c**, **5l** and **5o** (crystal violet staining method).
9. Determination of anti-proliferative activity (MTT assay)

Table S1. Crystal data and structure refinement parameters for compounds **5d** and **5f**.

	**Compound 5d**	**Compound 5f**
Empirical formula	$C_{26}H_{28}N_2S{\cdot}0.5(C_6H_{12})$	$C_{29}H_{28}N_2S$
Formula weight	442.64	436.59
Crystal system	Monoclinic	Triclinic
Space group	I2/a	P-1
Temperature (K)	121 (2)	329 (2)
a/Å	18.7865 (3)	7.1421 (5)
b/Å	10.8565 (2)	11.6290 (8)
c/Å	23.2644 (5)	15.1229 (12)
α/°	90	107.993 (7)
β/°	92.425(2)	99.633 (6)
γ/°	90	95.238 (6)
Volume/$Å^3$	1815.88 (14)	1163.99 (16)
Z	8	2
ρcalcg/cm^3	1.240	1.246
μ/mm^{-1}	1.341	1.365
F(000)	1904	464
Crystal size (mm^3)	0.15 × 0.10 × 0.09	0.30 × 0.06 × 0.03
Radiation	Cu Kα (λ=1.54184)	
Diffractometer	Xcalibur, Ruby, Gemini diffractometer	
Absorption correction	Multi-scan	
T_{min}, T_{max}	0.851, 0.886	0.906, 0.960
2Θ range for data collection/°	3.8-74.6°	3.1-71.6°
Index ranges	h = −23→23, k = −13→13, l = −126→29	h = −8→7, k = −14→14, l = −18→18
Reflections collected	18428	17828
Independent reflections	4793	4698
Data/restraints/parameters	290/0/0	289/0/0
Goodness-of-fit on F^2	1.04	1.01
Final R indexes [I>=2σ (I)]	0.069	0.054
Final R indexes [all data]	0.206	0.148
Largest diff. peak and hole (e $Å^{-3}$)	1.16, -0.59	0.21, -0.19
CCDC No.	2155510	2155511

Table S2. PASS prediction of histone deacetylase SIRT1 inhibitory activity of compounds **5a-r**.

Comp. No.	Pa	Pi	Comp. No.	Pa	Pi
**5a**	0,667	0,002	**5j**	0,488	0,003
**5b**	0,579	0,003	**5k**	0,488	0,003
**5c**	0,666	0,002	**5l**	0,666	0,002
**5d**	0,563	0,003	**5m**	0,579	0,003
**5e**	0,518	0,003	**5n**	0,591	0,002
**5f**	0,649	0,002	**5o**	0,600	0,002
**5g**	0,570	0,003	**5p**	0,496	0,003
**5h**	0,502	0,003	**5q**	0,461	0,003
**5i**	0,593	0,002	**5r**	0,562	0,003

Pa: probability to be active, Pi: probability to be inactive.

Table S3. Molecular formulae, molecular weights and elemental analyses data of compounds **5a-r**.

Comp. No.	Mol. Formula (Mol. Wt.)	Analysis: % Calcd. (Found)			
		C	H	N	S
**5a**	$C_{25}H_{26}N_2S$ (386.56)	77.68 (77.52)	6.78 (6.80)	7.25 (7.22)	8.29 (8.25)
**5b**	$C_{25}H_{25}ClN_2S$ (421.0)	71.32 (71.30)	5.99 (6.02)	6.65 (6.62)	7.62 (7.61)
**5c**	$C_{25}H_{25}BrN_2S$ (465.45)	64.51 (64.46)	5.41 (5.52)	6.02 (5.98)	6.89 (6.88)
**5d**	$C_{26}H_{28}N_2S$ (400.58)	77.96 (77.90)	7.05 (7.12)	6.99 (6.75)	8.0 (7.92)
**5e**	$C_{26}H_{28}N_2OS$ (416.58)	74.96 (74.80)	6.78 (6.80)	6.72 (6.68)	7.70 (7.62)
**5f**	$C_{29}H_{28}N_2S$ (436.62)	79.78 (79.76)	6.46 (6.50)	6.42 (6.40)	7.34 (7.33)
**5g**	$C_{25}H_{25}FN_2S$ (404.55)	74.22 (74.16)	6.23 (6.25)	6.92 (6.90)	7.92 (7.88)
**5h**	$C_{25}H_{24}ClFN_2S$ (438.99)	68.40 (68.23)	5.51 (5.50)	6.38 (6.39)	7.30 (7.28)
**5i**	$C_{25}H_{24}BrFN_2S$ (483.44)	62.11 (62.08)	5.0 (5.12)	5.79 (5.72)	6.63 (6.62)
**5j**	$C_{26}H_{27}FN_2S$ (418.57)	74.61 (74.62)	6.50 (6.55)	6.69 (6.62)	7.66 (7.65)
**5k**	$C_{26}H_{27}FN_2OS$ (434.57)	71.86 (71.82)	6.26 (6.30)	6.45 (6.32)	7.38 (7.38)
**5l**	$C_{29}H_{27}FN_2S$ (454.61)	76.62 (76.50)	5.99 (6.08)	6.16 (6.12)	7.05 (7.0)
**5m**	$C_{25}H_{25}ClN_2S$ (421.0)	71.32 (71.25)	5.99 (6.05)	6.65 (6.65)	7.62 (7.61)
**5n**	$C_{25}H_{24}Cl_2N_2S$ (455.44)	65.93 (65.80)	5.31 (5.33)	6.15 (6.17)	7.04 (6.98)
**5o**	$C_{25}H_{24}BrClN_2S$ (499.90)	60.07 (59.92)	4.84 (4.85)	5.60 (5.58)	6.41 (6.40)
**5p**	$C_{26}H_{27}ClN_2S$ (435.03)	71.79 (71.72)	6.26 (6.32)	6.44 (6.45)	7.37 (7.35)
**5q**	$C_{26}H_{27}ClN_2OS$ (451.03)	69.24 (69.25)	6.03 (6.08)	6.21 (6.18)	7.11 (7.10)
**5r**	$C_{29}H_{27}ClN_2S$ (471.06)	73.94 (73.82)	5.78 (5.80)	5.95 (5.86)	6.81 (6.80)

NMR Spectra

Compound **5a**

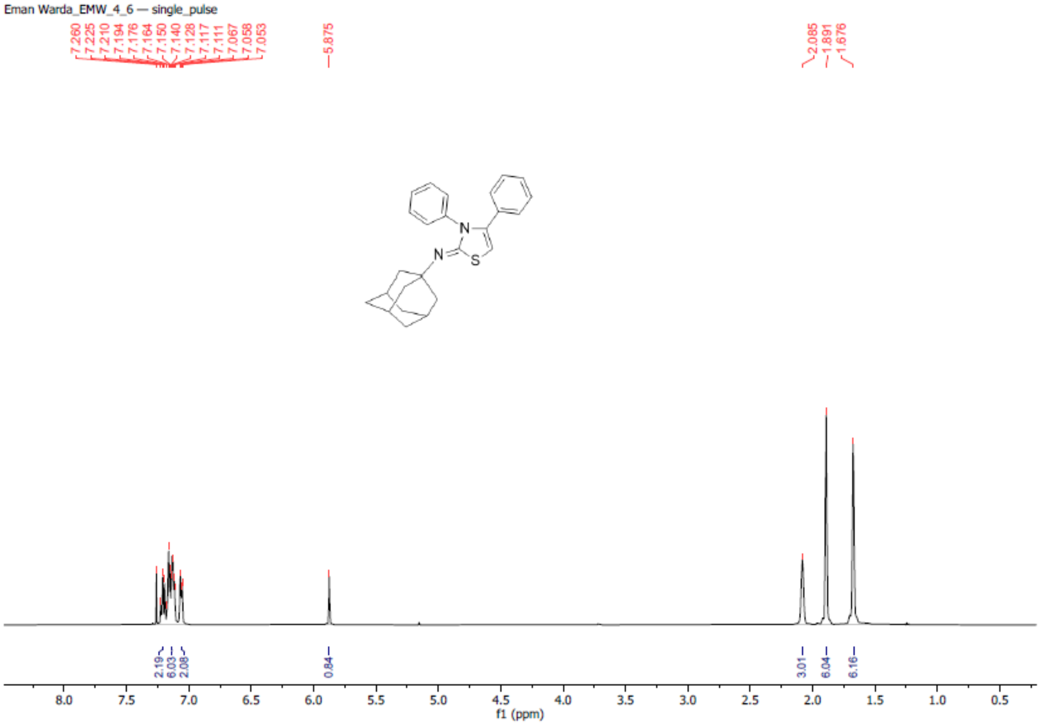

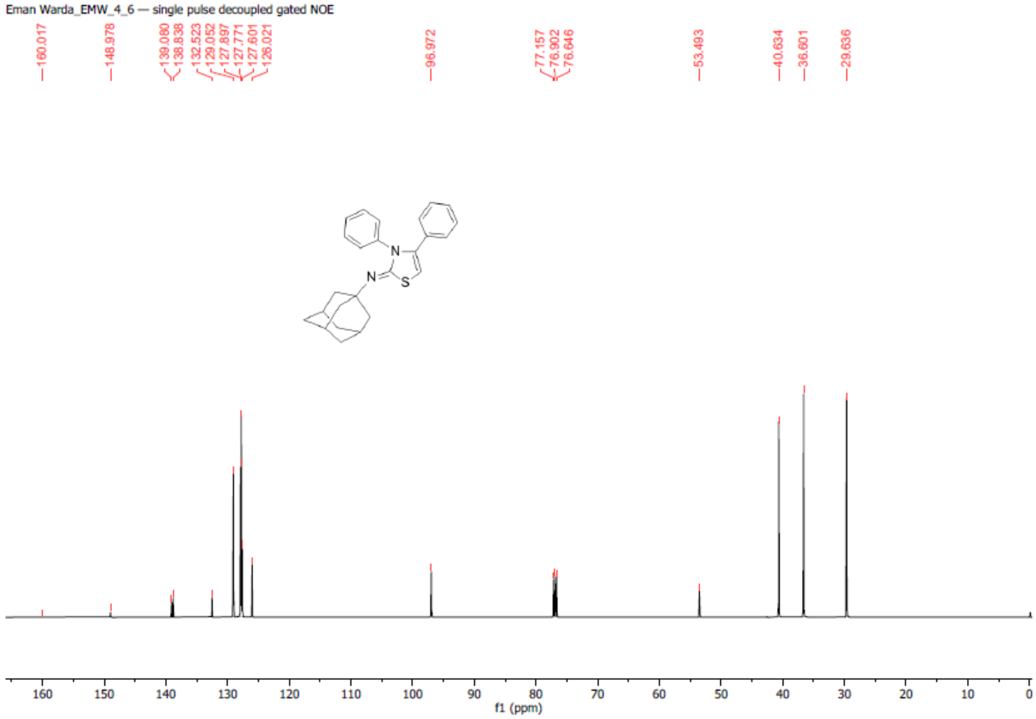

Compound **5b**

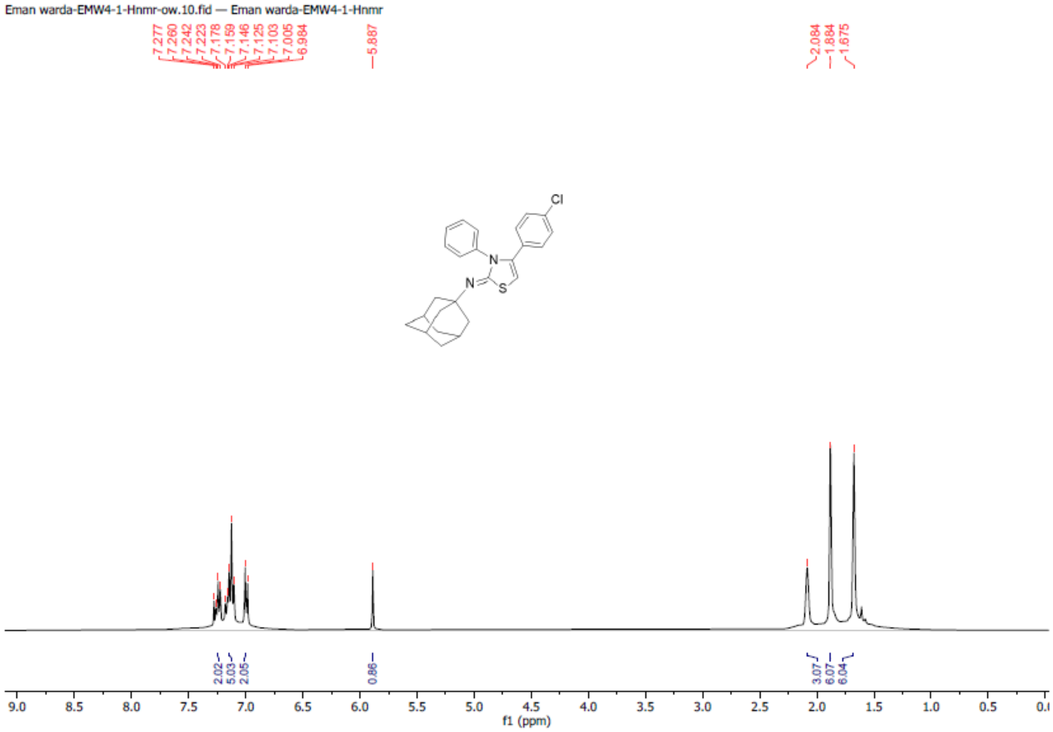
Eman warda-EMW4-1-Hnmr-ow.10.fid — Eman warda-EMW4-1-Hnmr
7.277
7.260
7.242
7.223
7.178
7.159
7.146
7.125
7.103
7.005
6.984
5.887
2.084
1.884
1.675
Cl
N
N
S
2.02
5.03
2.05
0.86
3.07
6.07
6.04
9.0
8.5
8.0
7.5
7.0
6.5
6.0
5.5
5.0
4.5
4.0
3.5
3.0
2.5
2.0
1.5
1.0
0.5
f1 (ppm)

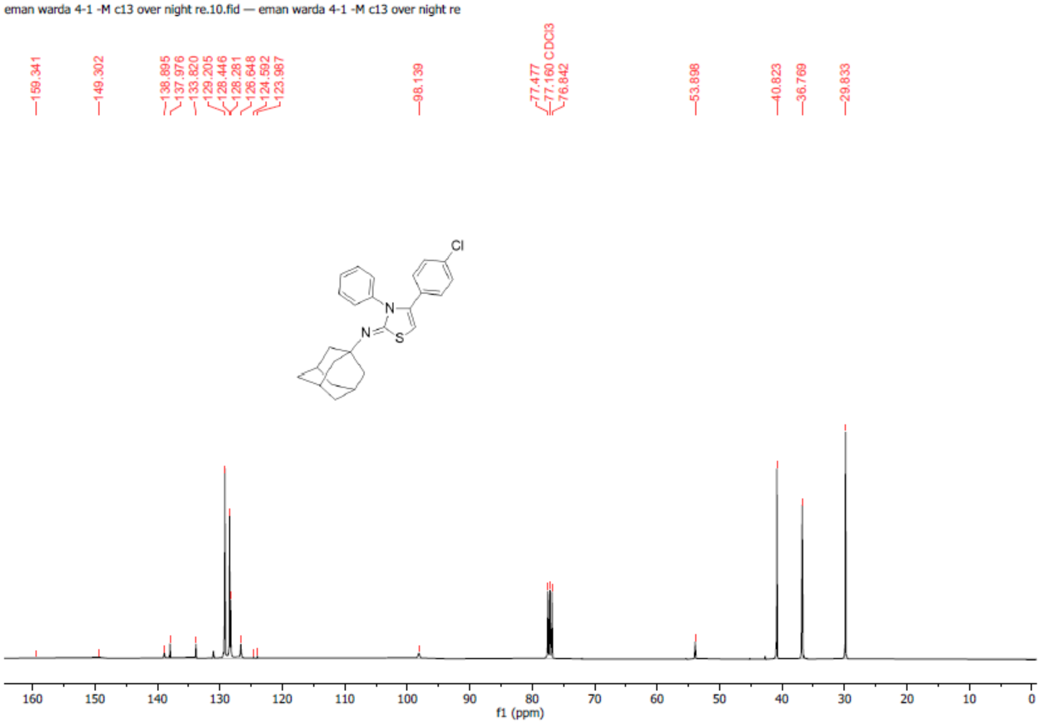
eman warda 4-1 -M c13 over night re.10.fid — eman warda 4-1 -M c13 over night re
159.341
149.302
138.895
137.976
133.820
129.205
128.446
128.281
126.648
124.592
123.987
98.139
77.477
77.160 CDCl3
76.842
53.898
40.823
36.769
29.833
Cl
N
N
S
160
150
140
130
120
110
100
90
80
70
60
50
40
30
20
10
0
f1 (ppm)

Compound **5c**

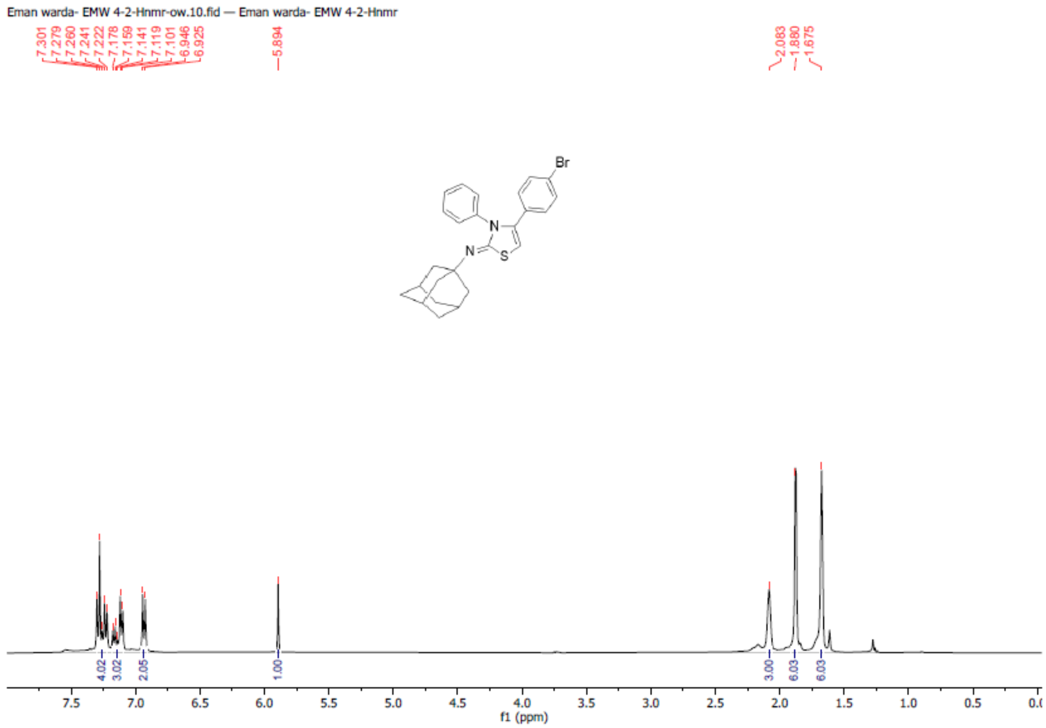

Eman warda- EMW 4-2-Hnmr-ow.10.fid — Eman warda- EMW 4-2-Hnmr
7.301 7.279 7.260 7.241 7.222 7.178 7.159 7.141 7.119 7.101 6.946 6.925
5.894
2.083 1.880 1.675
Br
N
N
S
4.02 3.02 2.05
1.00
3.00 6.03 6.03
7.5 7.0 6.5 6.0 5.5 5.0 4.5 4.0 3.5 3.0 2.5 2.0 1.5 1.0 0.5
f1 (ppm)

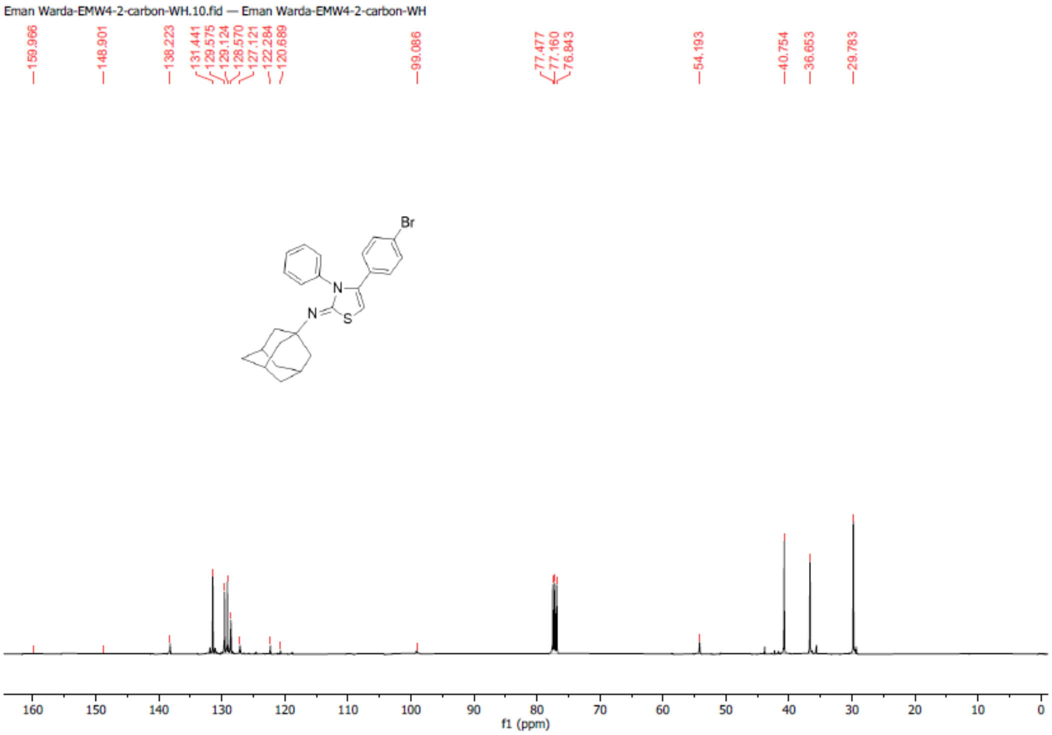

Eman Warda-EMW4-2-carbon-WH.10.fid — Eman Warda-EMW4-2-carbon-WH
159.966
148.901
138.223
131.441 129.575 129.124 128.570 127.121 122.284 120.689
99.086
77.477 77.160 76.843
54.193
40.754
36.653
29.783
Br
N
N
S
160 150 140 130 120 110 100 90 80 70 60 50 40 30 20 10 0
f1 (ppm)

Compound **5d**

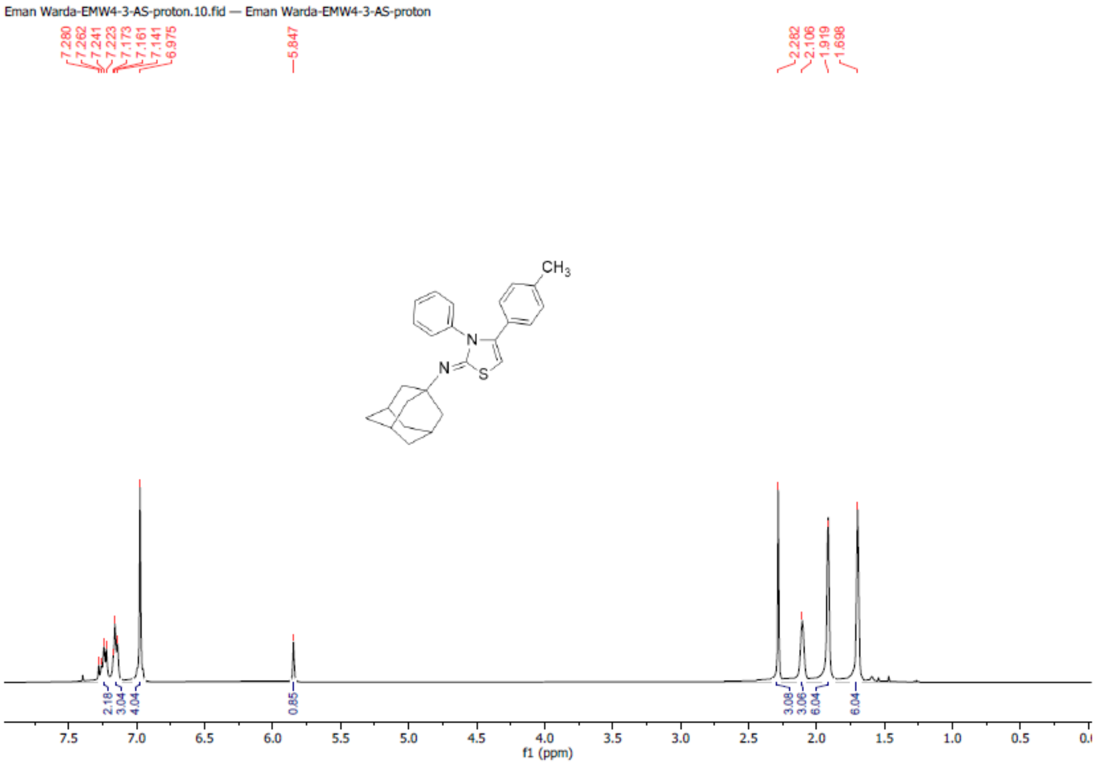
Eman Warda-EMW4-3-AS-proton.10.fid — Eman Warda-EMW4-3-AS-proton
7.280
7.262
7.241
7.223
7.173
7.161
7.141
6.975
5.847
2.282
2.106
1.919
1.698
CH3
N
N
S
2.18
3.04
4.04
0.85
3.08
3.06
6.04
6.04
f1 (ppm)

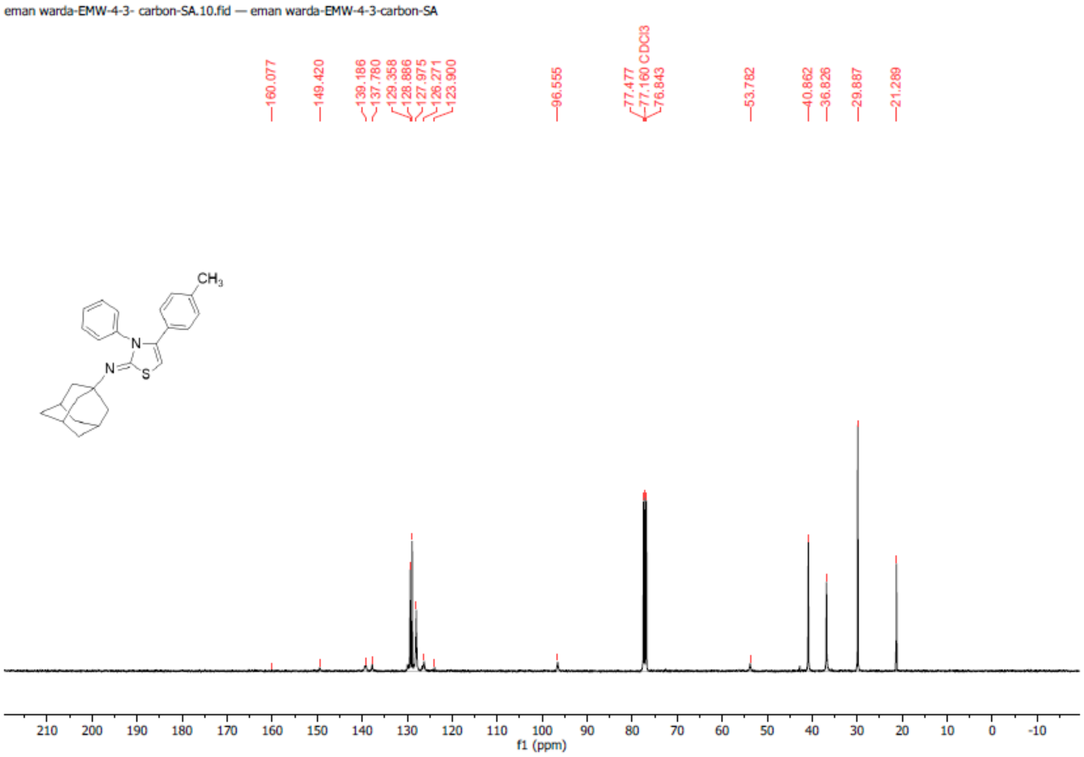
eman warda-EMW-4-3- carbon-SA.10.fid — eman warda-EMW-4-3-carbon-SA
160.077
149.420
139.186
137.780
129.358
128.886
127.975
126.271
123.900
96.555
77.477
77.160 CDCl3
76.843
53.782
40.862
36.826
29.887
21.289
CH3
N
N
S
f1 (ppm)

Compound **5e**

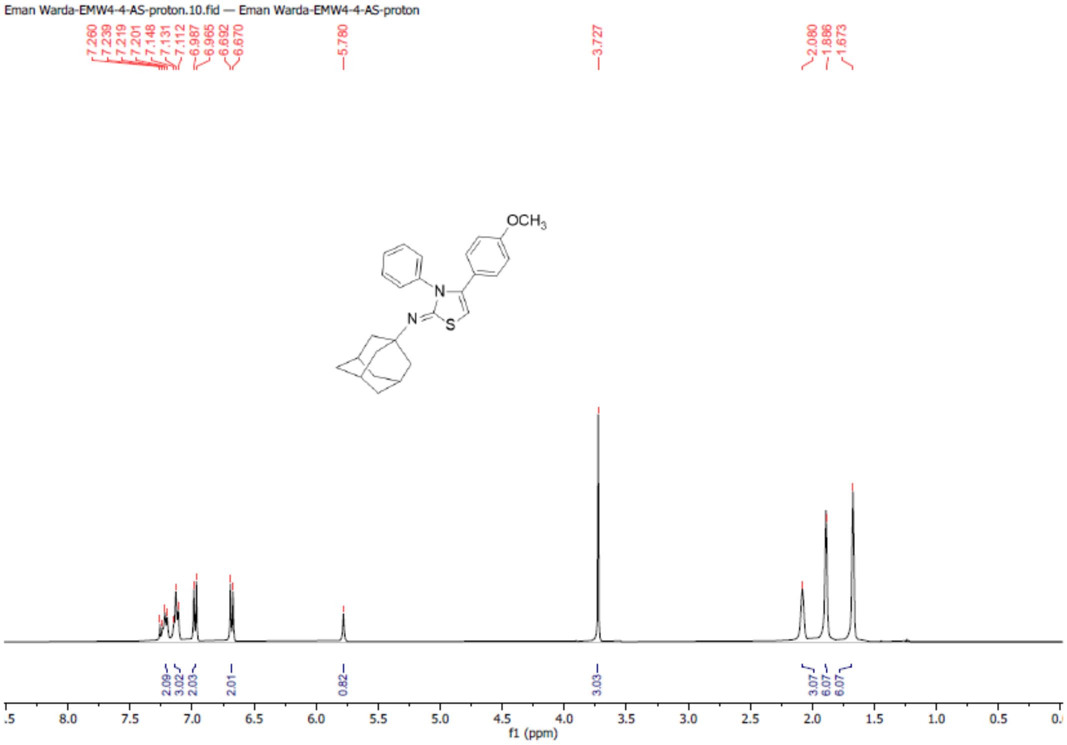
Eman Warda-EMW4-4-AS-proton.10.fid — Eman Warda-EMW4-4-AS-proton
7.260
7.239
7.219
7.201
7.148
7.131
7.112
6.987
6.965
6.692
6.670
5.780
3.727
2.080
1.886
1.673
OCH3
N
N
S
2.09
3.02
2.03
2.01
0.82
3.03
3.07
6.07
6.07
f1 (ppm)

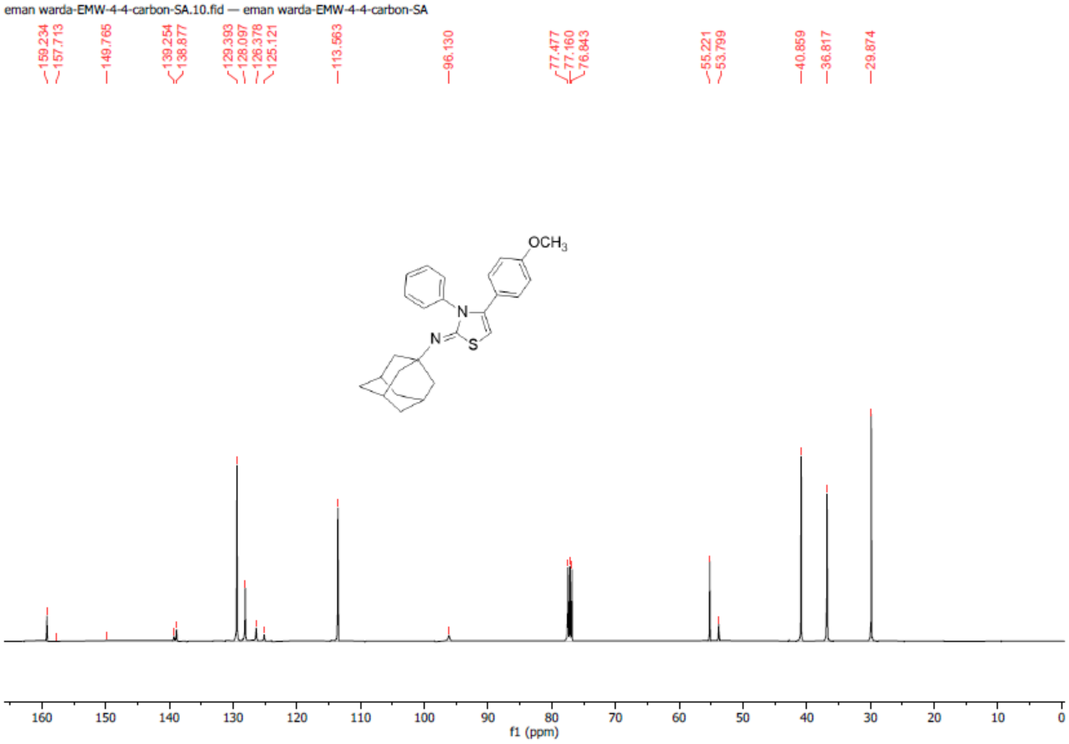
eman warda-EMW-4-4-carbon-SA.10.fid — eman warda-EMW-4-4-carbon-SA
159.234
157.713
149.765
139.254
138.877
129.393
128.097
126.378
125.121
113.563
96.130
77.477
77.160
76.843
55.221
53.799
40.859
36.817
29.874
OCH3
N
N
S
f1 (ppm)

Compound **5f**

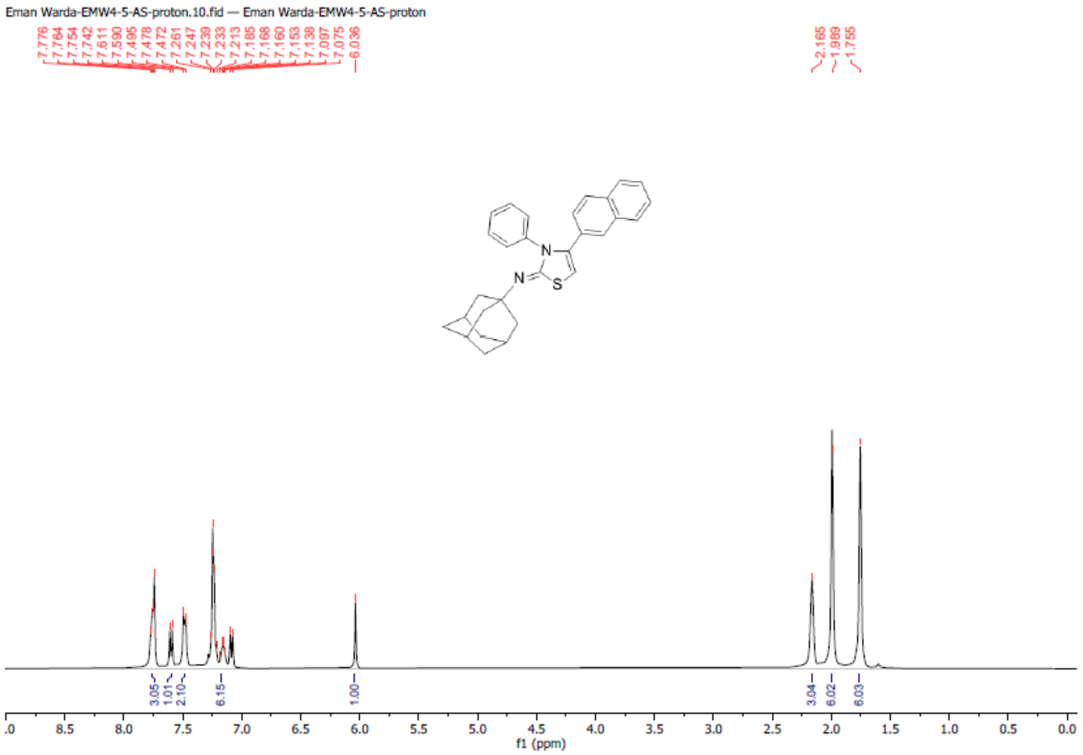
Eman Warda-EMW4-5-AS-proton.10.fid — Eman Warda-EMW4-5-AS-proton
7.776
7.764
7.754
7.742
7.611
7.590
7.495
7.478
7.472
7.261
7.247
7.239
7.233
7.213
7.185
7.168
7.160
7.153
7.138
7.097
7.075
6.036
2.165
1.989
1.755
N
S
3.05
1.01
2.10
6.15
1.00
3.04
6.02
6.03
f1 (ppm)

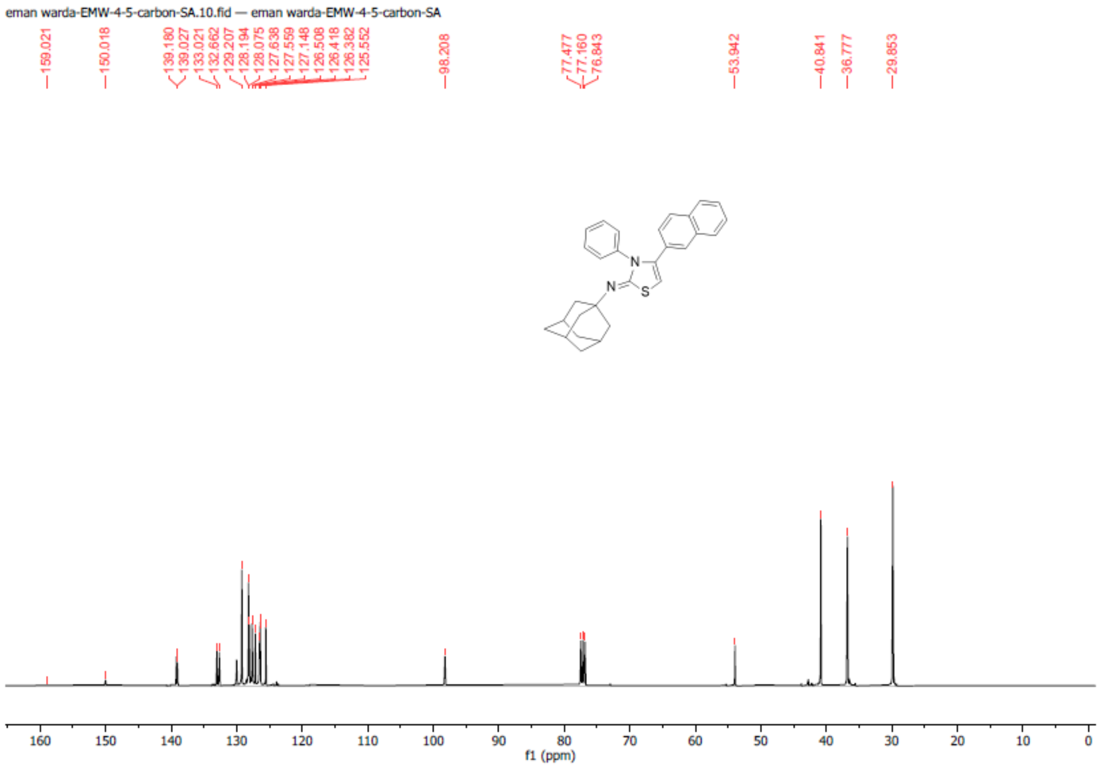
eman warda-EMW-4-5-carbon-SA.10.fid — eman warda-EMW-4-5-carbon-SA
159.021
150.018
139.180
139.027
133.021
132.662
129.207
128.194
128.075
127.638
127.559
127.148
126.508
126.418
126.382
125.552
98.208
77.477
77.160
76.843
53.942
40.841
36.777
29.853
N
S
f1 (ppm)

Compound **5g**

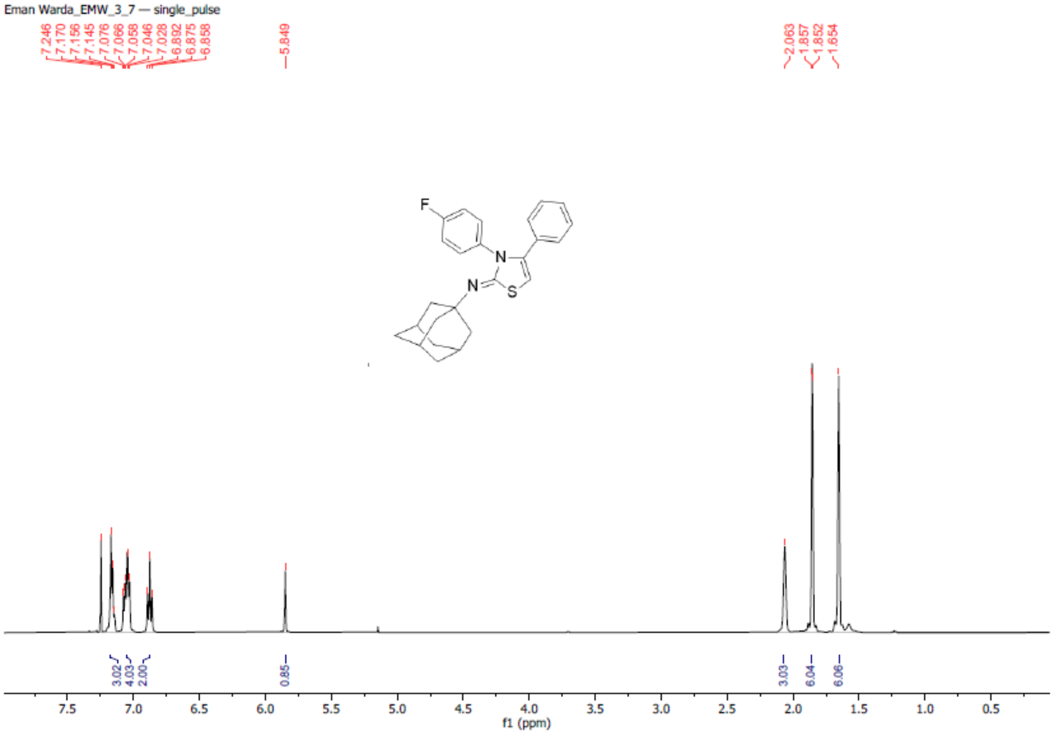

Eman Warda_EMW_3_7 — single_pulse
7.246
7.170
7.156
7.145
7.076
7.066
7.058
7.046
7.028
6.892
6.875
6.858
5.849
2.063
1.857
1.852
1.654
F
N
N
S
3.02
4.03
2.00
0.85
3.03
6.04
6.06
f1 (ppm)

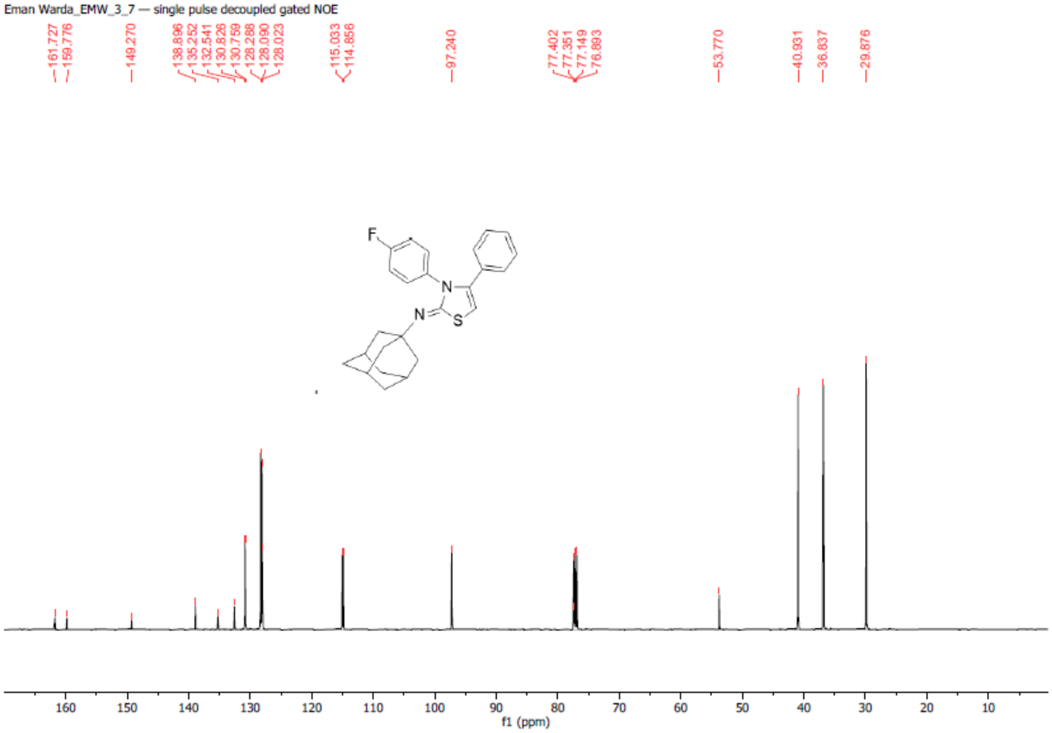

Eman Warda_EMW_3_7 — single pulse decoupled gated NOE
161.727
159.776
149.270
138.896
135.252
132.541
130.826
130.759
128.288
128.090
128.023
115.033
114.856
97.240
77.402
77.351
77.149
76.893
53.770
40.931
36.837
29.876
F
N
N
S
f1 (ppm)

Compound **5h**

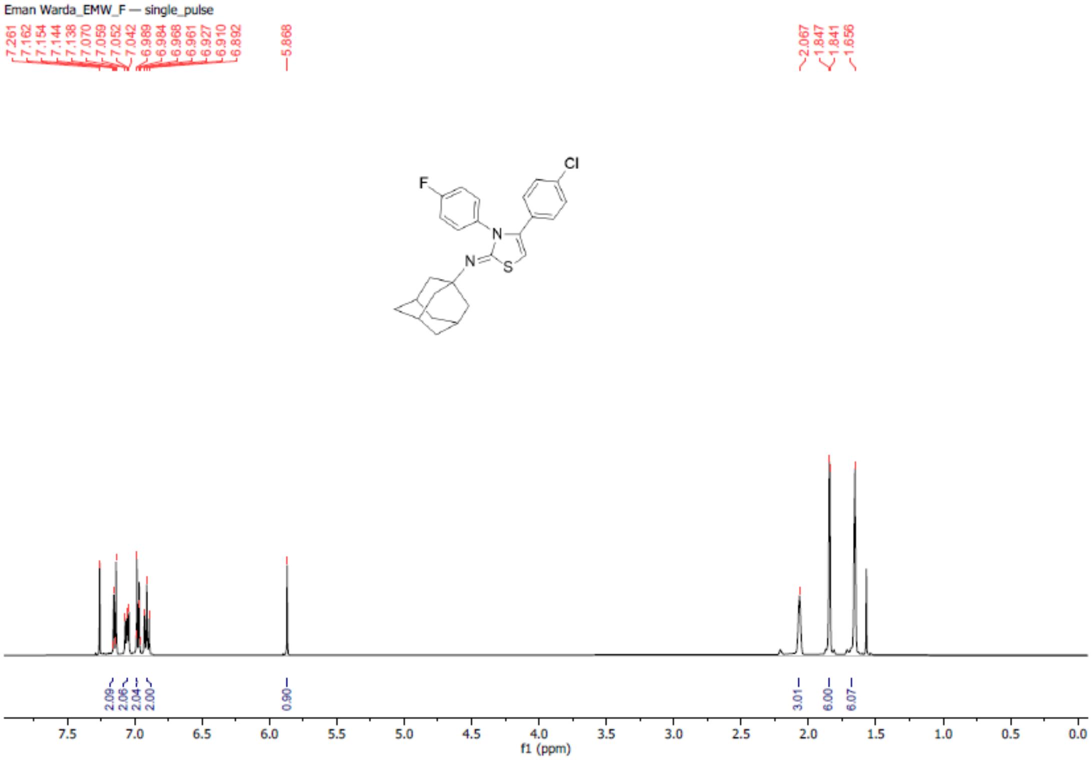
Eman Warda_EMW_F — single_pulse
f1 (ppm)

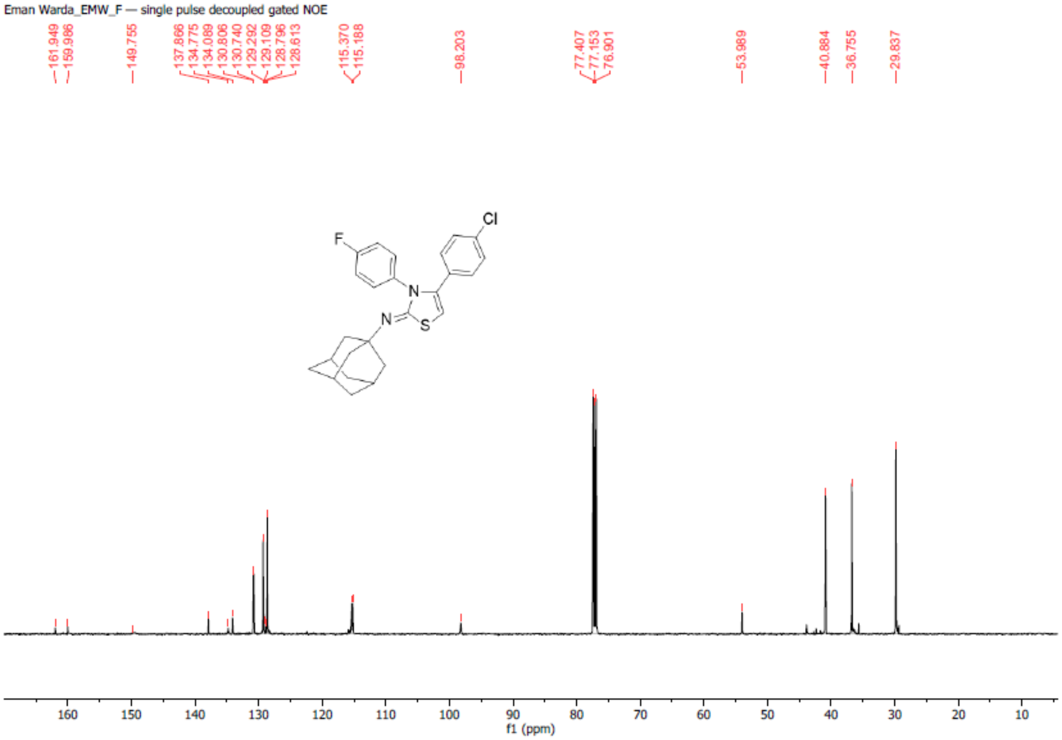
Eman Warda_EMW_F — single pulse decoupled gated NOE
f1 (ppm)

Compound **5i**

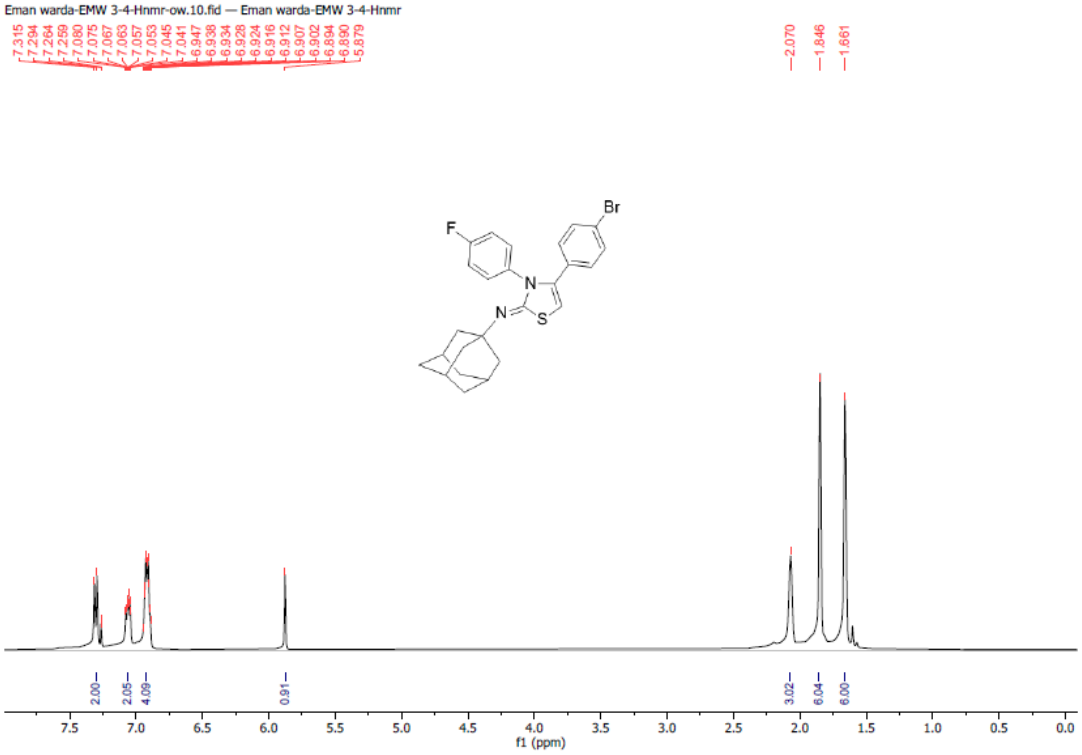
Eman warda-EMW 3-4-Hnmr-ow.10.fid — Eman warda-EMW 3-4-Hnmr
7.315
7.294
7.264
7.259
7.080
7.075
7.067
7.063
7.057
7.053
7.045
7.041
6.947
6.938
6.934
6.928
6.924
6.916
6.912
6.907
6.902
6.894
6.890
5.879
2.070
1.846
1.661
F
Br
N
N
S
2.00
2.05
4.09
0.91
3.02
6.04
6.00
f1 (ppm)

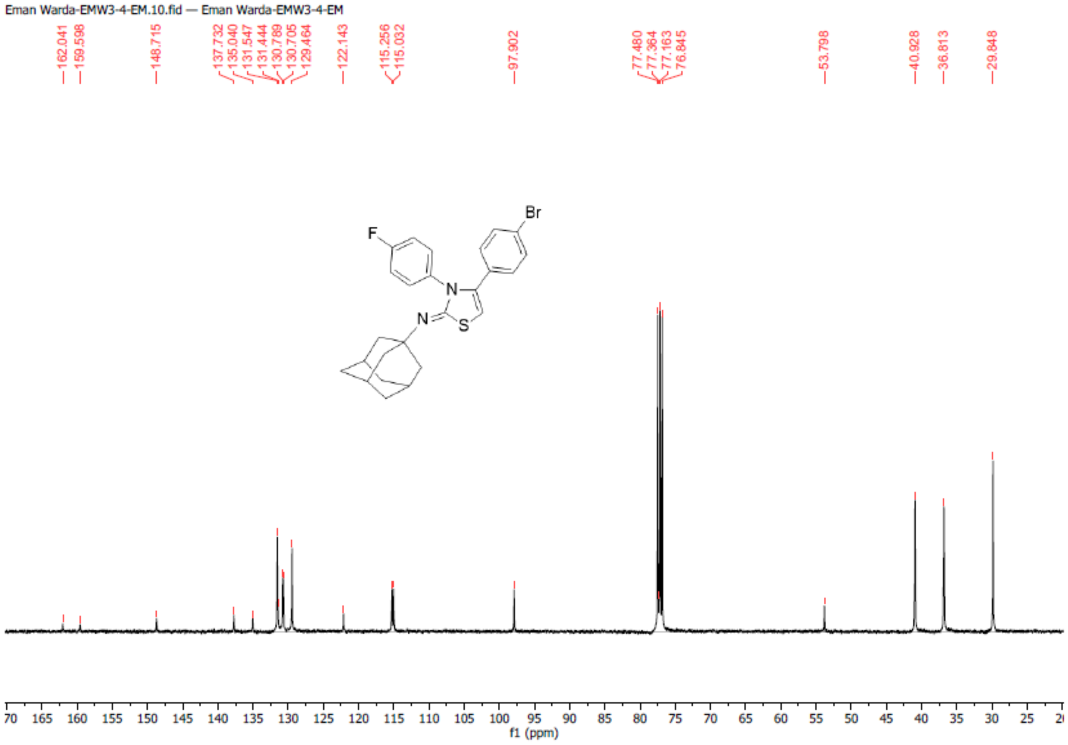
Eman Warda-EMW3-4-EM.10.fid — Eman Warda-EMW3-4-EM
162.041
159.598
148.715
137.732
135.040
131.547
131.444
130.789
130.705
129.464
122.143
115.256
115.032
97.902
77.480
77.364
77.163
76.845
53.798
40.928
36.813
29.848
F
Br
N
N
S
f1 (ppm)

Compound **5j**

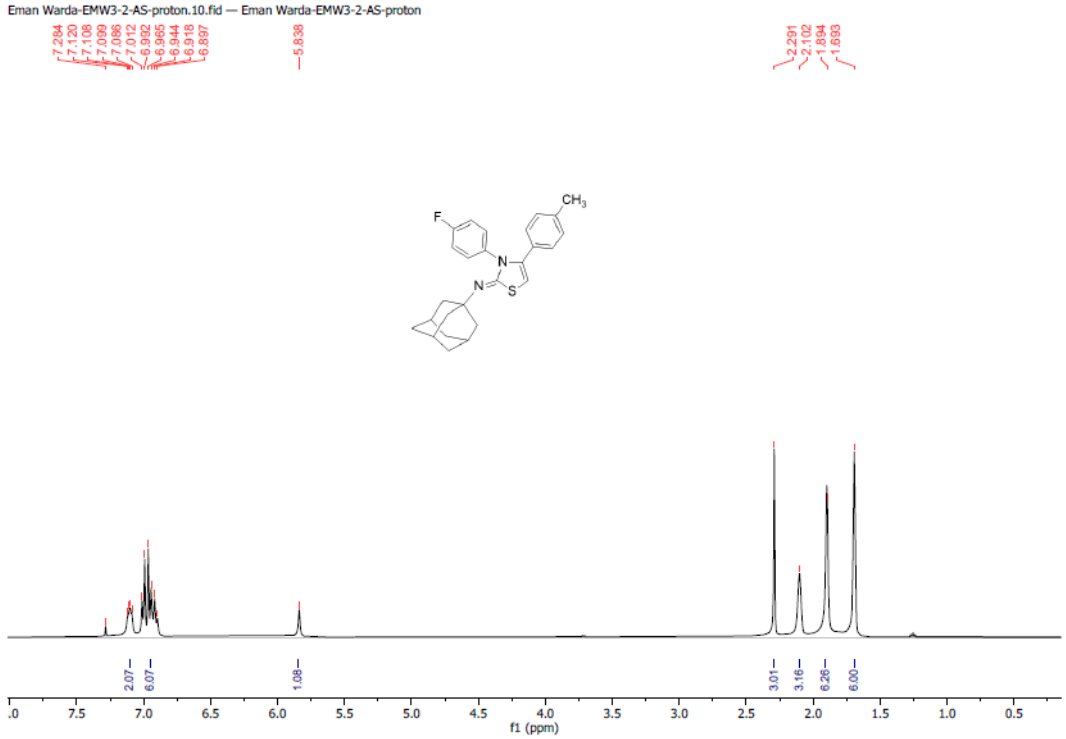
Eman Warda-EMW3-2-AS-proton.10.fid — Eman Warda-EMW3-2-AS-proton
7.284
7.120
7.108
7.099
7.086
7.012
6.992
6.965
6.944
6.918
6.897
5.838
2.291
2.102
1.894
1.693
F
N
N
S
CH3
2.07
6.07
1.08
3.01
3.16
6.26
6.00
f1 (ppm)

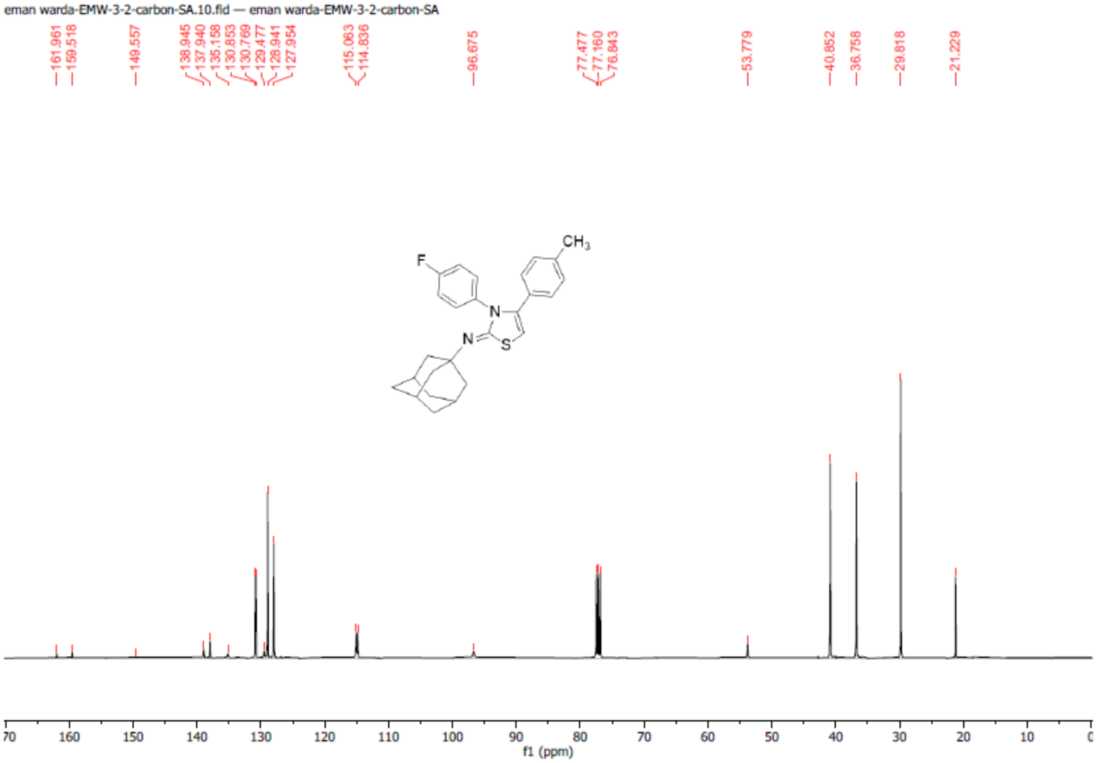
eman warda-EMW-3-2-carbon-SA.10.fid — eman warda-EMW-3-2-carbon-SA
161.961
159.518
149.557
138.945
137.940
135.158
130.853
130.769
129.477
128.941
127.954
115.063
114.836
96.675
77.477
77.160
76.843
53.779
40.852
36.758
29.818
21.229
F
N
N
S
CH3
f1 (ppm)

Compound **5k**

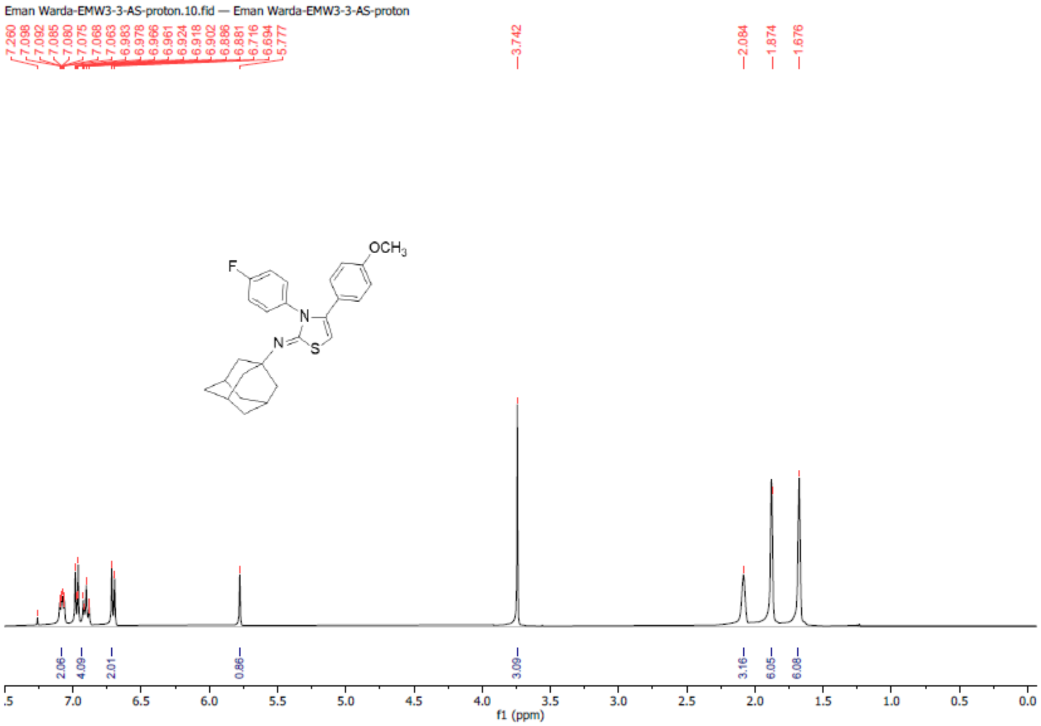
Eman Warda-EMW3-3-AS-proton.10.fid — Eman Warda-EMW3-3-AS-proton
7.260
7.098
7.092
7.085
7.080
7.075
7.068
7.063
6.983
6.978
6.966
6.961
6.924
6.918
6.902
6.886
6.881
6.716
6.694
5.777
3.742
2.084
1.874
1.676
F
N
OCH3
N
S
2.06
4.09
2.01
0.86
3.09
3.16
6.05
6.08
f1 (ppm)

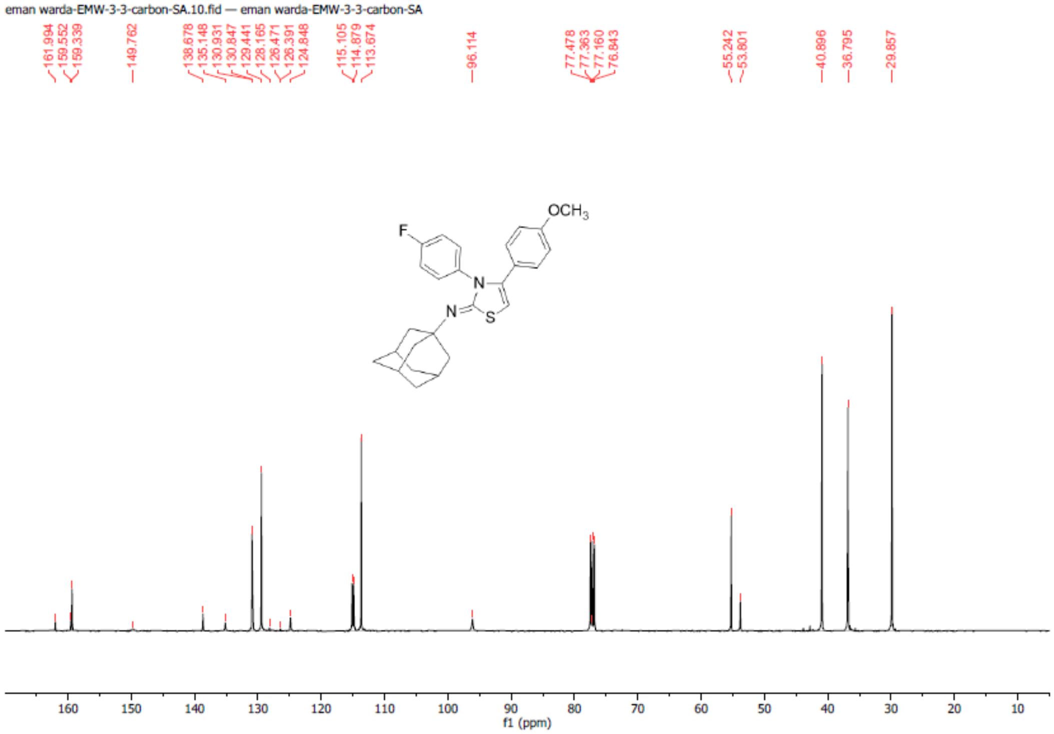
eman warda-EMW-3-3-carbon-SA.10.fid — eman warda-EMW-3-3-carbon-SA
161.994
159.552
159.339
149.762
138.678
135.148
130.931
130.847
129.441
128.165
126.471
126.391
124.848
115.105
114.879
113.674
96.114
77.478
77.363
77.160
76.843
55.242
53.801
40.896
36.795
29.857
F
N
OCH3
N
S
f1 (ppm)

Compound **5l**

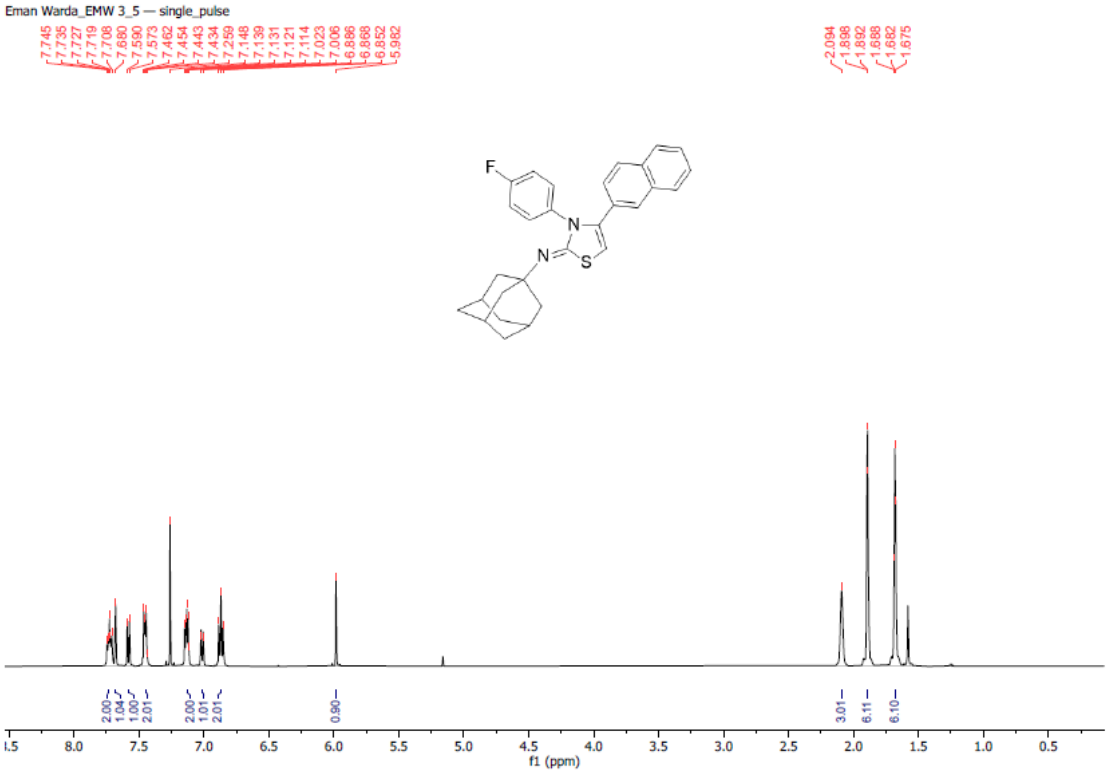
Eman Warda_EMW 3_5 — single_pulse
f1 (ppm)

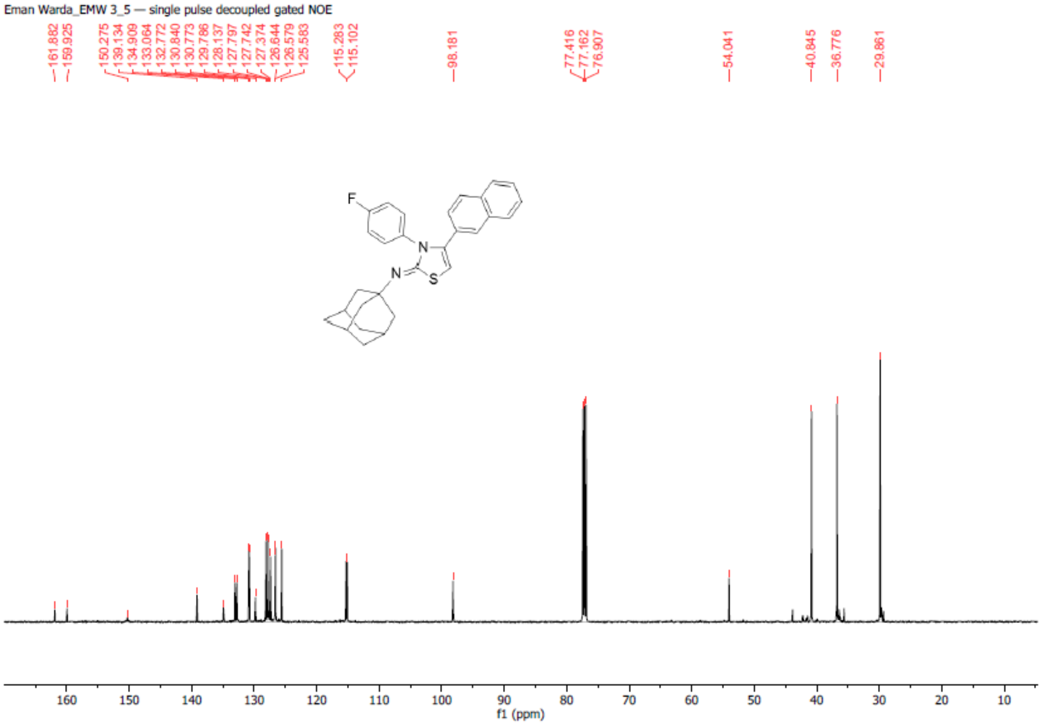
Eman Warda_EMW 3_5 — single pulse decoupled gated NOE
f1 (ppm)

Compound **5m**

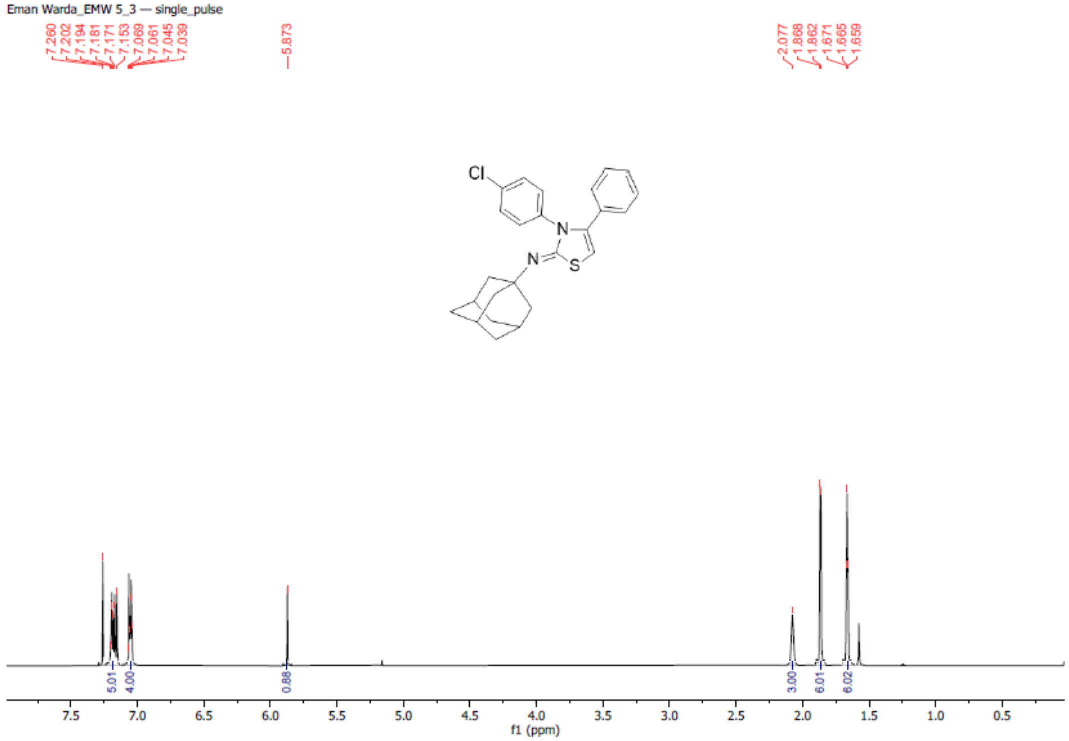

Eman Warda_EMW 5_3 — single_pulse
7.260
7.202
7.194
7.181
7.171
7.153
7.069
7.061
7.045
7.039
5.873
2.077
1.868
1.862
1.671
1.665
1.659
Cl
N
N
S
5.01
4.00
0.88
3.00
6.01
6.02
f1 (ppm)

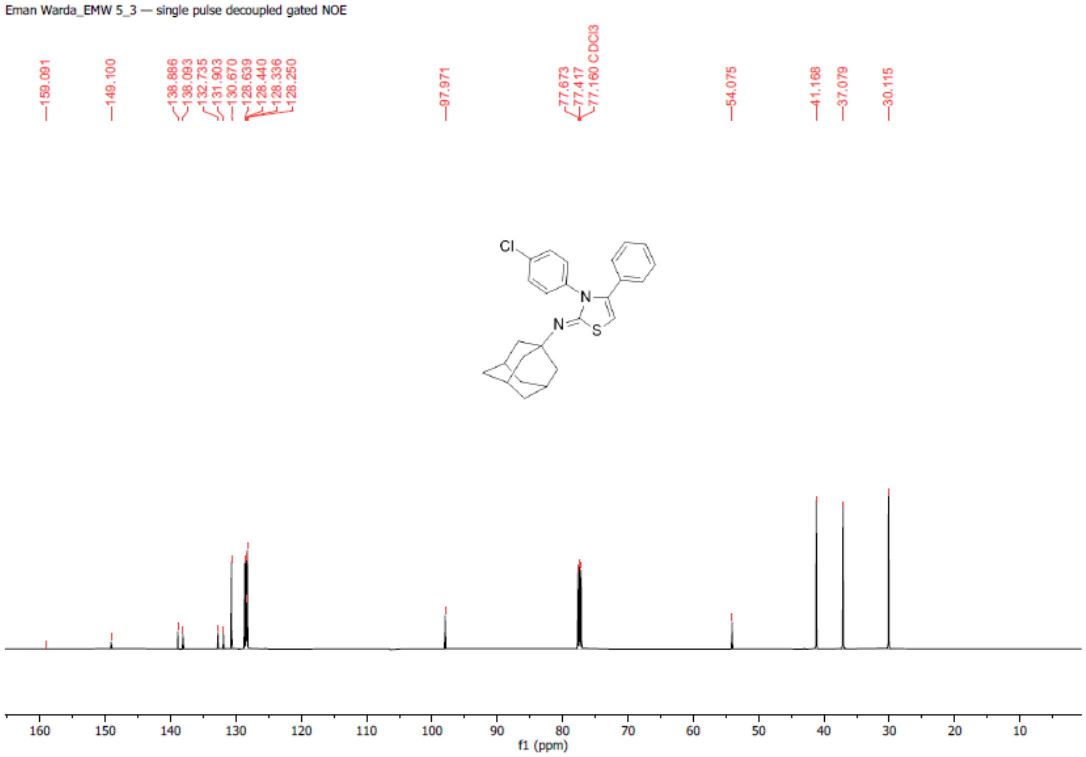

Eman Warda_EMW 5_3 — single pulse decoupled gated NOE
159.091
149.100
138.886
138.093
132.735
131.903
130.670
128.639
128.440
128.336
128.250
97.971
77.673
77.417
77.160 CDCl3
54.075
41.168
37.079
30.115
Cl
N
N
S
f1 (ppm)

Compound **5n**

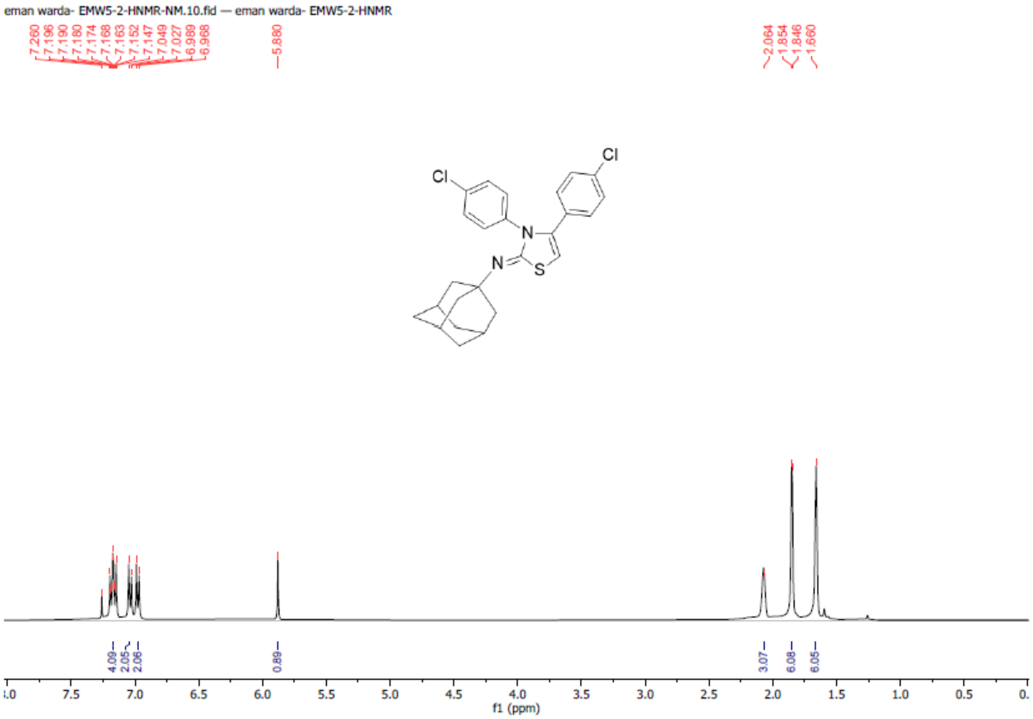
eman warda- EMW5-2-HNMR-NM.10.fid — eman warda- EMW5-2-HNMR
7.260
7.196
7.190
7.180
7.174
7.168
7.163
7.152
7.147
7.049
7.027
6.989
6.968
5.880
2.064
1.854
1.846
1.660
Cl
Cl
N
N
S
4.09
2.05
2.06
0.89
3.07
6.08
6.05
7.5
7.0
6.5
6.0
5.5
5.0
4.5
4.0
3.5
3.0
2.5
2.0
1.5
1.0
0.5
f1 (ppm)

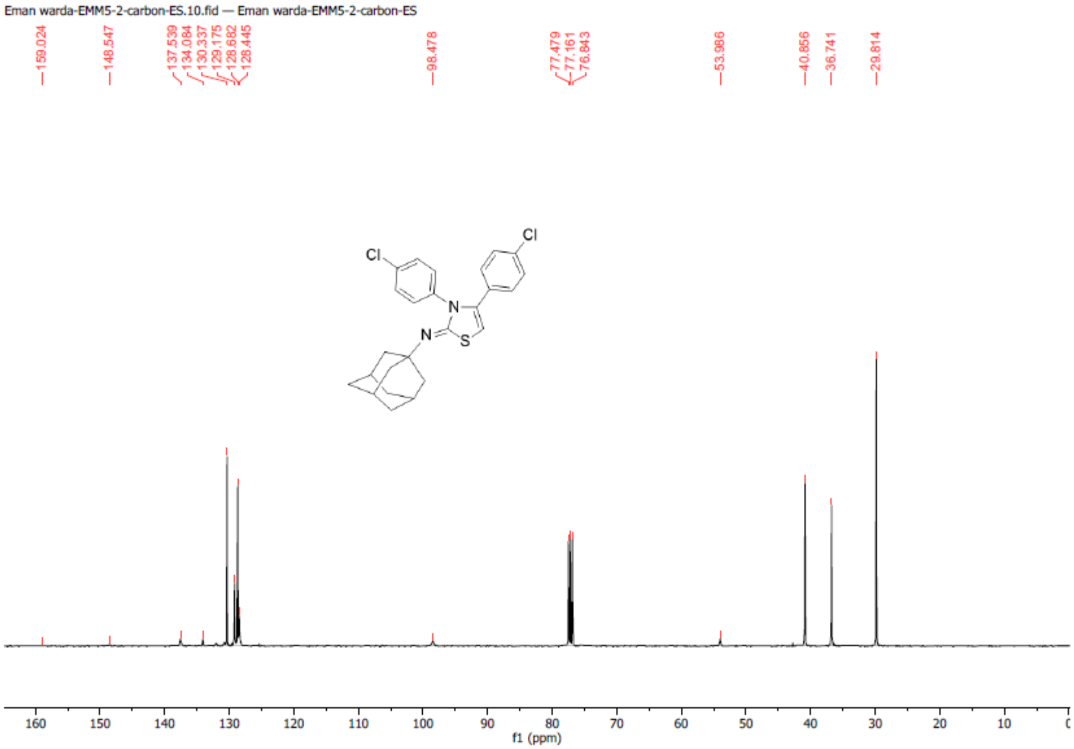
Eman warda-EMM5-2-carbon-ES.10.fid — Eman warda-EMM5-2-carbon-ES
159.024
148.547
137.539
134.084
130.337
129.175
128.682
128.445
98.478
77.479
77.161
76.843
53.986
40.856
36.741
29.814
Cl
Cl
N
N
S
160
150
140
130
120
110
100
90
80
70
60
50
40
30
20
10
f1 (ppm)

Compound **5o**

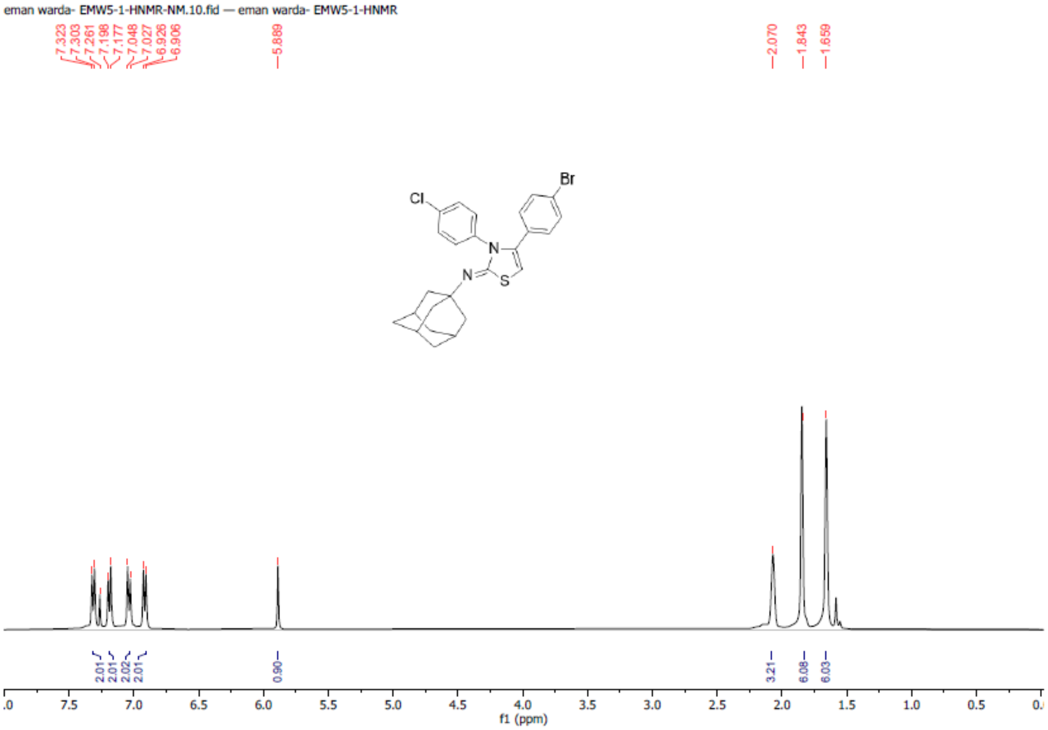
eman warda- EMW5-1-HNMR-NM.10.fid — eman warda- EMW5-1-HNMR
7.323
7.303
7.261
7.198
7.177
7.048
7.027
6.926
6.906
5.889
2.070
1.843
1.659
Br
Cl
N
N
S
2.01
2.01
2.02
2.01
0.90
3.21
6.08
6.03
f1 (ppm)

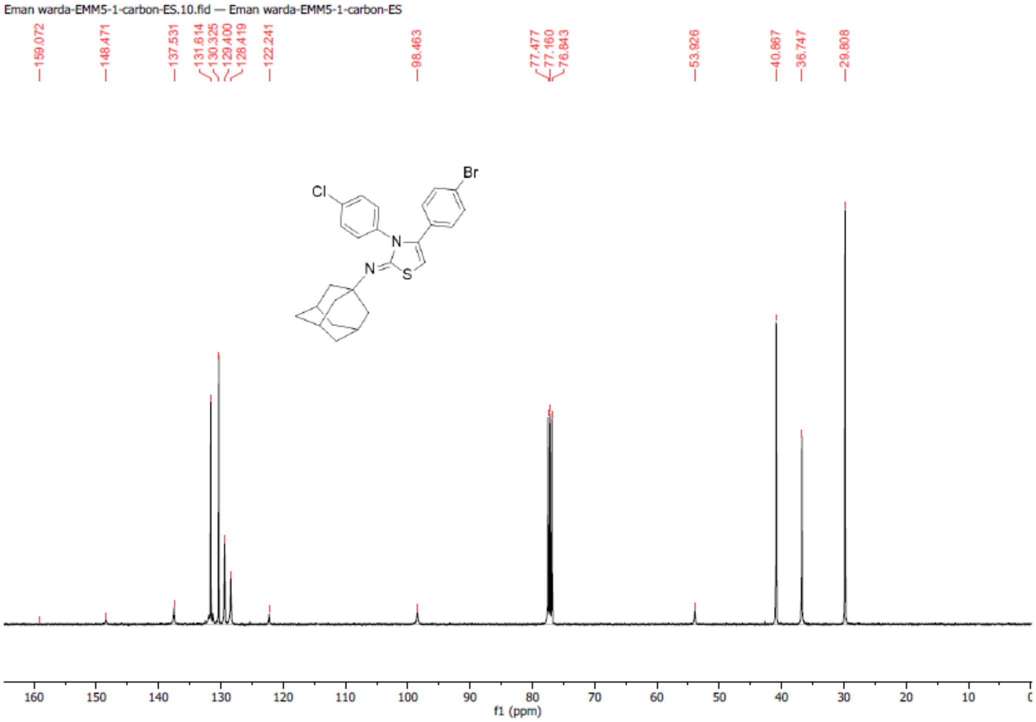
Eman warda-EMM5-1-carbon-ES.10.fid — Eman warda-EMM5-1-carbon-ES
159.072
148.471
137.531
131.614
130.325
129.400
128.419
122.241
98.463
77.477
77.160
76.843
53.926
40.867
36.747
29.808
Br
Cl
N
N
S
f1 (ppm)

Compound **5p**

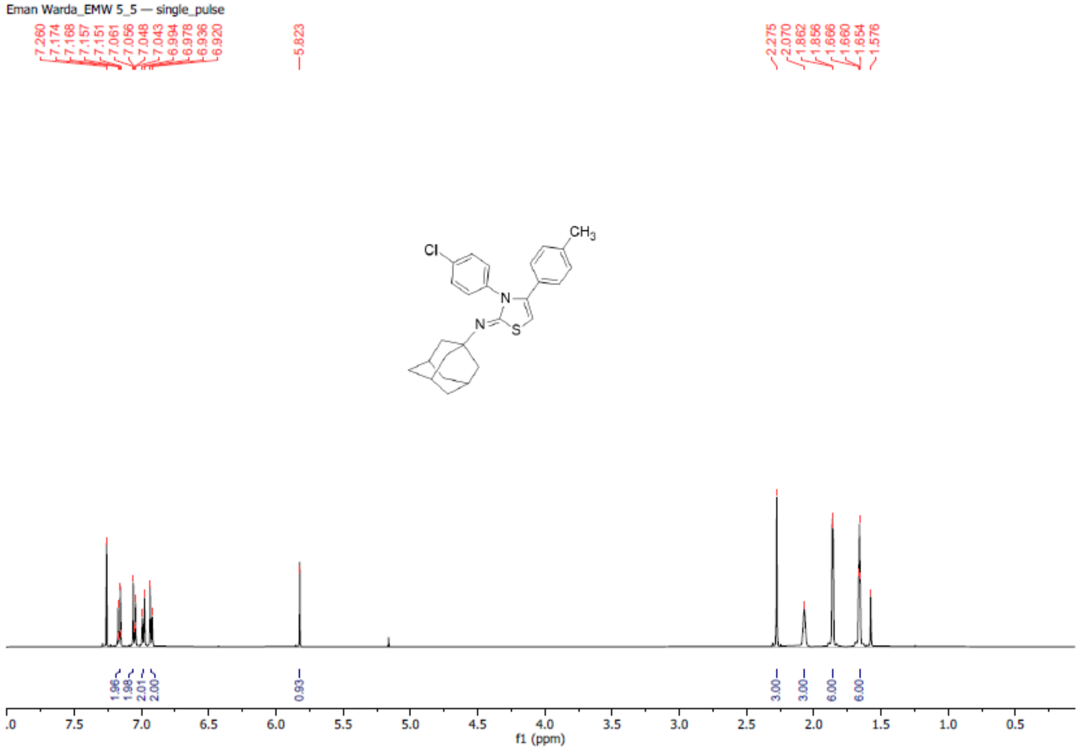
Eman Warda_EMW 5_5 — single_pulse
7.260
7.174
7.168
7.157
7.151
7.061
7.056
7.048
7.043
6.994
6.978
6.936
6.920
5.823
2.275
2.070
1.862
1.856
1.666
1.660
1.654
1.576
CH3
Cl
N
N
S
1.96
1.98
2.01
2.00
0.93
3.00
3.00
6.00
6.00
f1 (ppm)

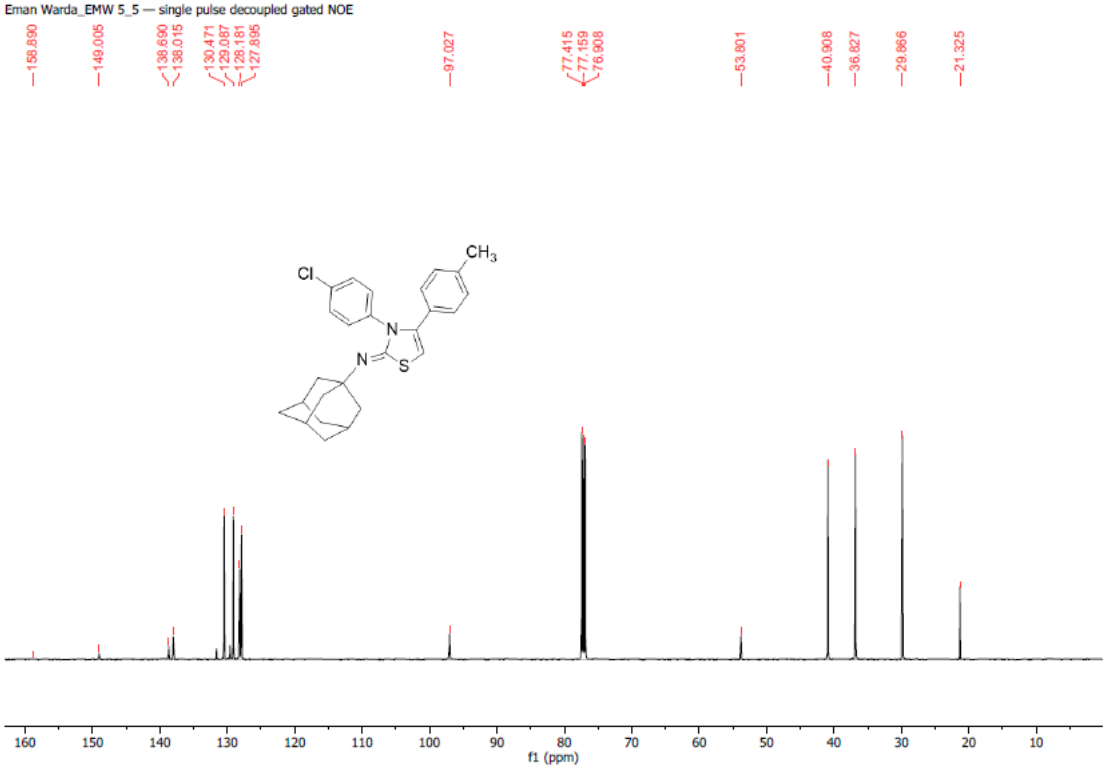
Eman Warda_EMW 5_5 — single pulse decoupled gated NOE
158.890
149.005
138.690
138.015
130.471
129.087
128.181
127.895
97.027
77.415
77.159
76.908
53.801
40.908
36.827
29.866
21.325
CH3
Cl
N
N
S
f1 (ppm)

Compound **5q**

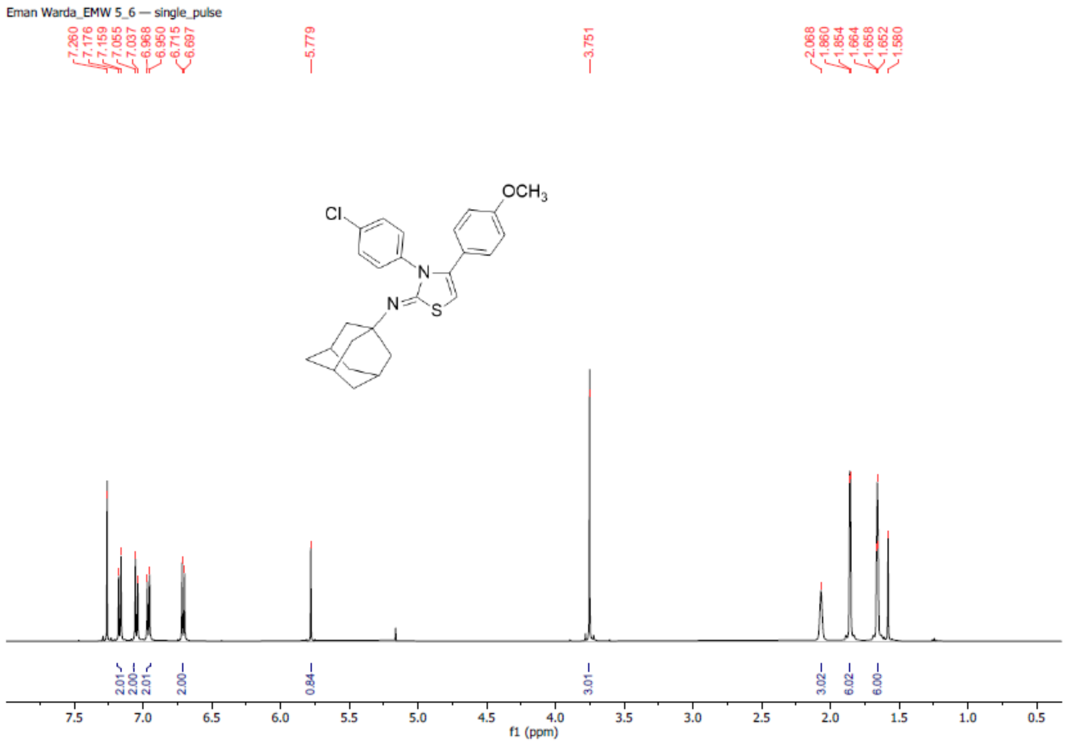
Eman Warda_EMW 5_6 — single_pulse
7.260
7.176
7.159
7.055
7.037
6.968
6.950
6.715
6.697
5.779
3.751
2.068
1.860
1.854
1.664
1.658
1.652
1.580
OCH3
Cl
N
N
S
2.01
2.00
2.01
2.00
0.84
3.01
3.02
6.02
6.00
f1 (ppm)

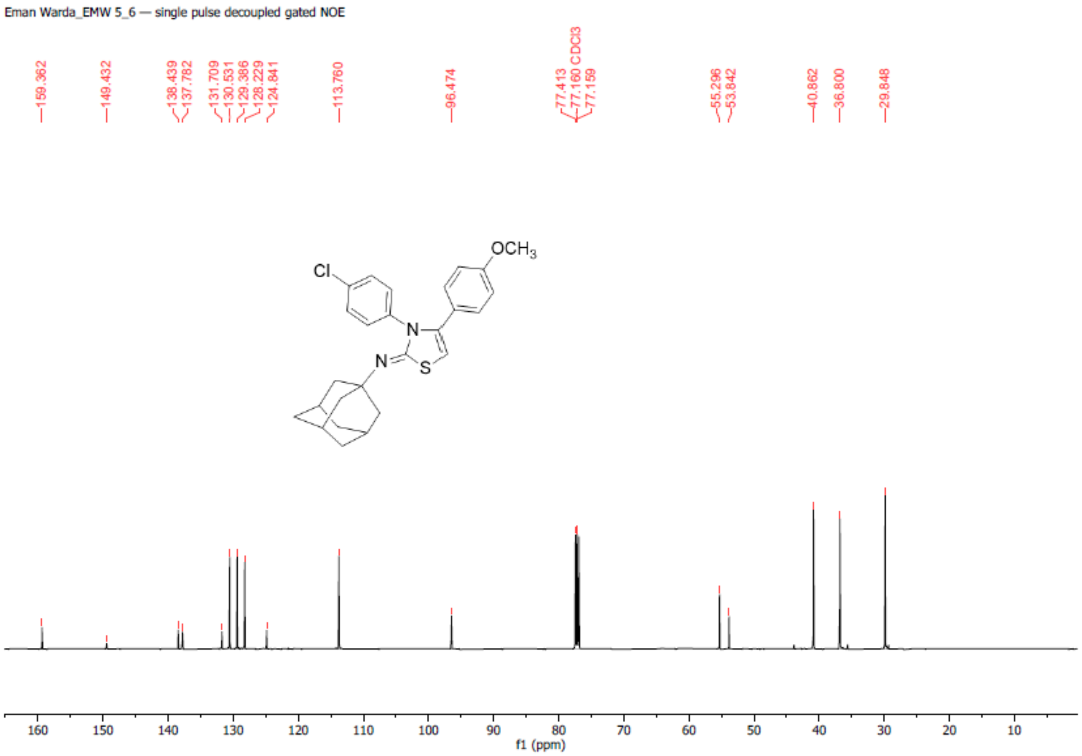
Eman Warda_EMW 5_6 — single pulse decoupled gated NOE
159.362
149.432
138.439
137.782
131.709
130.531
129.386
128.229
124.841
113.760
96.474
77.413
77.160 CDCl3
77.159
55.296
53.842
40.862
36.800
29.848
OCH3
Cl
N
N
S
f1 (ppm)

Compound **5r**

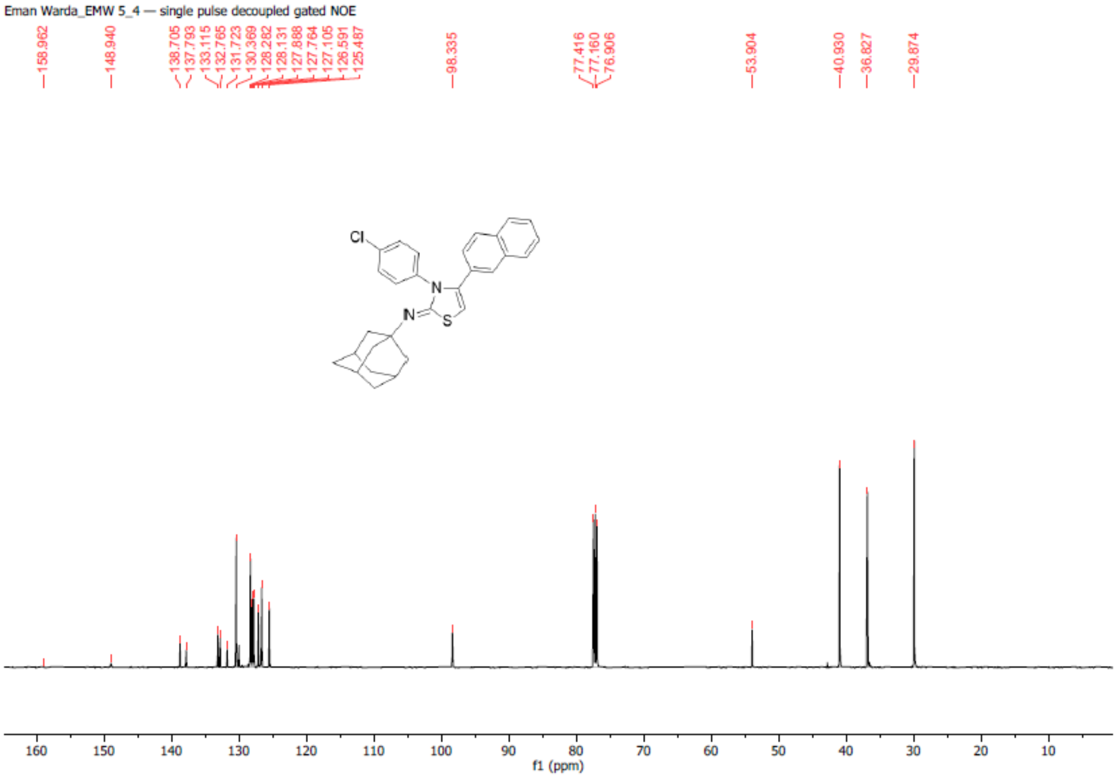
Eman Warda_EMW 5_4 — single pulse decoupled gated NOE
158.962
148.940
138.705
137.793
133.115
132.765
131.723
130.369
128.282
128.131
127.888
127.764
127.105
126.591
125.487
98.335
77.416
77.160
76.906
53.904
40.930
36.827
29.874
Cl
N
N
S
160 150 140 130 120 110 100 90 80 70 60 50 40 30 20 10
f1 (ppm)

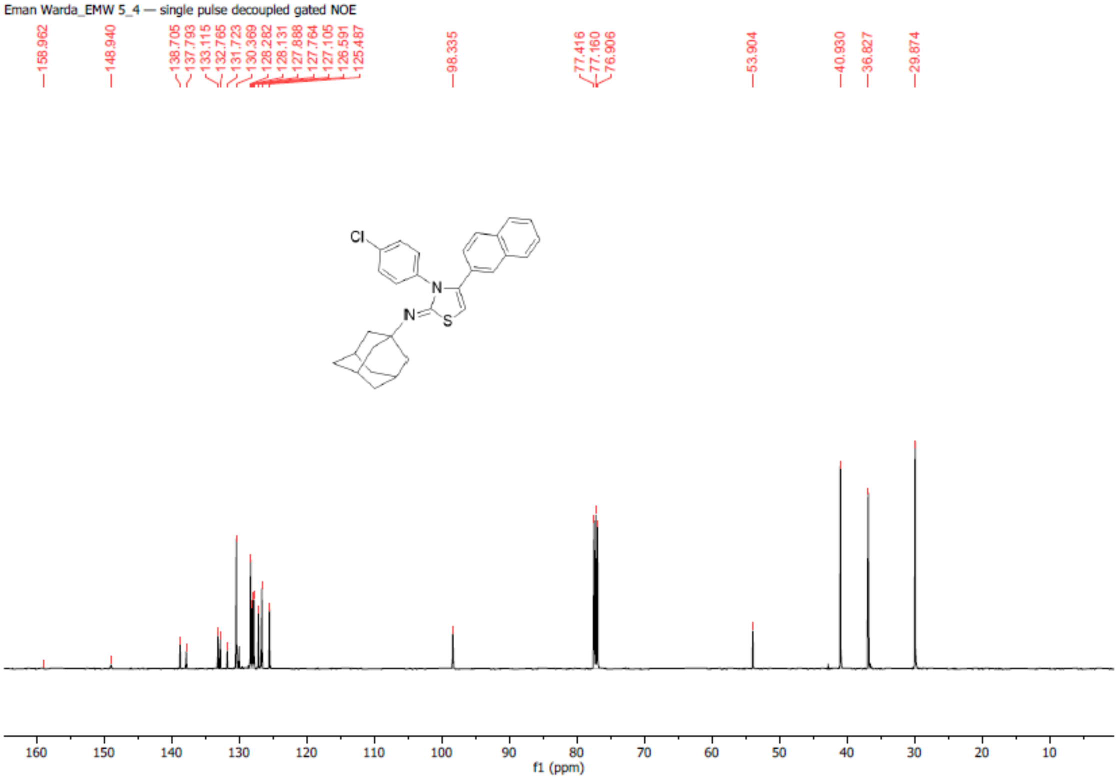
Eman Warda_EMW 5_4 — single pulse decoupled gated NOE
158.962
148.940
138.705
137.793
133.115
132.765
131.723
130.369
128.282
128.131
127.888
127.764
127.105
126.591
125.487
98.335
77.416
77.160
76.906
53.904
40.930
36.827
29.874
Cl
N
N
S
160 150 140 130 120 110 100 90 80 70 60 50 40 30 20 10
f1 (ppm)

Determination of antimicrobial activity of compounds 5a-r (agar disc-diffusion method)

Sterile filter paper discs (8 mm diameter) were moistened with compounds **5a-r** solution in dimethyl sulphoxide of specific concentration (200 µg/disc), the broad-spectrum antibacterial drugs Ampicillin trihydrate, Ciprofloxacin and the antifungal drug Fluconazole (100 µg/disc) were carefully placed on the agar culture plates that had been previously inoculated separately with the microorganisms. The plates were incubated at 37 ºC, and the diameter of the growth inhibition zones were measured after 24 hours in case of bacteria and 48 hours in case of fungi.

Determination of minimal inhibitory concentrations (MIC) and the minimal biocidal concentrations (MBC) for compounds 5a, 5c, 5g, 5l, 5m, 5o and 5q (micro-dilution susceptibility method)

Compounds **5a**, **5c**, **5g**, **5l**, **5m**, **5o** and **5q**, Ampicillin trihydrate, Ciprofloxacin and Fluconazole were dissolved in dimethylsulphoxide at concentration of 128 µg/mL. Two-fold dilutions of the solution were prepared (128, 64, 32, …, 0.5 µg/mL). The microorganism suspensions at 10^6 CFU/mL (colony forming unit/ml) concentrations were inoculated to the corresponding wells. The plates were incubated at 36 ºC for 24 and 48 hours for the bacteria and *Candida albicans*, respectively. The MIC values were determined as the lowest concentration that completely inhibited visible growth of the microorganism as detected by unaided eye. The MBC values were determined by the lowest concentration that killed of the microorganism by re-cultured on agar medium to verify the absence of growth.

Determination of bacterial biofilm inhibitory activity of compounds 5a, 5c, 5l and 5o (crystal violet staining method).

The effect of the synthesized compounds on biofilm formation was assessed in 96-well polystyrene plates. *Staphylococcus aureus* IFO 3060 and *Micrococcus luteus* IFO 3232 cells were inoculated into tryptone soy broth (TSB), after static incubation, cultures were vortexed, diluted 1:100 in TSB a final concentration 10^6 CFU/mL for biofilm formation and were cultured statically with or without the compounds **5a**, **5c, 5l and 5o** for 24 hours at 37 ºC. Then, non-adherent bacteria were detached by washing with sterile phosphate-buffered saline (PBS), and adherent bacteria were stained for 15 minutes with a 1% crystal violet solution. After 30 minutes. Then, 100 µL dissolving solution (30% methanol and 10% acetic acid) was added to each well to dissolve the crystal violet, and the optical density (OD) was measured at 570 nm using a microplate reader. The wells

containing medium supplemented with PBS were used as negative controls. Erythromycin, was used as a positive control. The intensity of staining is directly proportional to the number of biofilm adhering to the 96-well plate. The biofilm inhibition rate was calculated as follows:

% Bacterial biofilm inhibition = [(OD of control – OD of test compound/OD of control)] x 100

The IC_{50} is the lowest concentrations of tested compounds that exhibited 50% inhibition on the biofilm formation.

Determination of *in vitro* anti-proliferative activity (MTT assay)

The tumor cells (3000 cells per well) were cultured and seeded into 96-well plates and the plates were incubated for 24 hours. The cells were then treated with compounds **5a-r** and Doxorubicin at different concentrations in dimethyl sulfoxide (0.1 μM to 100 μM) at 37 °C in an atmosphere of 5% CO_2 for 48 hours. Freshly prepared 3-[4,5-dimethylthiazoyl-2-yl]-2,5-diphenyltetrazolium bromide (MTT) was added to each well at a terminal concentration of 5 μg/mL and incubated with cells at 37 °C for 4 hours. The formazan crystals were dissolved in 100 μL of dimethyl sulfoxide in each well, and the absorbency at 492 nm (for absorbance of MTT formazan) and 630 nm (for the reference wavelength) was measured with an enzyme linked immunosorbent assay (ELISA) reader (ChroMate-4300, FL, USA). All compounds were tested three times in each of the cell lines. The IC_{50} values were calculated according to the equation for Boltzmann sigmoidal concentration response curve using the nonlinear regression fitting models (Graph Pad, Prism Version 5). The results reported are means of three separate experiments. Statistical differences were analysed according to one-way ANOVA test wherein the differences were considered to be significant at $p < 0.05$.

checkCIF/PLATON report

Structure factors have been supplied for datablock(s) EMW-3

THIS REPORT IS FOR GUIDANCE ONLY. IF USED AS PART OF A REVIEW PROCEDURE FOR PUBLICATION, IT SHOULD NOT REPLACE THE EXPERTISE OF AN EXPERIENCED CRYSTALLOGRAPHIC REFEREE.

No syntax errors found. CIF dictionary Interpreting this report

Datablock: EMW-3

```
Bond precision:    C-C = 0.0051 A           Wavelength=1.54184

Cell:              a=18.7865(3)     b=10.8565(2)       c=23.2644(5)
                   alpha=90         beta=92.425(2)     gamma=90
Temperature:       121 K

                 Calculated                      Reported
Volume           4740.66(15)                     4740.66(15)
Space group      I 2/a                           I 2/a
Hall group       -I 2ya                          -I 2ya
Moiety formula   C26 H28 N2 S, 0.5(C6 H12)       C26 H28 N2 S, 0.5(C6 H12)
Sum formula      C29 H34 N2 S                    C29 H34 N2 S
Mr               442.64                          442.64
Dx,g cm-3        1.240                           1.240
Z                8                               8
Mu (mm-1)        1.341                           1.341
F000             1904.0                          1904.0
F000'            1911.09
h,k,lmax         23,13,29                        23,13,29
Nref             4882                            4882
Tmin,Tmax        0.851,0.886                     0.851,0.886
Tmin'            0.818

Correction method= # Reported T Limits: Tmin=0.851 Tmax=0.886
AbsCorr = MULTI-SCAN

Data completeness= 1.000           Theta(max)= 74.813

R(reflections)= 0.0688( 3722)                          wR2(reflections)=
                                                       0.2061( 4793)
S = 1.041                   Npar= 290
```

The following ALERTS were generated. Each ALERT has the format
test-name_ALERT_alert-type_alert-level.
Click on the hyperlinks for more details of the test.

Alert level C

```
PLAT243_ALERT_4_C High   'Solvent' Ueq as Compared to Neighbors of       C29 Check
PLAT260_ALERT_2_C Large Average Ueq of Residue Including       C27      0.109 Check
PLAT340_ALERT_3_C Low Bond Precision on  C-C Bonds ..............     0.0051 Ang.
PLAT360_ALERT_2_C Short  C(sp3)-C(sp3) Bond  C28      - C29      .       1.42 Ang.
PLAT906_ALERT_3_C Large K Value in the Analysis of Variance ......      3.103 Check
PLAT977_ALERT_2_C Check Negative Difference Density on H27A      .      -0.34 eA-3
PLAT977_ALERT_2_C Check Negative Difference Density on H27B      .      -0.34 eA-3
PLAT977_ALERT_2_C Check Negative Difference Density on H28B      .      -0.31 eA-3
PLAT977_ALERT_2_C Check Negative Difference Density on H29A      .      -0.38 eA-3
PLAT977_ALERT_2_C Check Negative Difference Density on H30A      .      -0.36 eA-3
PLAT977_ALERT_2_C Check Negative Difference Density on H30B      .      -0.36 eA-3
```

Alert level G

```
PLAT066_ALERT_1_G Predicted and Reported Tmin&Tmax Range Identical          ? Check
PLAT072_ALERT_2_G SHELXL First  Parameter in WGHT  Unusually Large       0.10 Report
PLAT083_ALERT_2_G SHELXL Second Parameter in WGHT  Unusually Large      11.70 Why ?
PLAT300_ALERT_4_G Atom Site Occupancy of H27A       Constrained at        0.5 Check
PLAT300_ALERT_4_G Atom Site Occupancy of H27B       Constrained at        0.5 Check
PLAT300_ALERT_4_G Atom Site Occupancy of H30A       Constrained at        0.5 Check
PLAT300_ALERT_4_G Atom Site Occupancy of H30B       Constrained at        0.5 Check
PLAT380_ALERT_4_G Incorrectly? Oriented X(sp2)-Methyl Moiety .....        C10 Check
PLAT410_ALERT_2_G Short Intra H...H Contact  H27A      ..H28B     .       2.07 Ang.
                                                     x,y,z  =      1_555 Check
PLAT410_ALERT_2_G Short Intra H...H Contact  H27A      ..H28A     .       2.05 Ang.
                                               3/2-x,y,1-z  =      2_656 Check
PLAT410_ALERT_2_G Short Intra H...H Contact  H27B      ..H28A     .       2.06 Ang.
                                                     x,y,z  =      1_555 Check
PLAT410_ALERT_2_G Short Intra H...H Contact  H27B      ..H28B     .       2.07 Ang.
                                               3/2-x,y,1-z  =      2_656 Check
PLAT410_ALERT_2_G Short Intra H...H Contact  H29A      ..H30B     .       2.08 Ang.
                                                     x,y,z  =      1_555 Check
PLAT410_ALERT_2_G Short Intra H...H Contact  H29A      ..H30B     .       2.08 Ang.
                                                     x,y,z  =      1_555 Check
PLAT410_ALERT_2_G Short Intra H...H Contact  H29B      ..H30A     .       2.08 Ang.
                                                     x,y,z  =      1_555 Check
PLAT410_ALERT_2_G Short Intra H...H Contact  H29B      ..H30A     .       2.08 Ang.
                                                     x,y,z  =      1_555 Check
PLAT411_ALERT_2_G Short Inter H...H Contact  H15       ..H27B     .       2.12 Ang.
                                                     x,y,z  =      1_555 Check
PLAT411_ALERT_2_G Short Inter H...H Contact  H15       ..H27B     .       2.12 Ang.
                                                     x,y,z  =      1_555 Check
PLAT883_ALERT_1_G No Info/Value for _atom_sites_solution_primary .     Please Do !
PLAT912_ALERT_4_G Missing # of FCF Reflections Above STh/L=  0.600         88 Note
PLAT941_ALERT_3_G Average HKL Measurement Multiplicity ..........         3.8 Low
PLAT955_ALERT_1_G Reported (CIF) and Actual (FCF) Lmax Differ by .          1 Units
PLAT978_ALERT_2_G Number C-C Bonds with Positive Residual Density.          5 Info
```

---

0 **ALERT level A** = Most likely a serious problem - resolve or explain

```
 0 ALERT level B = A potentially serious problem, consider carefully
11 ALERT level C = Check. Ensure it is not caused by an omission or oversight
23 ALERT level G = General information/check it is not something unexpected

 3 ALERT type 1 CIF construction/syntax error, inconsistent or missing data
21 ALERT type 2 Indicator that the structure model may be wrong or deficient
 3 ALERT type 3 Indicator that the structure quality may be low
 7 ALERT type 4 Improvement, methodology, query or suggestion
 0 ALERT type 5 Informative message, check
```

It is advisable to attempt to resolve as many as possible of the alerts in all categories. Often the minor alerts point to easily fixed oversights, errors and omissions in your CIF or refinement strategy, so attention to these fine details can be worthwhile. In order to resolve some of the more serious problems it may be necessary to carry out additional measurements or structure refinements. However, the purpose of your study may justify the reported deviations and the more serious of these should normally be commented upon in the discussion or experimental section of a paper or in the "special_details" fields of the CIF. checkCIF was carefully designed to identify outliers and unusual parameters, but every test has its limitations and alerts that are not important in a particular case may appear. Conversely, the absence of alerts does not guarantee there are no aspects of the results needing attention. It is up to the individual to critically assess their own results and, if necessary, seek expert advice.

Publication of your CIF in IUCr journals

A basic structural check has been run on your CIF. These basic checks will be run on all CIFs submitted for publication in IUCr journals (*Acta Crystallographica*, *Journal of Applied Crystallography*, *Journal of Synchrotron Radiation*); however, if you intend to submit to *Acta Crystallographica Section C* or *E* or *IUCrData*, you should make sure that full publication checks are run on the final version of your CIF prior to submission.

Publication of your CIF in other journals

Please refer to the *Notes for Authors* of the relevant journal for any special instructions relating to CIF submission.

PLATON version of 20/01/2022; check.def file version of 19/01/2022

Datablock EMW-3 - ellipsoid plot

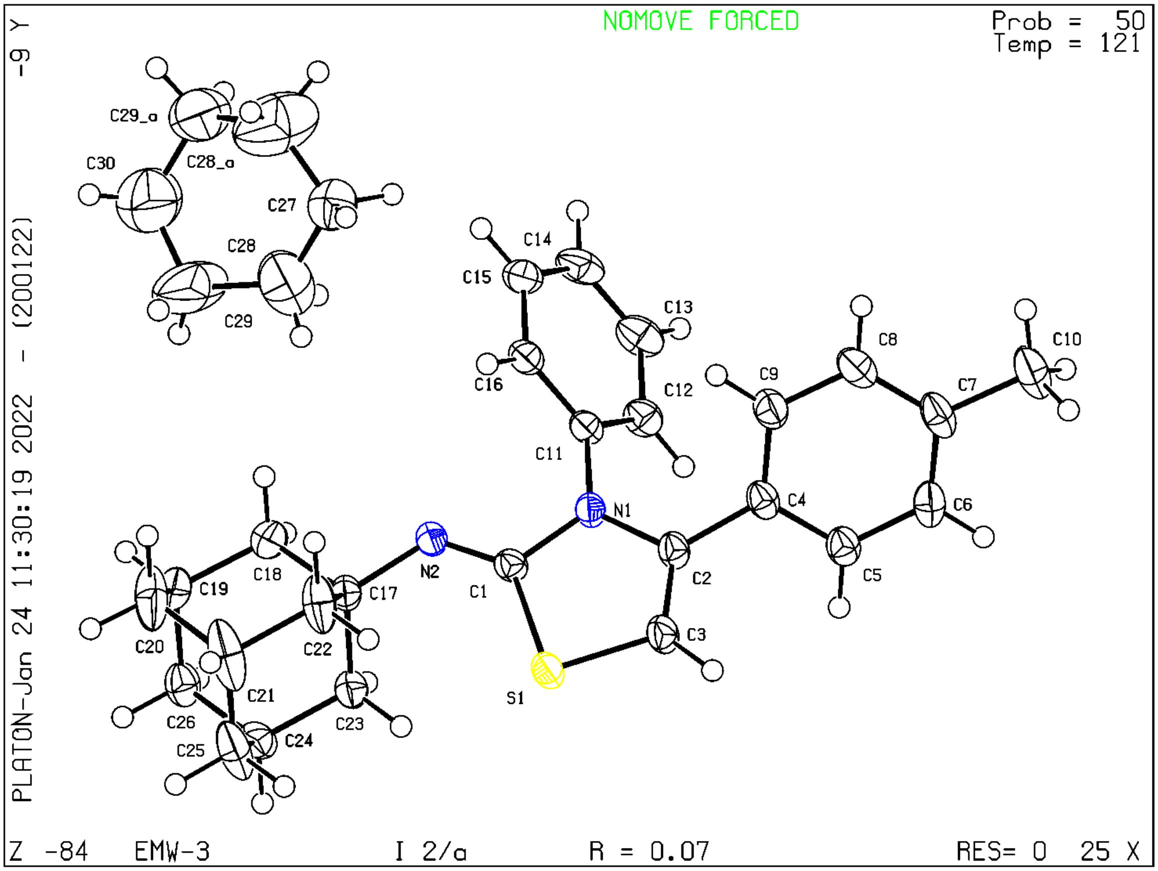

checkCIF/PLATON report

Structure factors have been supplied for datablock(s) EMW-4

THIS REPORT IS FOR GUIDANCE ONLY. IF USED AS PART OF A REVIEW PROCEDURE FOR PUBLICATION, IT SHOULD NOT REPLACE THE EXPERTISE OF AN EXPERIENCED CRYSTALLOGRAPHIC REFEREE.

No syntax errors found.   CIF dictionary   Interpreting this report

Datablock: EMW-4

Bond precision:	C-C = 0.0046 A		Wavelength=1.54184
Cell:	a=7.1421(5)	b=11.6290(8)	c=15.1229(12)
	alpha=107.993(7)	beta=99.633(6)	gamma=95.238(6)
Temperature:	329 K		

	Calculated	Reported
Volume	1164.00(16)	1163.99(16)
Space group	P -1	P -1
Hall group	-P 1	-P 1
Moiety formula	C29 H28 N2 S	C29 H28 N2 S
Sum formula	C29 H28 N2 S	C29 H28 N2 S
Mr	436.59	436.59
Dx,g cm-3	1.246	1.246
Z	2	2
Mu (mm-1)	1.365	1.365
F000	464.0	464.0
F000’	465.77	
h,k,lmax	8,14,18	8,14,18
Nref	4776	4776
Tmin,Tmax	0.906,0.960	0.906,0.960
Tmin’	0.664	

Correction method= # Reported T Limits: Tmin=0.906 Tmax=0.960
AbsCorr = MULTI-SCAN

Data completeness= 1.000   Theta(max)= 74.859

R(reflections)= 0.0542(2873)   wR2(reflections)= 0.1481(4698)

S = 1.013   Npar= 289

The following ALERTS were generated. Each ALERT has the format
test-name_ALERT_alert-type_alert-level.
Click on the hyperlinks for more details of the test.

Alert level C

```
PLAT242_ALERT_2_C Low    'MainMol' Ueq as Compared to Neighbors of        C20 Check
PLAT340_ALERT_3_C Low Bond Precision on  C-C Bonds ...............    0.00465 Ang.
PLAT906_ALERT_3_C Large K Value in the Analysis of Variance ......      4.315 Check
```

Alert level G

```
PLAT066_ALERT_1_G Predicted and Reported Tmin&Tmax Range Identical          ? Check
PLAT883_ALERT_1_G No Info/Value for _atom_sites_solution_primary .     Please Do !
PLAT912_ALERT_4_G Missing # of FCF Reflections Above STh/L=  0.600         78 Note
PLAT941_ALERT_3_G Average HKL Measurement Multiplicity ...........        3.8 Low
PLAT978_ALERT_2_G Number C-C Bonds with Positive Residual Density.          1 Info
```

0 **ALERT level A** = Most likely a serious problem - resolve or explain
0 **ALERT level B** = A potentially serious problem, consider carefully
3 **ALERT level C** = Check. Ensure it is not caused by an omission or oversight
5 **ALERT level G** = General information/check it is not something unexpected

2 ALERT type 1 CIF construction/syntax error, inconsistent or missing data
2 ALERT type 2 Indicator that the structure model may be wrong or deficient
3 ALERT type 3 Indicator that the structure quality may be low
1 ALERT type 4 Improvement, methodology, query or suggestion
0 ALERT type 5 Informative message, check

It is advisable to attempt to resolve as many as possible of the alerts in all categories. Often the minor alerts point to easily fixed oversights, errors and omissions in your CIF or refinement strategy, so attention to these fine details can be worthwhile. In order to resolve some of the more serious problems it may be necessary to carry out additional measurements or structure refinements. However, the purpose of your study may justify the reported deviations and the more serious of these should normally be commented upon in the discussion or experimental section of a paper or in the "special_details" fields of the CIF. checkCIF was carefully designed to identify outliers and unusual parameters, but every test has its limitations and alerts that are not important in a particular case may appear. Conversely, the absence of alerts does not guarantee there are no aspects of the results needing attention. It is up to the individual to critically assess their own results and, if necessary, seek expert advice.

Publication of your CIF in IUCr journals

A basic structural check has been run on your CIF. These basic checks will be run on all CIFs submitted for publication in IUCr journals (*Acta Crystallographica*, *Journal of Applied Crystallography*, *Journal of Synchrotron Radiation*); however, if you intend to submit to *Acta Crystallographica Section C* or *E* or *IUCrData*, you should make sure that full publication checks are run on the final version of your CIF prior to submission.

Publication of your CIF in other journals

Please refer to the *Notes for Authors* of the relevant journal for any special instructions relating to CIF submission.

PLATON version of 18/12/2021; check.def file version of 18/12/2021

Datablock EMW-4 - ellipsoid plot

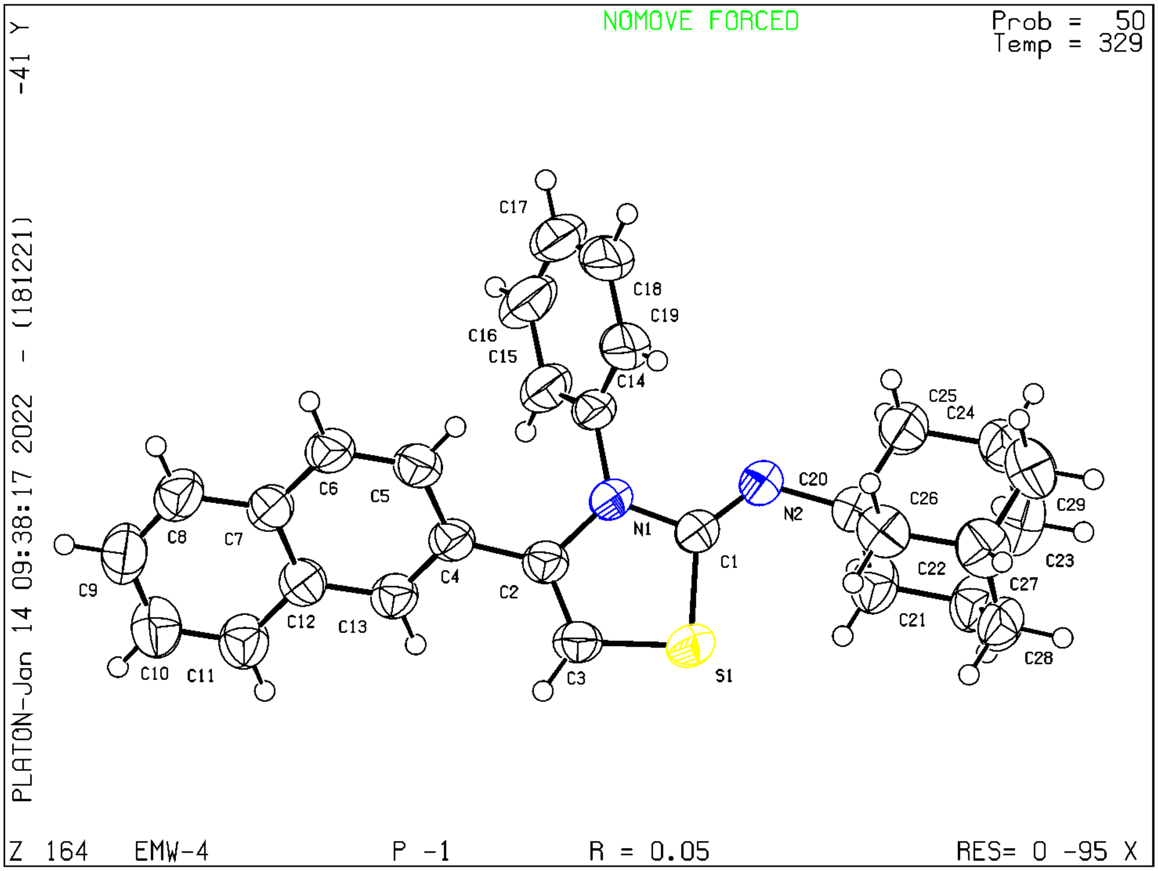

Supplement: Supplementary file 1 — Supplementary Information. [file 41598_2022_25390_MOESM1_ESM.pdf]
